# Reactivity of Diamines in Acyclic Diamino Carbene Gold Complexes

**DOI:** 10.1021/acs.inorgchem.2c00509

**Published:** 2022-05-04

**Authors:** Guilherme M. D. M. Rúbio, Tristan T. Y. Tan, Alexander Prado-Roller, Jia Min Chin, Michael R. Reithofer

**Affiliations:** † Institute of Inorganic Chemistry, Faculty of Chemistry, University of Vienna, Vienna A-1090, Austria; ‡ Institute of Materials Research and Engineering, A*STAR (Agency for Science, Technology and Research), Singapore 138634, Singapore; § Institute of Inorganic Chemistry - Functional Materials, Faculty of Chemistry, University of Vienna, Vienna A-1090, Austria

## Abstract

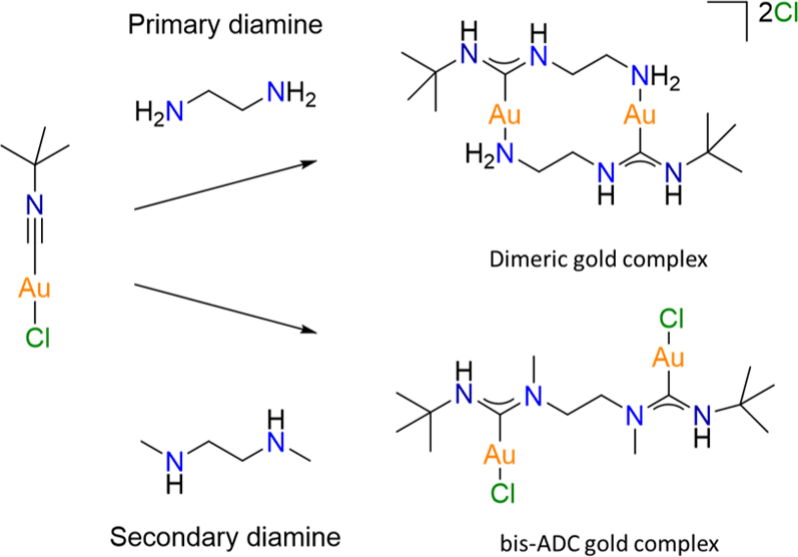

Acyclic diamino carbenes (ADCs) are interesting alternatives to their more widely studied N-heterocyclic carbene counterparts, particularly due to their greater synthetic accessibility and properties such as increased sigma donation and structural flexibility. ADC gold complexes are typically obtained through the reaction of equimolar amounts of primary/secondary amines on gold-coordinated isocyanide ligands. As such, the reaction of diamine nucleophiles to isocyanide gold complexes was expected to lead to bis-ADC gold compounds with potential applications in catalysis or as novel precursors for gold nanomaterials. However, the reaction of primary diamines with two equivalents of isocyanide gold chlorides resulted in only one of the amine groups reacting with the isocyanide carbon. The resulting ADC gold complexes bearing free amines dimerized via coordination of the amine to the partner gold atom, resulting in cyclic, dimeric gold complexes. In contrast, when secondary diamines were used, both amines reacted with an isocyanide carbon, leading to the expected bis-ADC gold complexes. Density functional theory calculations were performed to elucidate the differences in the reactivities between primary and secondary diamines. It was found that the primary amines were associated with higher reaction barriers than the secondary amines and hence slower reaction rates, with the formation of the second carbenes in the bis-ADC compounds being inhibitingly slow. It was also found that diamines have a unique reactivity due to the second amine serving as an internal proton shuttle.

## Abstract 2

Reactions between primary diamines with a gold-coordinated isocyanide complex afforded unexpected cyclic dimeric gold complexes instead of bis-acyclic diamino carbene gold complexes, which were obtained when using secondary diamines. DFT calculations revealed that primary amines react slower in the carbene-forming reaction, with the barrier of the second carbene formation ultimately resulting in this interesting selectivity.

## Introduction

Complexes of acyclic diamino carbenes (ADCs) are amongst the most synthetically accessible carbene complexes. However, they are significantly under-investigated compared to the ubiquitous complexes of N-heterocyclic carbenes (NHCs). NHCs’ attractive properties of high thermal and chemical stabilities allow their complexes to challenge phosphine-based metal catalysts in a number of organic transformations, most commonly for C–C cross-coupling reactions.^1^ However, synthetically adjusting NHCs’ electronic and steric properties is relatively challenging, particularly for the synthesis of asymmetric or chiral NHCs, thereby limiting the tunability and versatility of these ligands.^2−4^ In contrast, ADCs offer some benefits over NHCs; first, ADCs have a wider N–C–N bond angle and an accordingly higher steric impact on the metal center.^5^ Consequently, this can lead to increased complex stability and also promote reductive eliminations during cross-coupling reactions.^6,7^ On the other hand, ADCs are better σ-donor ligands compared to NHCs, leading to an increased electronic density at the metal center, which should help to facilitate oxidative addition reactions.^5,8^ On that note, in a recent study, Huynh and co-workers systematically investigated the donor properties of a series of ADCs and concluded that protic ADCs are stronger sigma donors than classical NHCs.^9^ Furthermore, ADCs are structurally flexible due to an often very low rotational barrier of the C–N bond.^10^ Values of a C–N rotational barrier of lower than 13 kcal/mol have been reported,^10,11^ allowing ADC metal complexes to easily adapt to structural changes required in different stages of a catalytic process. Finally, as protic ADCs are typically synthesized through the reaction of an isocyanide metal complex with a primary or secondary amine, each N-substituent can be individually selected based on the isocyanide and amine used, thus allowing fine-tuning of ADC steric and electronic properties.^12−15^

Especially in recent years, diverse ADC gold chloride compounds have been explored in catalysis^16^ for biological applications and as gold nanomaterial precursors.^15,17−19^ There are several examples of the reaction of isocyanide gold chloride with primary and secondary monoamines but only a few reports on the reactions of diamines with isocyanide gold chloride thus far. Such reactions are worth investigating as the expected products of bis-carbene complexes are not only advantageous for catalysis but could also serve as precursors for bis-carbene-stabilized nanomaterials, which may offer enhanced stability. Espinet and co-workers reported the reaction of [AuCl(CNPy-2)] with chiral diamines, namely (*S*)-1,1′-binapthyl-2,2′-diamine, (1*R*,2*R*)-1,2-diaminecyclohexane, and (1*R*,2*R*)-1,2-diphenylethane-1,2-diamine, where the diamine bridges two gold ADC complexes when reacted in a 2:1 ratio (isocyanide gold chloride/diamine).^20^ Similar reactions are also reported by Toste et al. using 3,3′-substituted 1,1′-binapthyl-2,2′-diamine, again yielding bridged ADC gold complexes,^21^ and recently, Gimeno and co-workers reported on the equimolar reaction of isocyanide gold chloride with 1,2-diaminecyclohexane yielding a mono ADC complex which still contains a free amine group.^22^

Herein, we report on the reactivity of isocyanide gold chloride with primary and secondary diamines. While the reaction of secondary diamines with isocyanide gold chloride yields the expected bis-ADC gold complex, to our surprise, the corresponding reaction with primary diamines results instead in the self-assembly of cyclic gold dimers. To understand the differences in the reactivities between primary and secondary diamines, density functional theory (DFT) calculations were therefore performed.

## Results and Discussion

### Synthesis of Complexes [**1–5**]

**Scheme 1:**
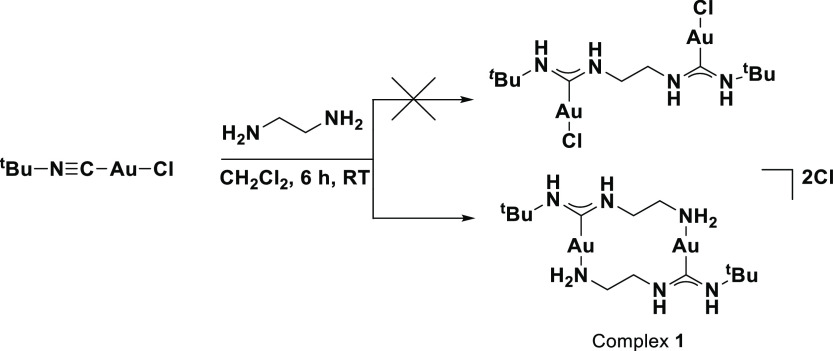
Reaction of Ethylenediamine with [*t*-BuNCAuCl]

ADC gold complexes are typically obtained through the reaction of equimolar amounts of primary/secondary amines with coordinated isocyanide ligands. We therefore expected that the reaction of protic diamine nucleophiles with two equivalents of isocyanide gold chloride would lead to the formation of bis-ADC gold compounds where the employed diamine “bridges” the two gold centers. When two equivalents of [*t*-BuNCAuCl] were reacted with one equivalent of ethylenediamine in dichloromethane (DCM) under ambient conditions, an almost immediate formation of a white precipitate was observed, which could be isolated at 49% yield (Scheme 1). Detailed structural investigation revealed that the white precipitate has a mass of *m*/*z* = 715.1850 for [M – Cl^–^]^+^ and *m*/*z* = 340.1081 for [M – 2Cl^–^]^2+^, respectively. The analysis of the ^1^H NMR spectrum clearly showed the presence of two independent CH_2_ groups with resonances at 3.21 and 4.15 ppm corresponding to the two CH_2_ groups of the ethylenediamine residue, indicating that the resulting complex lacks a *C*_2_ symmetry through the ethylenediamine ligand. Furthermore, ^1^H and ^15^N-HMBC NMR spectra (externally referenced to NH_3_) showed three ^15^N signals at 16.4, 121.1, and 151.3 ppm. The ^15^N signals at 121.1 and 151.3 ppm are assigned to the nitrogen atoms within the ADC, whereas the chemical shift of the third nitrogen at 16.4 ppm indicates that no second ADC was formed. Based on the spectroscopic evidence, we concluded that complex **1** was indeed obtained. This was further supported by repeating the reaction with equimolar amounts of [*t*-BuNCAuCl] and ethylenediamine, where **1** was obtained at 98% yield.

**Scheme 2:**
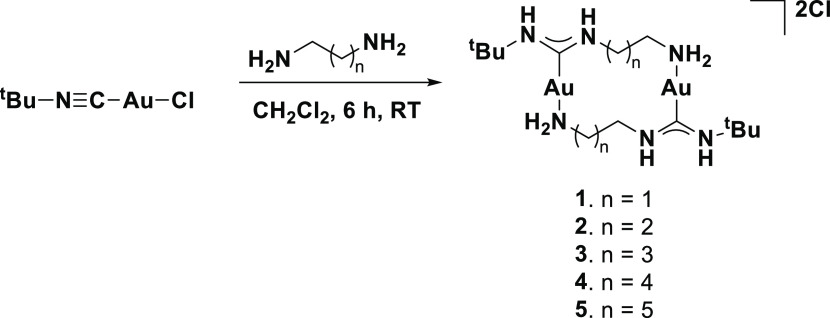
Reaction between Various Primary Diamines and [*t*-BuNCAuCl]

We then investigated if such dimeric gold complexes can also be formed using diamines spaced by longer hydrocarbon chains. When ethylenediamine was replaced with either 1,3-propylenediamine, 1,4-butanediamine, 1,5-pentanediamine, or 1,6-hexanediamine, again a white precipitate was formed each time (Scheme 2). The formation of dimeric complexes can be easily confirmed via mass spectrometric analysis through the presence of two distinct molecular mass peaks which arise from [M – Cl^–^]^+^ or [M – 2Cl^–^]^2+^ (Supporting Information, Figures S23–S30). Here, the charge of the molecular species can be assigned based on the isotopic distribution, where a mass difference between the isotope peaks of *m*/*z* = 1 is found for a singly charged species and a *m*/*z* = 0.5 for a doubly charged species. Furthermore, the isotope distribution modeling clearly shows that in the [M – 2Cl^–^]^2+^ signal, both chlorides are missing, which is expected, given that all dimers are doubly charged with chlorides as counter ions.

**Figure 1:**
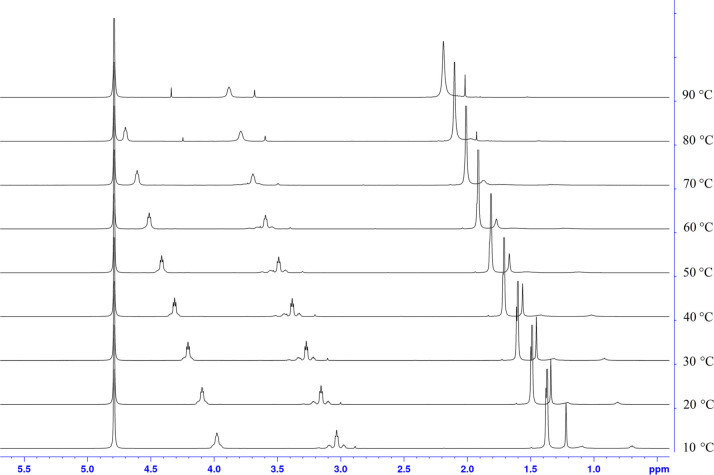
^1^H NMR variable temperature experiment (VT from 10 to 90 °C) of complex **1**.

It is well-documented that, depending on the steric demand of the N-nitrogen substituents, ADC complexes can form stable stereoisomers or rotamers in solution at room temperature.^17^ As such, NMR analysis can be complicated as each rotamer gives rise to a single set of signals. The formation of rotamers is clearly visible in the ^1^H NMR spectrum of **1,** where three signals for each CH_2_ group (e.g., RNHCH_2_ = 3.27, 3.21, and 3.15 ppm at 25 °C) are detected, with one main isomer at 3.21 ppm and two further isomers at 3.27 and 3.15 ppm with similar concentrations. In order to determine that additional peaks are arising from rotamers and are not compound impurities, VT NMR studies were carried out (Figure 1). All signals show a significant low field shift with increasing temperature, while at the same time, the intensity of the minor rotamers decreases. This can be especially observed at the CH_2_ groups at RNHCH_2_ = 3.27, 3.21, and 3.15 ppm (at 25 °C). With increasing temperature, the intensity of the two small signals not only shows a low field shift but also at about 80 °C, only one signal at 3.72 ppm is visible, thus confirming that indeed rotamers are present at RT; similar shifts are observed for complex **4** (Figure S12).

**Figure 2:**
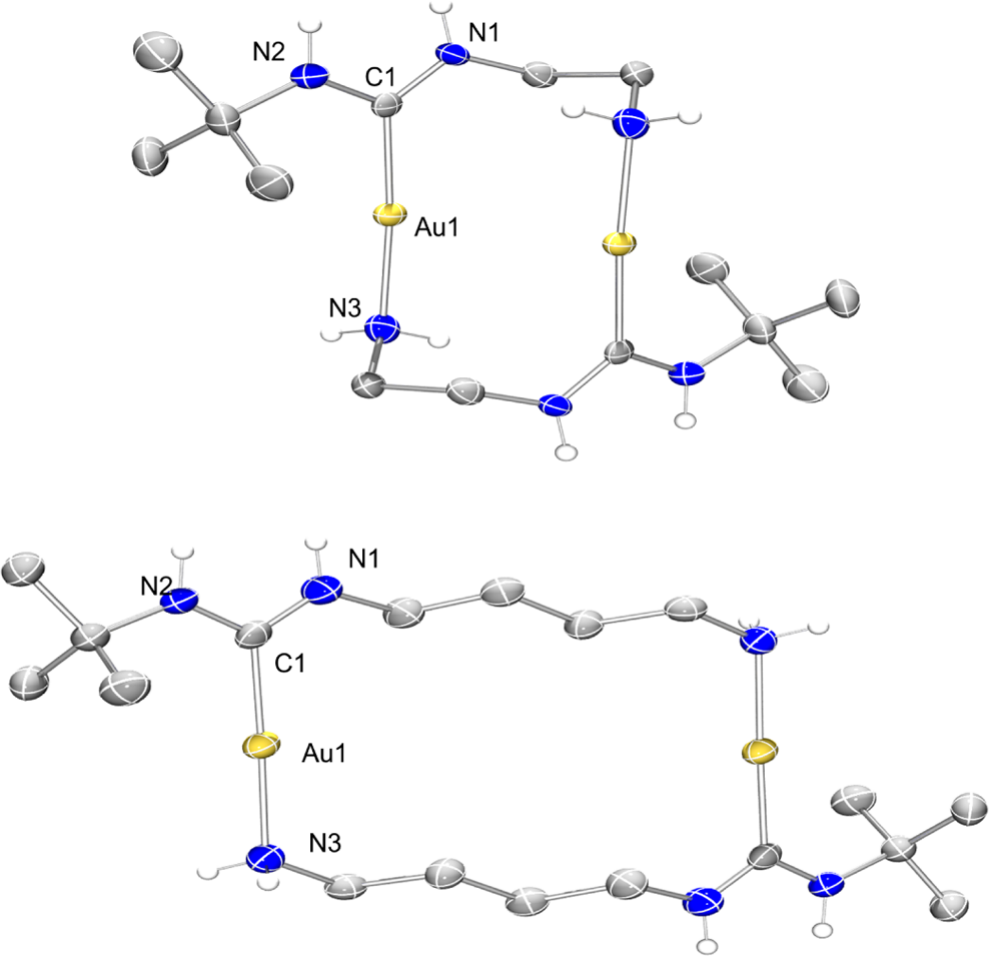
Molecular structures (ellipsoids drawn at 50% probability) of complexes **1** (top) and **3** (bottom). Hydrogen atoms (except for NH), co-crystallized solvents, and counterions have been omitted for clarity. Selected bond lengths [Å] and bond angles [°] for **1**: Au1–Au1 3.1539(5), Au1–N3 2.109(11), Au1–C1 1.989(13); C1–Au1–N3 174.2(5), N1–C1–N2 115.4(11). Selected bond lengths [Å] and bond angles [°] for **3**: Au1–C1 2.007(4), Au1–N3 2.074(3); C1–Au1–N3 177.84(13), N2–C1–N1 115.3(3).

In addition to the spectroscopic evidence for the dimeric nature of complexes **1–5**, the structures of complexes **1** and **3** were unambiguously determined by single crystal X-ray diffraction. Vapor diffusion of Et_2_O into saturated ethanol solutions of the respective complexes yielded single crystals of composition **1** and **3**·EtOH. The molecular structures are shown in Figure 2. The Au–C_ADC_ bond length in complexes **1** and **3** measures to be 1.992(11) and 2.007(4) Å, respectively, which falls in the typical range for Au–C_ADC_ distances reported previously.^14^

### Synthesis of Complexes [**6–9**]

**Scheme 3:**
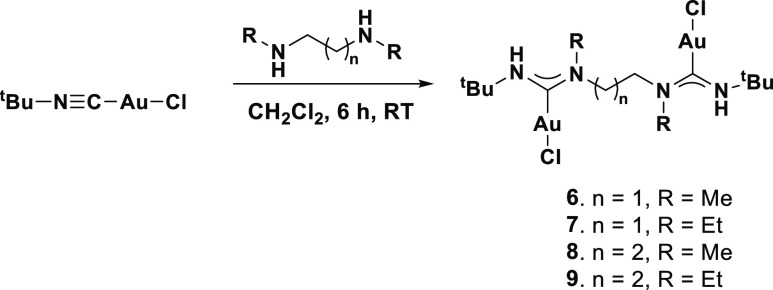
Synthesis of bis-Acyclic Diamino Carbene Gold Complexes

It has been previously reported that secondary amines are more reactive than primary amines, leading to shorter reaction times.^23,24^ As such, we thought to investigate if the reaction with secondary diamines would also yield the same reaction products as primary diamines. When one equivalent of *N*,*N*′-dimethylethylenediamine was reacted with two equivalents of [*t*-BuNCAuCl] in DCM, no precipitate was formed even after 24 h. A white powder could only be isolated after *n*-pentane was added, suggesting that the reaction did not yield a charged dimeric compound. MS analysis showed a main signal at *m*/*z* = 683.1481 which could be identified as [M – Cl^–^]^+^, while the analysis of the ^1^H NMR spectra revealed only one CH_2_ (4.19 ppm) and one CH_3_ signal (3.15 ppm), indicating that the obtained compound has a *C*_2_ symmetry through the ligand. Furthermore, ^1^H and^15^N-HMBC NMR spectra (externally referenced to NH_3_) showed only two ^15^N signals at 151.5 and 119.8 ppm, respectively, which can be both assigned to the ADC nitrogen atoms. Based on the spectroscopic evidence, we concluded that the bis-carbene complex **6** (Scheme 3) was formed, which is in clear contrast to the product isolated from the reaction with primary diamines. Furthermore, the reaction of equimolar amounts of *N*,*N*′-dimethylethylenediamine and [*t*-BuNCAuCl], as well as changing the solvent to a less polar one, still only yields complex **6**, indicating that its formation is highly favored.

When the same reaction was performed using either *N*,*N*′-diethylethylenediamine, *N*,*N*′-dimethylpropyldiamine, or *N*,*N*′-diethylpropyldiamine in DCM, again no precipitate was observed. The formation of a bis-carbene complex can be easily identified via mass spectrometric analysis through the characteristic [M – Cl^–^]^+^ signal. Furthermore, the proposed structure can also be confirmed by NMR spectroscopy. In the ^1^H NMR spectrum, the NH signal for compounds **6–9** is detected at around 6 ppm, while alkyl signals are in the expected regions. Again, the presence of rotamers is visible through an additional signal of the ^*t*^Bu residue (see e.g., Figure S17). However, the relative amounts of rotamers are lower when compared to compounds **1–5**, and only one rotamer can be seen through the additional ^*t*^Bu signal. Furthermore, the ^13^C NMR resonances of the C_ADC_ atoms for all complexes (dimers and bis-carbenes) were detected between 189.9 and 192.8 ppm, respectively, which are in accordance with the similar reported compounds.^9,15,25^ Interestingly, the trans-standing ligand (amine vs chloride) has little influence on the chemical shift of the carbene signal.

**Figure 3:**
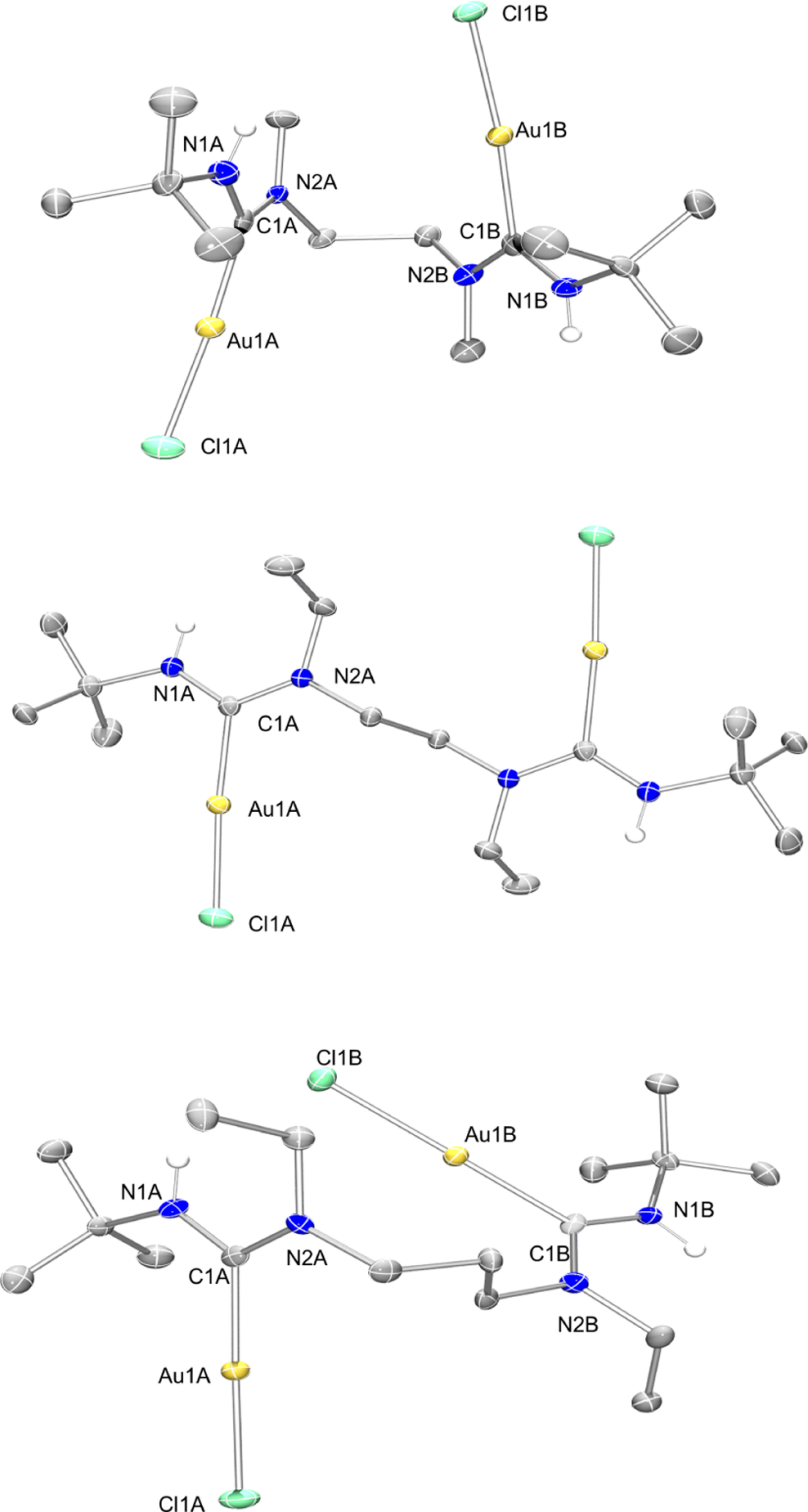
Molecular structures (ellipsoids drawn at 50% probability) of complexes **6** (top), **7** (middle), and **9** (bottom). Hydrogen atoms (except for NH) and co-crystallized solvents have been omitted for clarity. Selected bond lengths [Å] and bond angles [°] for **6**: Au1A–C1A 2.018(6), Au1B–C1B 2.008(6), Au1A–Cl1A 2.3117(15), Au1B–Cl1B 2.2896(15); C1A–Au1A–Cl1A 175.15(16), C1B–Au1B–Cl1B 176.56(16), N1A–C1A–N2A 117.4(5), N1B–C1B–N2B 115.6(5). Selected bond lengths [Å] and bond angles [°] for **7**: Au1A–C1A 2.017(3). Au1B–C1B 2.012(3), Au1A–Cl1A 2.2989(8), Au1B–Cl1B 2.2878(8); C1A–Au1A–Cl1A 176.17(9) C1B–Au1B–Cl1B 173.69(9), N1A–C1A–N2A 117.5(3), N1B–C1B–N2B 117.6(3). Selected bond lengths [Å] and bond angles [°] for **9**: Au1A–C1A 2.011(6), Au1A–Cl1A 2.2895(15), C1A–Au1A–Cl1A 178.63(17).

Single crystals suitable for X-ray diffraction of complexes **6**, **7,** and **9** allowed for their unambiguous structural determination. Vapor diffusion of Et_2_O into saturated DCM solutions of the respective complexes yielded single crystals of compositions **6**, **7,** and **9**. The structures are depicted in Figure 3. The nature of the used secondary amine had little effect on the Au–C_ADC_ bond lengths. This observation agrees with the ^13^C NMR spectroscopic data, which showed little variations in the chemical shifts of the C_ADC_ atoms between complexes, implying that the donor strength of various ADCs is not affected strongly by the nature of the R groups on the nitrogen atoms.

### Computational Results and Discussion

To understand the differences in the reactivities between primary and secondary diamines, DFT calculations were performed to model the potential energy surfaces of the reactions. We employed the B2PLYP//BP86 protocol as outlined by Ciancaleoni and co-workers.^26^

**Scheme 4:**
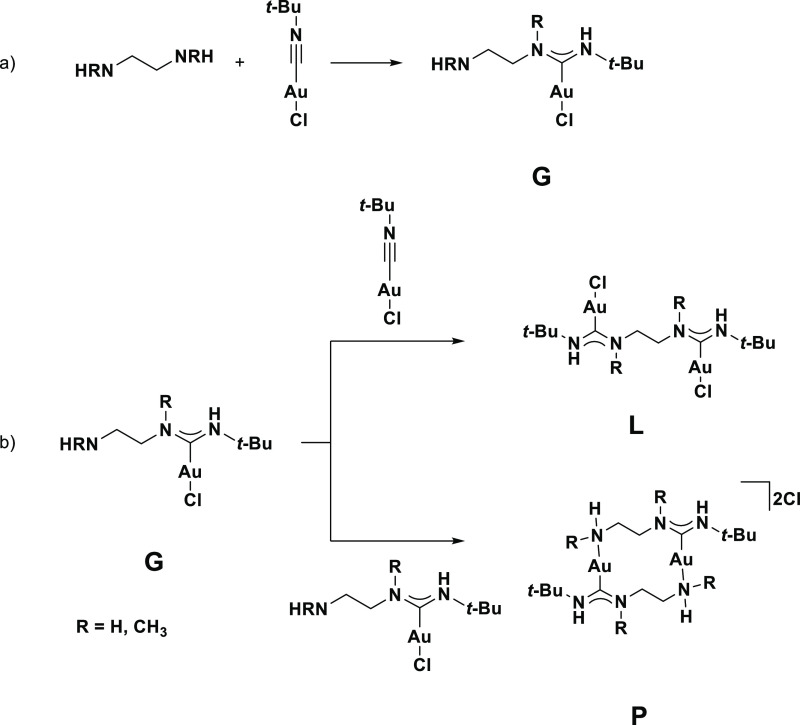
(a) Formation of the Mono-Carbene Complex (b) Divergent Reactivity of the Second Amine Group Either Forming the Bis-Carbene Complex (Top) or Cyclic Dimer (Bottom)

Focusing on the ethylene-bridged diamines, we first studied the formation of the mono-carbene complexes **G1** (R = H) and **G2** (R = CH_3_) (Scheme 4a) via the nucleophilic attack of one amine group on the isocyanide carbon atom, followed by a proton transfer.

**Figure 4:**
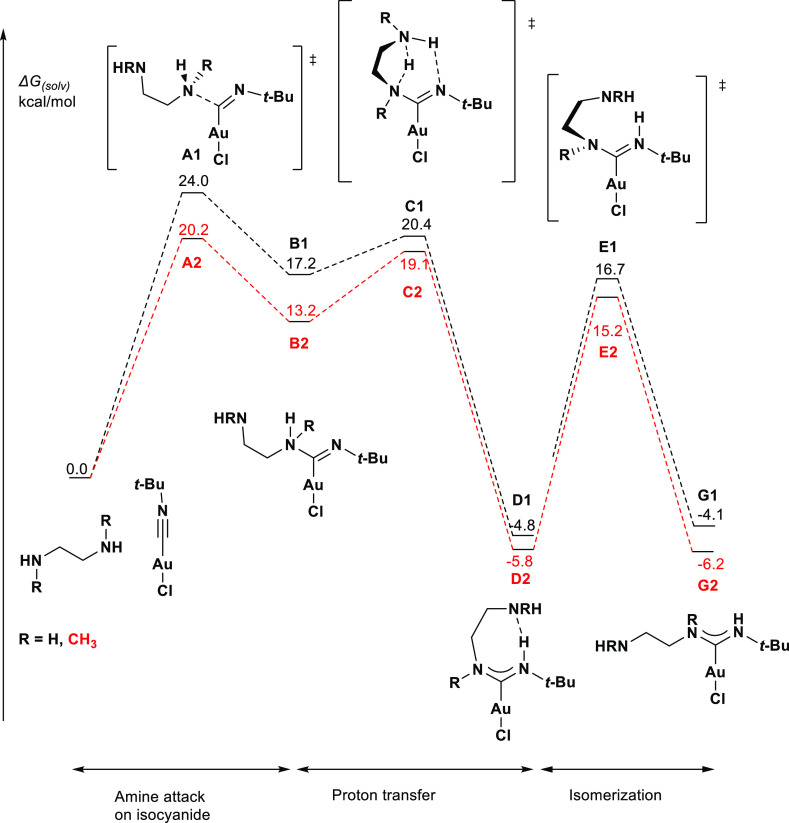
Reaction profile of the formation of the mono-carbene species.

We would like to highlight two sets of noteworthy findings from this reaction profile (Figure 4). First is the higher reaction barrier associated with the primary amine (**A1**, Δ*G*_(solv)_ = 24.0 kcal/mol) than with the secondary amine (**A2**, Δ*G*_(solv)_ = 20.2 kcal/mol), which is consistent with the observation that the formation of ADC complexes is slower with primary amines than with secondary amines.^14^

The second set of noteworthy findings relates to the transition states **C1** and **C2**, where the second amine functional group serves as an internal proton shuttle, allowing the proton transfer step to proceed with very low step-barriers. Without the second amine group, the proton transfer would have to go through a very unfavorable four-membered ring transition state (**F1** and **F2**, see Supporting Information Figures S48 and S49 for the full profile), or an external proton shuttle such as excess amine or adventitious water could facilitate the proton transfer.^27^ Either of these alternate pathways would result in a higher overall barrier.

Finally, isomerization of the syn*,*anti isomers **D1** and **D2** to the syn,syn isomers **G1** and **G2** proceeds quickly over a low step barrier of about 16 kcal/mol.

We next studied the two possible reaction profiles involving the free amine ends of the mono-carbene complexes **G1** and **G2** (see Figure 4). Reaction with a second equivalent of [*t*-BuNCAuCl] would yield the bis-carbene species and complexes **L1** and **L2**. Alternatively, reaction with the Au center of another mono-carbene complex and displacement of the chloride ligand would yield the dimeric, dicationic species **P1** and **P2**.

Experimentally, reactions involving primary diamines exclusively followed the dimerization pathway to give analogues of the dimer **P1**, while reactions using secondary diamines displayed selectivity for the bis-carbene complexes analogous to complex **L2**. The analysis of the reaction profile reveals a possible explanation for such selectivity.

**Figure 5:**
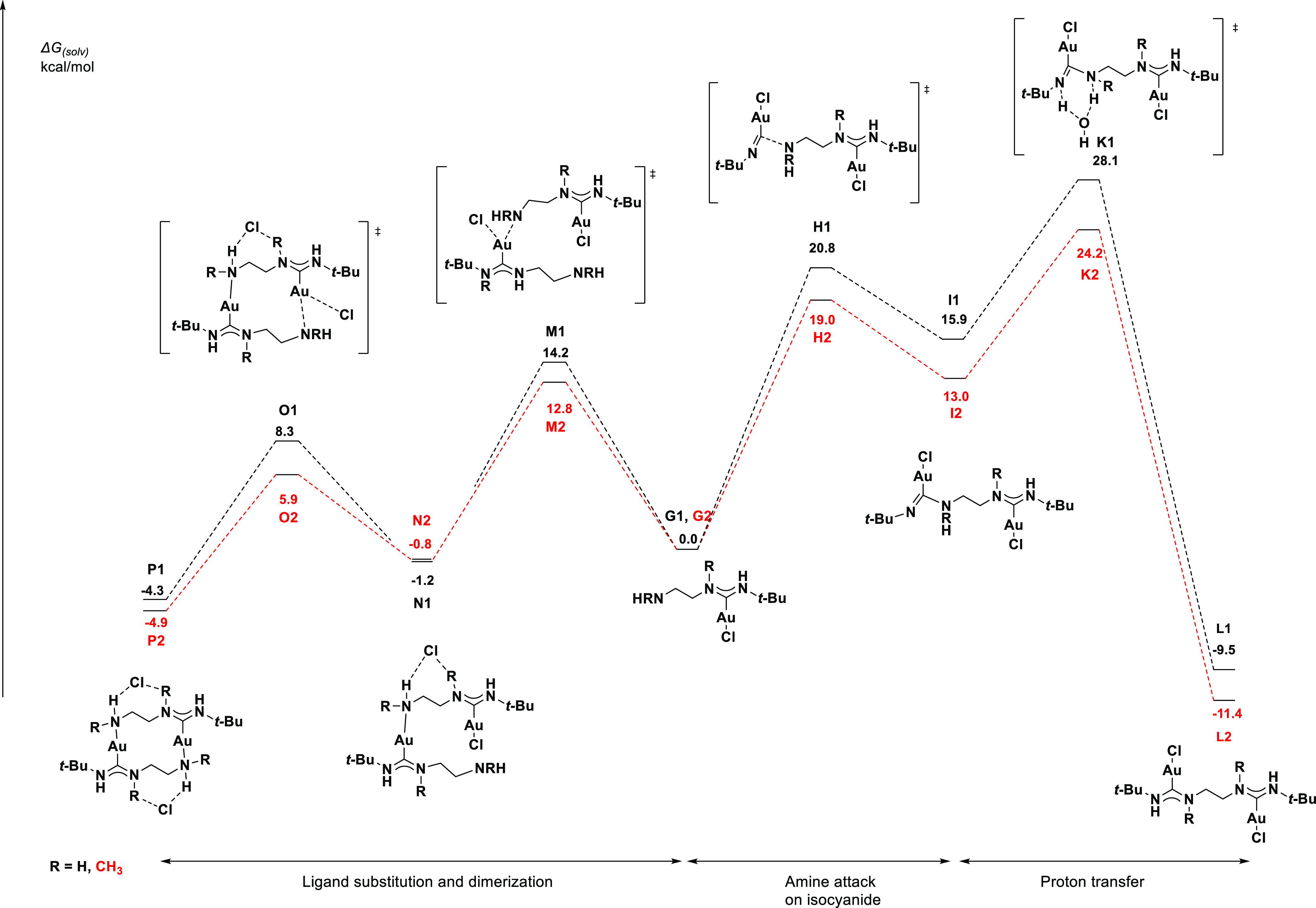
Profile of the two possible reactions pathways of the amine functional group of **G1** and **G2**.

We first analyze the reaction path toward the bis-carbene complex. Like the reaction profile shown in Figure 5 for the formation of the mono-carbene complex, reactions involving secondary amines have lower barriers than those of the primary amines. However, a key difference between the formation of the first carbene and the second carbene is that the latter lacks an internal proton shuttle. We have modeled the transition state with water as the proton shuttle (**K1** and **K2**) and found that the higher overall barrier associated with the primary amine (28.1 kcal/mol) implied requiring a few days for the formation of **L1**, while the lower overall barrier associated with the secondary amine (24.2 kcal/mol) meant that obtaining **L2** in a few hours at room temperature was feasible.

The analysis of the pathway for the dicationic dimer formation revealed very low barriers for both the primary and secondary amines. The poor solubility of the dimer **P1** in DCM results in precipitation of the complex, and the energy of crystallization likely drives forward the reaction. The dimer **P2** appears to be more soluble in DCM, and the micro reversibility of the dimerization in solution leads to the formation of the eventual thermodynamic product **L2**. We note that the relative energies of the ionic dimers should be interpreted qualitatively due to the large charge separation compared to the neutral complexes, which might not be modeled sufficiently or accurately by the used solvation model (see Experimental Section for details).

## Conclusions

The reactivity difference of primary and secondary diamines with isocyanide gold was investigated. First, we studied the reaction of isocyanide gold with 0.5 equivalents of ethylene diamine with the expectation that a bis-carbene complex would be formed. Interestingly, the formation of a bis-carbene was not observed, but instead, the self-assembly of a cyclic gold dimer was observed. The same reaction products could be observed when a molar ratio of 1:1 (isocyanide gold/ethylene diamine) or diamines with different chain lengths are used. When the same reaction was performed using secondary amines, only the bis-carbene gold complex could be obtained. To understand the differences in the reactivities between primary and secondary diamines, DFT calculations were performed, revealing that the primary amine has a higher reaction barrier when forming the ADC. Furthermore, the analysis of the pathway for the dicationic dimer formation revealed very low barriers for both the primary and secondary amines; however, the reaction production of the primary amine has very low solubility in the used solvent DCM, which favors the dimer formation over the bis-carbene compounds. Overall, this study shows that the product selectivity of primary versus secondary opens up new routes to ADC compounds, which might find application in catalysis and as gold nanomaterial precursors.^18^

## Experimental Section

### Materials and Methods

All experiments were performed under ambient conditions unless stated otherwise. 1,2-Diaminoethane, 1,4-diaminobutane, 1,5-diaminopentane, *N*,*N′*-dimethylethylenediamine, *N*,*N*′-diethylethylenediamine, *N*,*N*′-dimethyl-propanediamine, *N*,*N*′-diethyl-propanediamine, and *n*-pentane were purchased from Sigma-Aldrich, 1,3-diaminopropane and 1,6-diaminohexane were purchased from Alfa Aesar. DCM, Et_2_O, and *n*-hexane were purchased from VWR chemicals. All purchased chemicals were used as received. The [*t*-BuNCAuCl] complex was synthesized according to the literature procedure.^14^

^1^H and ^13^C NMR spectra were recorded at the NMR Center, Faculty of Chemistry, University of Vienna, with a Bruker 600 MHz spectrometer. Chemical shifts (δ) are expressed in ppm downfield from SiMe_4_ using the residual protonated solvent signal as an internal standard. Coupling constants (*J*) are expressed in hertz (Hz). TopSpin 4.0.8 was used to analyze the NMR spectra.

High-resolution MS was measured at the Mass Spectrometry Center, Faculty of Chemistry, University of Vienna, utilizing a Bruker maXis UHR-TOF with electrospray ionization as the ion source. Elemental analysis was determined by the Laboratory for Elemental Analysis, Faculty of Chemistry, University of Vienna, on a PerkinElmer 2400 CHN Elemental Analyzer.

X-ray diffraction data were measured on a Bruker D8 Venture diffractometer equipped with a multilayer monochromator, a Mo Kα INCOATEC micro focus sealed tube, and an Oxford cooling system. Semi-empirical multiscan absorption corrections were applied to all data sets.^28,29^ Structure solutions were found with SHELXS (direct methods)^30^ or Olex2 (charge flipping)^31^ and were refined with SHELXL^32^ against |*F*^2^| of all data using first isotropic and later anisotropic thermal parameters for all non-hydrogen atoms. Non-hydrogen atoms were refined with anisotropic displacement parameters. Hydrogen atoms were added to the structure models at calculated positions.

### General Procedure for the Synthesis of (N^*t*^Bu)(NC_*x*_H_*y*_)CAuCl Dimers

[*t*-BuNCAuCl] (49 mg, 0.156 mmol, 1 equiv) was dissolved in 1 mL of DCM. 0.95 equivalents of a primary diamine were added to the reaction mixture. The reaction mixture was stirred for 6 h at 22 °C. Subsequently, in the cases where the crude product precipitated, filtration was performed, followed by washing three times with *n*-pentane. In the cases where the product showed a slight solubility in DCM, the mixture was concentrated under reduced pressure, followed by the addition of 1 mL of cold Et_2_O, and continued stirring for an additional 15 min. *n*-pentane was then added in a small amount (200 μL), and the resulting precipitate was filtered and washed three times with a 70:30 Et_2_O/*n*-pentane mixture. In both cases, the obtained white precipitate was dried under vacuum overnight and analyzed without further purification.

#### Complex **1**

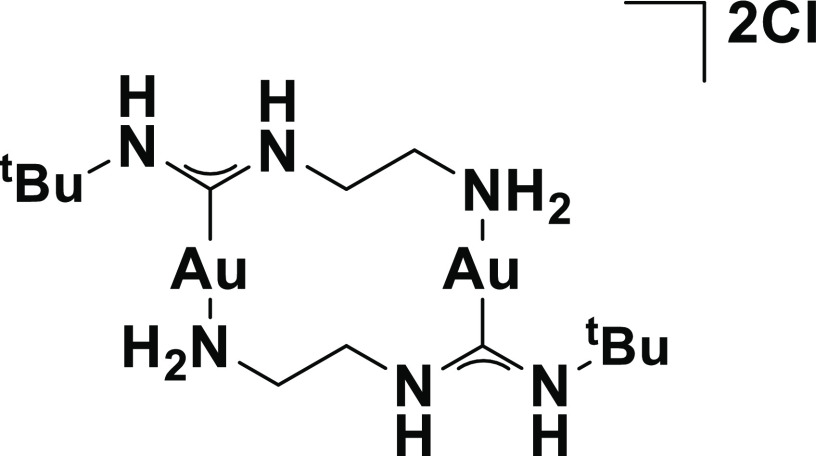



Yield: 107.7 mg (92%). ^1^H NMR (600 MHz, D_2_O): δ 1.38–1.58 (18H, C(C***H***_3_)_3_); 3.21 (4H, CNHC***H***_2_CH_2_); 4.15 (4H, CNHCH_2_C***H***_2_NH_2_) ppm. ^13^C NMR (150 MHz, D_2_O): δ 30.5 (C(***C***H_3_)_3_); 44.3 (CNH***C***H_2_CH_2_); 51.3 (CNHCH_2_***C***H_2_NH_2_); 52.8 (***C***(CH_3_)_3_); 190.8 ((NH)(NH)***C***Au) ppm. MS (*m*/*z*): calcd for [C_14_H_34_Au_2_N_6_Cl]^+^, 715.1859; found [C_14_H_34_Au_2_N_6_Cl]^+^, 715.1850 and [C_14_H_34_Au_2_N_6_]^2+^, 340.1081. Anal. Calcd for C_14_H_34_N_6_Au_2_Cl_2_·2H_2_O: C, 21.36; H, 4.86; N, 10.67. Found: C, 21.23; H, 4.80; N, 10.45.

#### Complex **2**

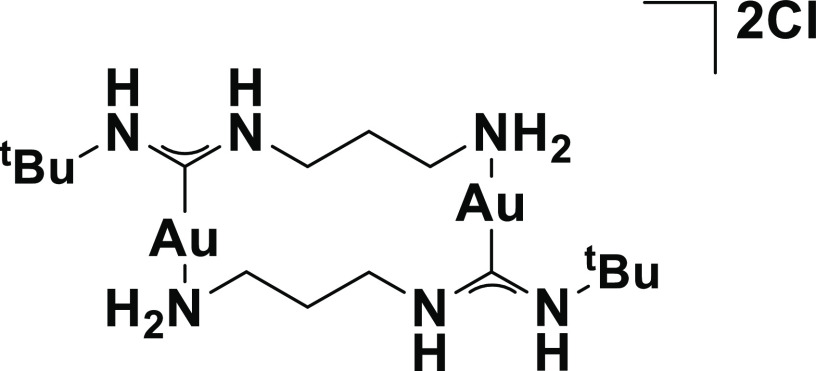



Yield: 99.6 mg (81%). ^1^H NMR (600 MHz, CD_3_OD): δ 1.52–1.67 (18H, C(C***H***_3_)_3_); 1.87–2.20 (4H, CNHCH_2_C***H***_2_CH_2_); 2.95–3.13 (4H, CNHC***H***_2_(CH_2_)_2_NH_2_); 3.59–3.80 (4H, CNH(CH_2_)_2_C***H***_2_NH_2_) ppm. ^13^C NMR (150 MHz, CD_3_OD): δ 30.6 (C(C***H***_3_)_3_); 34.1 (CNHCH_2_C***H***_2_CH_2_); 43.1 (CNH***C***H_2_(CH_2_)_2_NH_2_); 46.9 (CNH(CH_2_)_2_***C***H_2_NH_2_); 52.5 (***C***(CH_3_)_3_); 192.8 ((NH)(NH)**C**Au) ppm. MS (*m*/*z*): calcd for [C_16_H_38_Au_2_N_6_Cl]^+^, 743.2172; found [C_16_H_38_Au_2_N_6_Cl]^+^, 743.2185 and [C_16_H_38_Au_2_N_6_]^2+^, 354.1242. Anal. Calcd for C_16_H_38_Au_2_Cl_2_N_6_·H_2_O: C, 24.10; H, 5.06; N, 10.54. Found: C, 24.33; H, 5.13; N, 10.36.

#### Complex **3**

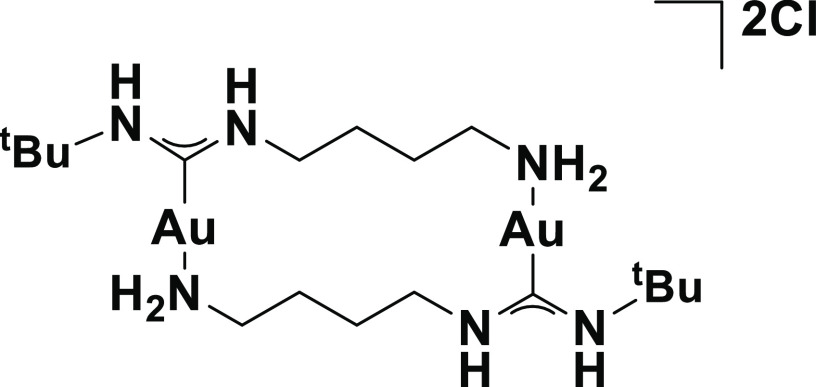



Yield: 89 mg (70%). ^1^H NMR (600 MHz, CD_3_OD): δ 1.50–1.63 (18H, C(C***H***_3_)_3_); 1.72 (4H, CNHCH_2_C***H***_2_(CH_2_)_2_); 1.84 (4H, CNH(CH_2_)_2_(C***H***_2_)CH_2_); 3.04 (4H, CNHC***H***_2_(CH_2_)_3_NH_2_); 3.70 (4H, CNH(CH_2_)_2_C**H**_2_NH_2_) ppm. ^13^C NMR (150 MHz, CD_3_OD): δ 28.1 (CNHCH_2_***C***H_2_(CH_2_)_2_); 29.9 (CNH(CH_2_)_2_***C***H_2_CH_2_); 30.2 (C(***C***H_3_)_3_); 41.3 (CNH***C***H_2_(CH_2_)_3_NH_2_); 49.6 (CNH(CH_2_)_2_***C***H_2_NH_2_); 52.1 (***C***(CH_3_)_3_); 192.6 ((NH)(NH)***C***Au) ppm. MS (*m*/*z*): calcd for [C_18_H_42_Au_2_N_6_Cl]^+^, 771.2485; found [C_18_H_42_Au_2_N_6_Cl]^+^, 771.2472 and [C_18_H_42_Au_2_N_6_]^2+^, 368.1393. Anal. Calcd for C_18_H_42_Au_2_Cl_2_N_6_·CH_3_OH: C, 27.18; H, 5.52; N, 10.01. Found: C, 27.57; H, 5.54; N, 9.81.

#### Complex **4**

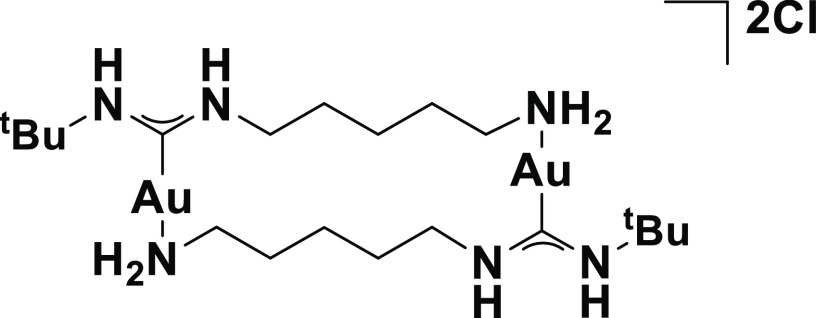



Yield: 102.9 mg (78%). ^1^H NMR (600 MHz, CD_3_OD): δ 1.41–1.51 (4H, CNH(CH_2_)_2_C***H***_2_(CH_2_)_2_); 1.52–1.61 (18H, C(C***H***_3_)_3_); 1.63–1.75(4H, CNH(CH_2_)_3_C***H***_2_CH_2_); 1.77–1.88 (4H, CNHCH_2_C***H***_2_(CH_2_)_3_); 2.89–3.04 (4H, CNHC***H***_2_(CH_2_)_4_); 3.53–3.64 (4H, CNH(CH_2_)_4_C***H***_2_NH_2_) ppm. ^13^C NMR (150 MHz, CD_3_OD): δ 24.6 (CNH(CH_2_)_2_***C***H_2_(CH_2_)_2_); 31.7 (C(***C***H_3_)_3_); 32.4 (CNH(CH_2_)_3_***C***H_2_CH_2_); 32.7 (CNHCH_2_***C***H_2_(CH_2_)_3_); 46.1 (CNH***C***H_2_(CH_2_)_4_NH_2_); 50.7 (CNH(CH_2_)_4_***C***H_2_NH_2_); 53.5 (***C***(CH_3_)_3_); 191.3 ((NH)(NH)***C***Au) ppm. MS (*m*/*z*): calcd for [C_20_H_46_Au_2_N_6_]^2+^, 382.1552; Found [C_20_H_46_Au_2_N_6_]^2+^, 382.1551. Anal. Calcd for C_20_H_46_Au_2_N_6_Cl_2_·2H_2_O: C, 27.56; H, 5.78; N, 9.64. Found: C, 27.30; H, 5.40; N, 9.23.

#### Complex **5**

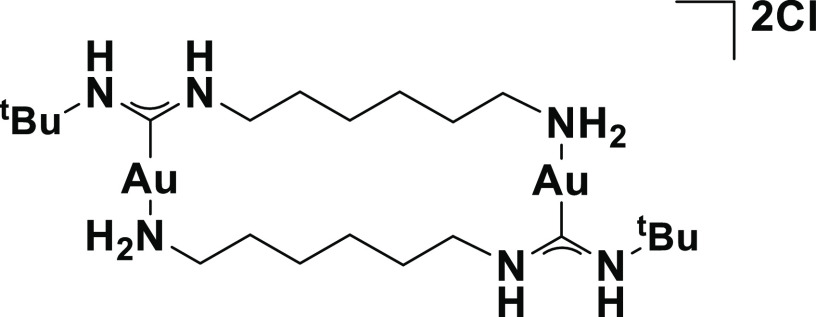



Yield: 102 mg (75%). ^1^H NMR (600 MHz, CD_3_OD): δ 1.38–1.52 (8H, CNH(CH_2_)_2_(C***H***_2_)_2_(CH_2_)_2_NH_2_); 1.52–1.61 (18H, C(C***H***_3_)_3_); 1.66 (4H, CNHCH_2_C***H***_2_(CH_2_)_4_NH_2_); 1.78 (4H, CNH(CH_2_)_4_(C***H***_**2**_)CH_2_NH_2_); 2.90–3.03 (4H, CNHC***H***_2_(CH_2_)_5_); 3.56–3.68 (4H, CNH(CH_2_)_5_C***H***_2_NH_2_) ppm. ^13^C NMR (150 MHz, CD_3_OD): δ 26.2 (CNH(CH_2_)_2_(***C***H_2_)_2_(CH_2_)_2_NH_2_); 30.4 (C(***C***H_3_)_3_); 31.2 (CNHCH_2_***C***H_2_(CH_2_)_4_NH_2_); 32.0 (CNH(CH_2_)_4_***C***H_2_CH_2_NH_2_); 45.0 (CNH***C***H_2_(CH_2_)_5_); 49.9 (CNH(CH_2_)_5_***C***H_2_NH_2_); 53.4 (***C***(CH_3_)_3_); 191.1 ((NH)(NH)***C***Au) ppm. MS (*m*/*z*): calcd for [C_22_H_50_Au_2_N_6_]^2+^, 396.1709; found [C_22_H_50_Au_2_N_6_]^2+^, 396.1700. Anal. Calcd for C_22_H_50_Au_2_N_6_Cl_2_·H_2_O: C, 29.97; H, 5.95; N, 9.53. Found: C, 29.76; H, 5.56; N, 9.35.

### General Procedure for the Synthesis of Bis-(N^*t*^Bu)(NC_*n*_H_2*n*_)CAuCl

To a solution containing the *t*-butyl isocyanide gold chloride complex (49 mg, 0.156 mmol, 1 equiv) and a minimum amount of DCM (1 mL), 0.45 equiv of a secondary diamine was added to the reaction mixture. The reaction mixture was stirred for 6 h at 22 °C. The product showed slight solubility in DCM, so the mixture was concentrated under reduced pressure, followed by the addition of 1 mL of cold Et_2_O, and kept stirring for an additional 15 min. *n*-Pentane was then added in a small amount (200 μL), and the resulting precipitate was filtered and washed three times with a Et_2_O *n*-pentane 70:30 (v/v) mixture. The obtained white precipitate was dried under vacuum overnight and analyzed without further purification.

#### Complex **6**

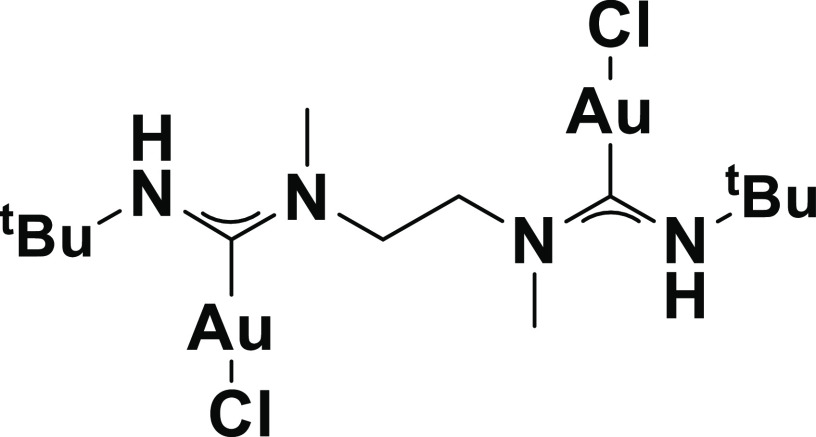



Yield: 41 mg (72%). ^1^H NMR (600 MHz, CDCl_3_): δ 1.62 (18H, C(C***H***_3_)_3_); 3.15 (6H, CN(C***H***_3_)(CH_2_)_2_(C***H***_3_)NC); 4.19 (4H, CN(CH_3_)(C***H***_2_)_2_(CH_3_)NC); 5.93 (2H, (CH_3_)_3_CN***H***C) ppm. ^13^C NMR (150 MHz, CDCl_3_): δ 31.8 (C(***C***H_3_)_3_); 35.6 (CN(***C***H_3_) (CH_2_)_2_(***C***H_3_)NC); 54.7 (***C***(CH_3_)_3_); 59.6 (CN(CH_3_)(***C***H_2_)_2_(CH_3_)NC); 191.4 ((N)(NH)***C***Au) ppm. MS (*m*/*z*): calcd for: [C_14_H_30_Au_2_ClN_4_]^+^, 683.1485; found [C_14_H_30_Au_2_ClN_4_]^+^, 683.1481. Anal. Calcd for C_14_H_30_Au_2_Cl_2_N_4_: C, 23.38; H, 4.20; N, 7.79. Found: C, 23.24; H, 3.96; N, 7.64.

#### Complex **7**

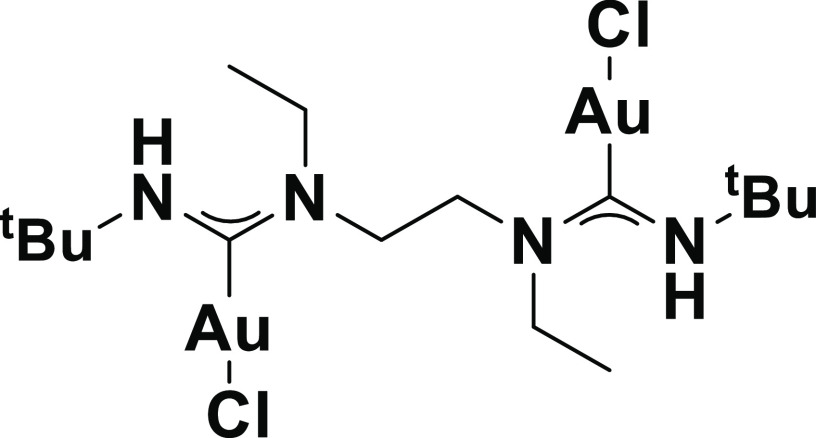



Yield: 26 mg (44%). ^1^H NMR (600 MHz, CDCl_3_): δ 1.19 (6H, CN(CH_2_C***H***_3_)(CH_2_)_2_(CH_2_C***H***_3_)NC); 1.50–1.69 (18H, C(C***H***_3_)_3_); 3.53 (4H, CN(C***H***_2_CH_3_)(CH_2_)_2_(C***H***_2_CH_3_)NC); 4.12 (4H, CN(CH_2_CH_3_)(C***H***_2_)_2_(CH_2_CH_3_)NC); 6.03 (2H, (CH_3_)_3_CN***H***C) ppm. ^13^C NMR (150 MHz, CDCl_3_): δ 11.1 (CN(CH_2_***C***H_3_)(CH_2_)_2_(CH_2_***C***H_3_)NC); 32.1 (C(***C***H_3_)_3_); 42.1 (CN(***C***H_2_CH_3_)(CH_2_)_2_(***C***H_2_CH_3_)NC); 54.8 (***C***(CH_3_)_3_); 57.2 (CN(CH_3_)(***C***H_2_)_2_(CH_3_)NC); 192.0 ((N)(NH)***C***Au) ppm. MS (*m*/*z*): calcd for [C_16_H_34_Au_2_ClN_4_]^+^, 711.1798; found [C_16_H_34_Au_2_ClN_4_]^+^, 711.1800. Anal. Calcd for C_16_H_34_Au_2_Cl_2_N_4_: C, 25.72; H, 4.59; N, 7.50. Found: C, 26.01; H, 4.68; N, 7.46.

#### Complex **8**

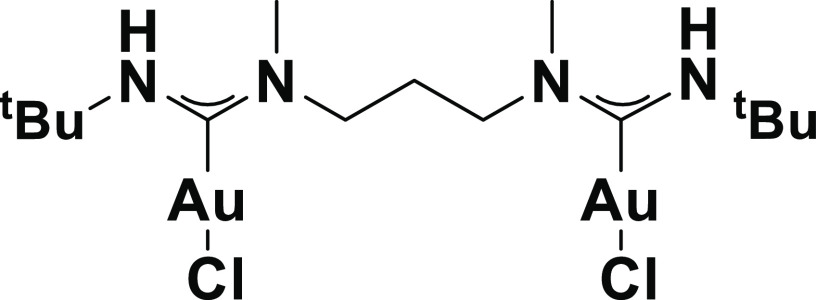



Yield: 34 mg (59%). ^1^H NMR (600 MHz, CDCl_3_): δ 1.65 (18H, C(C***H***_3_)_3_); 1.95 (2H, CN(CH_3_)(CH_2_)(C***H***_2_)(CH_2_)(CH_3_)NC); 3.04 (6H, CN(C***H***_3_)(CH_2_)_3_(C***H***_3_)NC); 4.15 (4H, CN(CH_3_)(C***H***_2_)(CH_2_)(C***H***_2_)(CH_3_)NC); 5.98 (2H, (CH_3_)_3_CN***H***C) ppm. ^13^C NMR (150 MHz, CDCl_3_): δ 27.8 (CN(CH_3_)(CH_2_)(***C***H_2_)(CH_2_)(CH_3_)NC); 31.6 (C(***C***H_3_)_3_); 34.0 (CN(***C***H_3_)(CH_2_)_2_(***C***H_3_)NC); 54.6 (***C***(CH_3_)_3_); 59.2 (CN(CH_3_)(***C***H_2_)(CH_2_)(***C***H_2_)(CH_3_)NC); 190.7 ((N)(NH)***C***Au) ppm. MS (*m*/*z*): calcd for [C_15_H_32_Au_2_ClN_4_]^+^, 697.1641; found [C_15_H_32_Au_2_ClN_4_]^+^, 697.1640. Anal. Calcd for C_15_H_32_Au_2_Cl_2_N_4_: C, 24.57; H, 4.40; N, 7.64. Found: C, 24.26; H, 4.13; N, 7.42.

#### Complex **9**

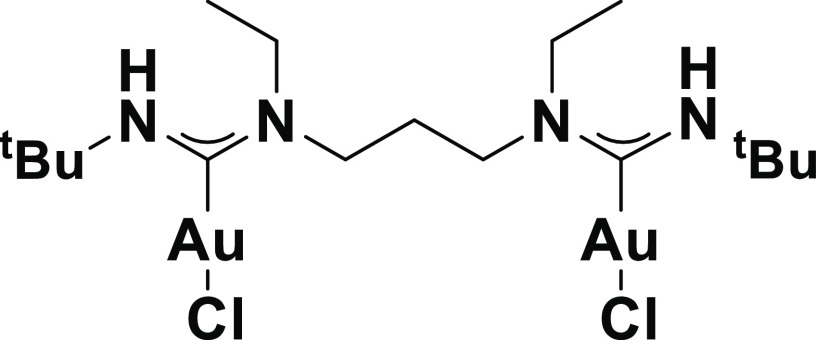



Yield: 53 mg (88%). ^1^H NMR (600 MHz, CDCl_3_): δ 1.17 (6H, CN(CH_2_C***H***_3_)(CH_2_)_3_(CH_2_C***H***_3_)NC); 1.64 (18H, C(C***H***_3_)_3_); 1.98 (2H, CN(CH_2_CH_3_)(CH_2_)(C***H***_2_)(CH_2_)(CH_2_CH_3_)NC); 3.44 (4H, CN(C***H***_2_CH_3_)(CH_2_)_3_(C***H***_2_CH_3_)NC); 4.11 (4H, CN(CH_2_CH_3_)(C***H***_2_)(CH_2_)(C***H***_2_)(CH_2_CH_3_)NC); 6.05 (2H, (CH_3_)_3_CN***H***C) ppm. ^13^C NMR (150 MHz, CDCl_3_): δ 11.4 (CN(CH_2_C***H***_3_)(CH_2_)_3_(CH_2_C***H***_3_)NC); 28.8 (CN(CH_2_CH_3_)(CH_2_)(***C***H_2_)(CH_2_)(CH_2_CH_3_)NC); 31.6 (C(***C***H_3_)_3_); 41.0 (CN(***C***H_2_CH_3_)(CH_2_)_3_(***C***H_2_CH_3_)NC); 54.5 (***C***(CH_3_)_3_); 56.9 (CN(CH_2_CH_3_)(***C***H_2_)(CH_2_)(***C***H_2_)(CH_2_CH_3_)NC); 189.9 ((N)(NH)***C***Au) ppm. MS (*m*/*z*): calcd for [C_17_H_36_Au_2_ClN_4_]^+^, 725.1954; found [C_17_H_36_Au_2_ClN_4_]^+^, 725.1957. Anal. Calcd for C_17_H_36_Au_2_Cl_2_N_4_: C, 26.82; H, 4.77; N, 7.36. Found: C, 26.83; H, 4.56; N, 7.19.

### Computational Details

All calculations were executed using ORCA 5.0.1.^33−35^ Geometry optimizations and vibrational frequencies were calculated using the BP86 functional^36,37^ with an atom pairwise dispersion correction (D3),^38,39^ together with the def2-TZVP basis set^40^ and effective core potentials for gold.^41^ These calculations were performed using RI density fitting approximations with the def2/J Coulomb fitting basis set.^42^ A rotor approximation was applied to low-frequency vibrational modes as described by Grimme.^43^ Solvation by DCM was modeled using the conductor-like polarizable continuum model.^44,45^ Single-point electronic energies were recalculated with the B2PLYP-D3^46^ functional and the def2-TZVP basis set, together with the def2-TZVP/C auxiliary basis^47^ set for RI approximation for the perturbation step.

## Supporting Information Available

Additional characterization data of all compounds (^1^H and ^13^C NMR spectra and MS-spectra), supplementary X-ray crystallography data, and additional computational details and data (PDF)
*xyz* coordinates (XYZ)

## Supplementary Material

ic2c00509_si_001.pdf

ic2c00509_si_002.xyz

## Accession Codes

CCDC 2150198–2150202 contain the supplementary crystallographic data for this paper. These data can be obtained free of charge via www.ccdc.cam.ac.uk/data_request/cif, or by emailing data_request@ccdc.cam.ac.uk, or by contacting The Cambridge Crystallographic Data Centre, 12 Union Road, Cambridge CB2 1EZ, UK; fax: +44 1223 336033.
