# Supplementary material for: Reactivity of Diamines in Acyclic Diamino Carbene Gold Complexes
Source: Inorg Chem. 2022 May 4;61(19):7448–58. doi: 10.1021/acs.inorgchem.2c00509 (PMC9115764; doi:10.1021/acs.inorgchem.2c00509)
Supplement: Supplementary file 1 — ic2c00509_si_001.pdf [file ic2c00509_si_001.pdf]

Reactivity of Diamines in Acyclic Diamino Carbene Gold Complexes

Guilherme M. D. M. Rúbio[a]‡, Tristan T. Y. Tan[b]‡, Alexander Prado-Roller[c], Jia Min Chin[c], Michael R. Reithofer[a]**

a Institute of Inorganic Chemistry, Faculty of Chemistry, University of Vienna, Vienna A-1090, Austria,

email: michael.reithofer@univie.ac.at

b Institute of Materials Research and Engineering, A*STAR (Agency for Science, Technology and Research), Singapore 138634, Singapore

c Institute of Inorganic Chemistry - Functional Materials, Faculty of Chemistry, University of Vienna, Vienna A-1090, Austria

email: jiamin.chin@univie.ac.at

‡These authors contributed equally

Table of Contents

NMR Spectra of complex 1 3
NMR Spectra of complex 2 6
NMR Spectra of complex 3 7
NMR Spectra of complex 4 8
NMR Spectra of complex 5 10
NMR Spectra of complex 6 11
NMR Spectra of complex 7 12
NMR Spectra of complex 8 13
NMR Spectra of complex 9 14
Mass spectra of complex 1.......... 15
Mass spectra of complex 2.......... 17
Mass spectra of complex 3.......... 19
Mass spectra of complex 4.......... 21
Mass spectra of complex 5.......... 22
Mass spectra of complex 6.......... 23
Mass spectra of complex 7.......... 24
Mass spectra of complex 8.......... 25
Mass spectra of complex 9.......... 26
Crystallographic data for complex 1.......... 27
Crystallographic data for complex 3.......... 27
Crystallographic data for complex 6.......... 28
Crystallographic data for complex 7.......... 29
Crystallographic data for complex 9.......... 30
DFT calculations.......... 32

NMR Spectra of complex 1

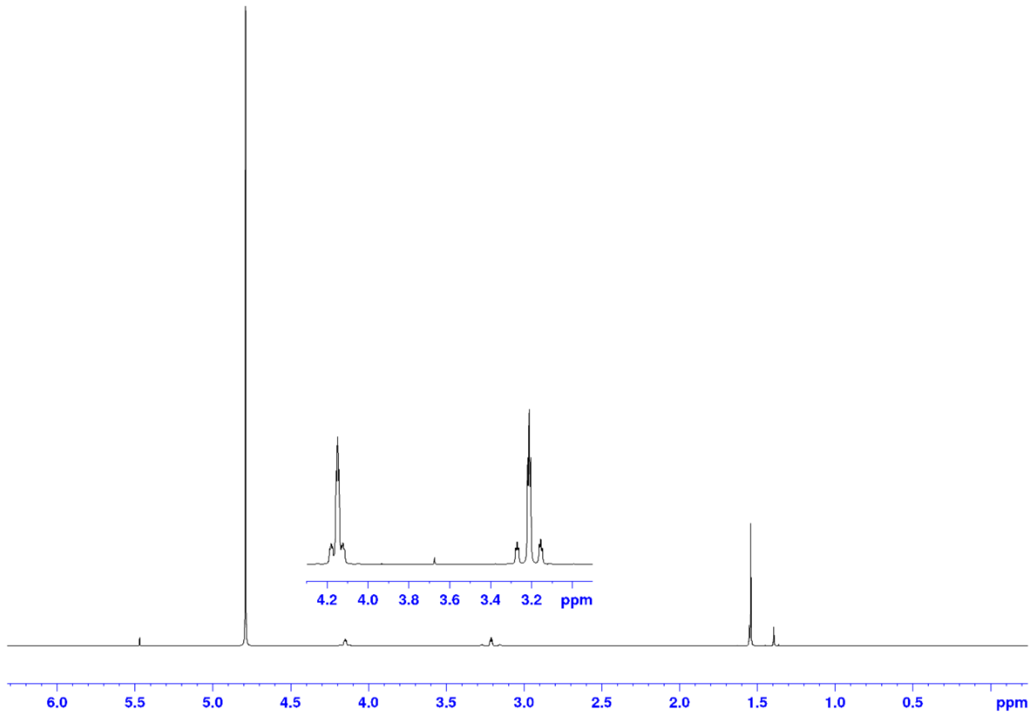

Figure S 1. ^{1}H-NMR spectrum of complex **1**.

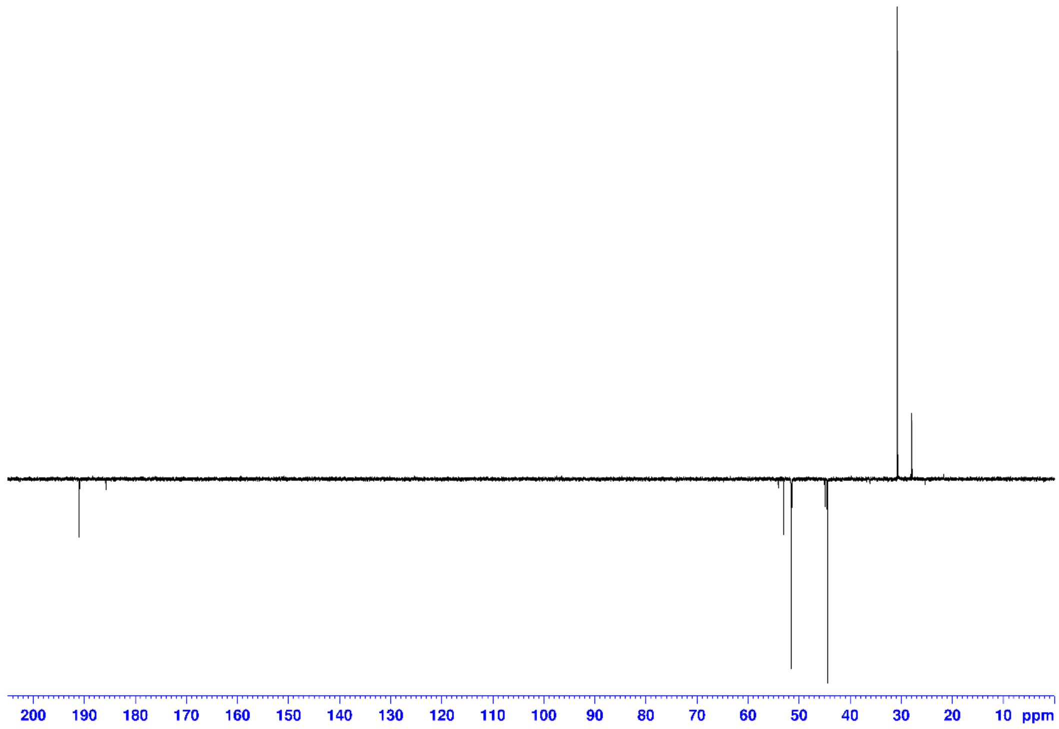

Figure S 2. ^{13}C-NMR spectrum of complex **1**

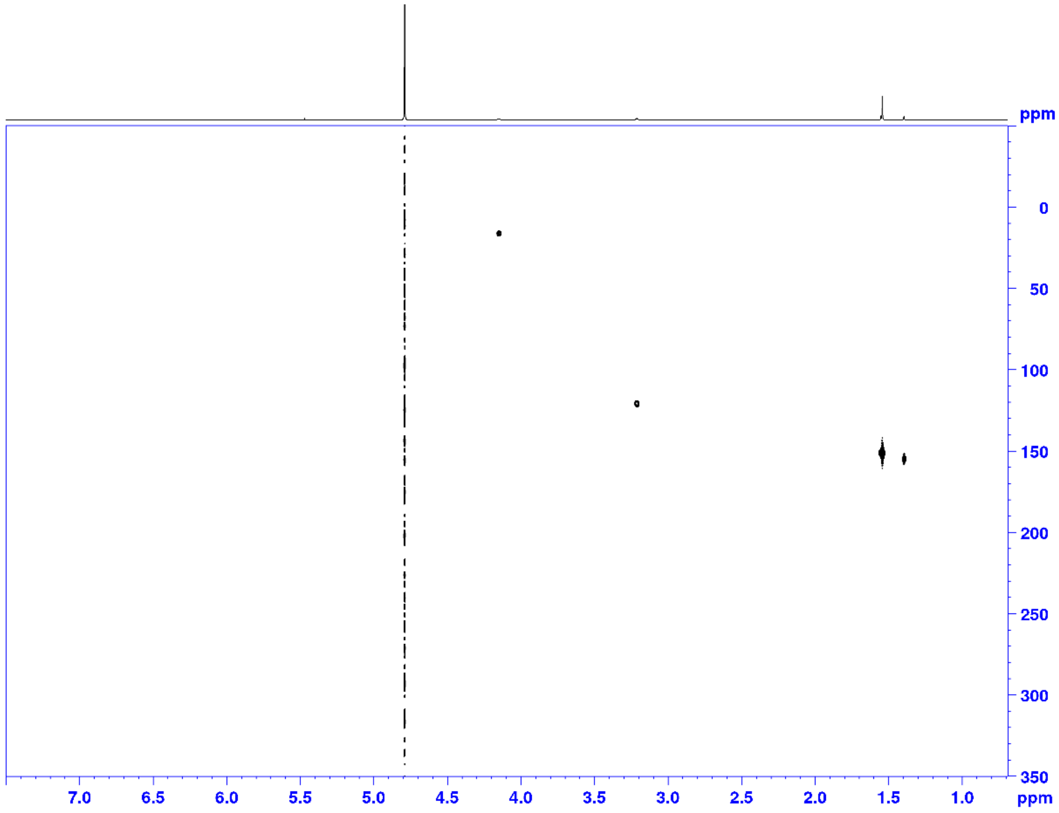

Figure S 3. ^{1}H-^{15}N HMBC spectrum of complex **1**

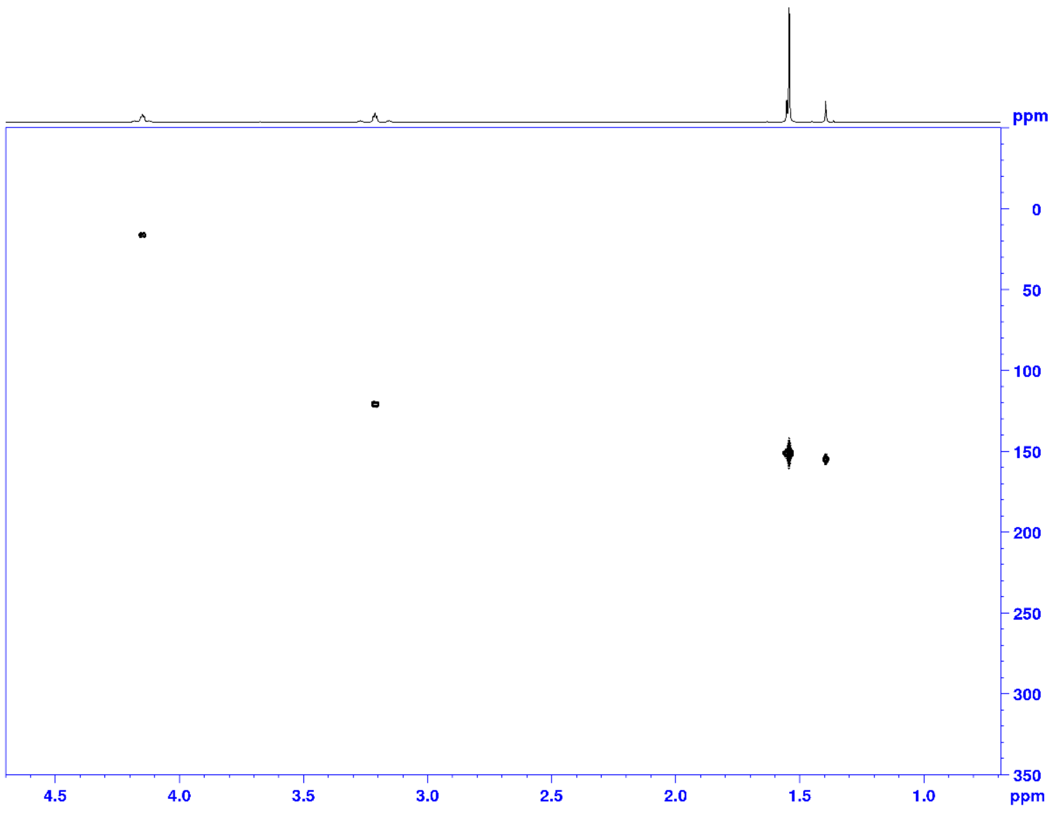

Figure S 4. Zoom of ^{1}H-^{15}N HMBC spectrum of complex **1**

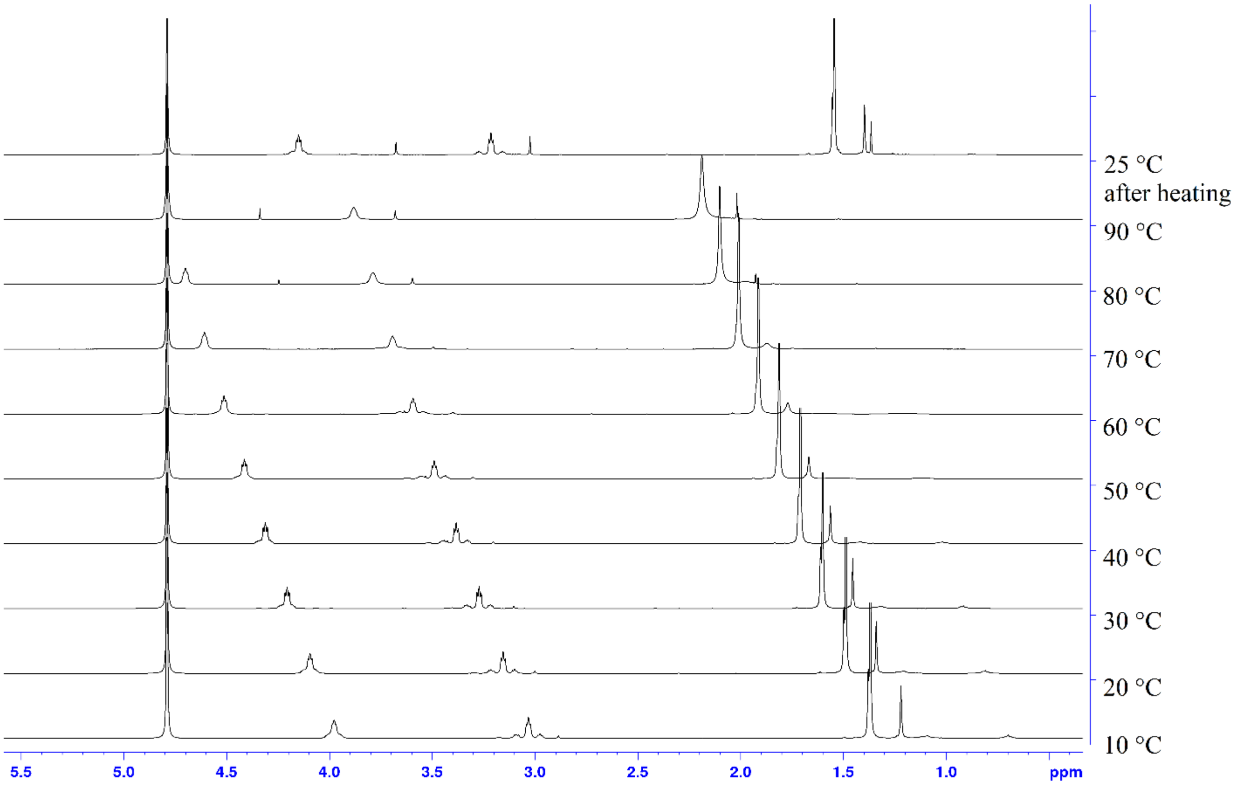

Figure S 5. Variable temperature ^{1}H-NMR spectra of complex **1** from T = 10 °C to 90 °C and subsequent cooling down to 25 °C

NMR Spectra of complex 2

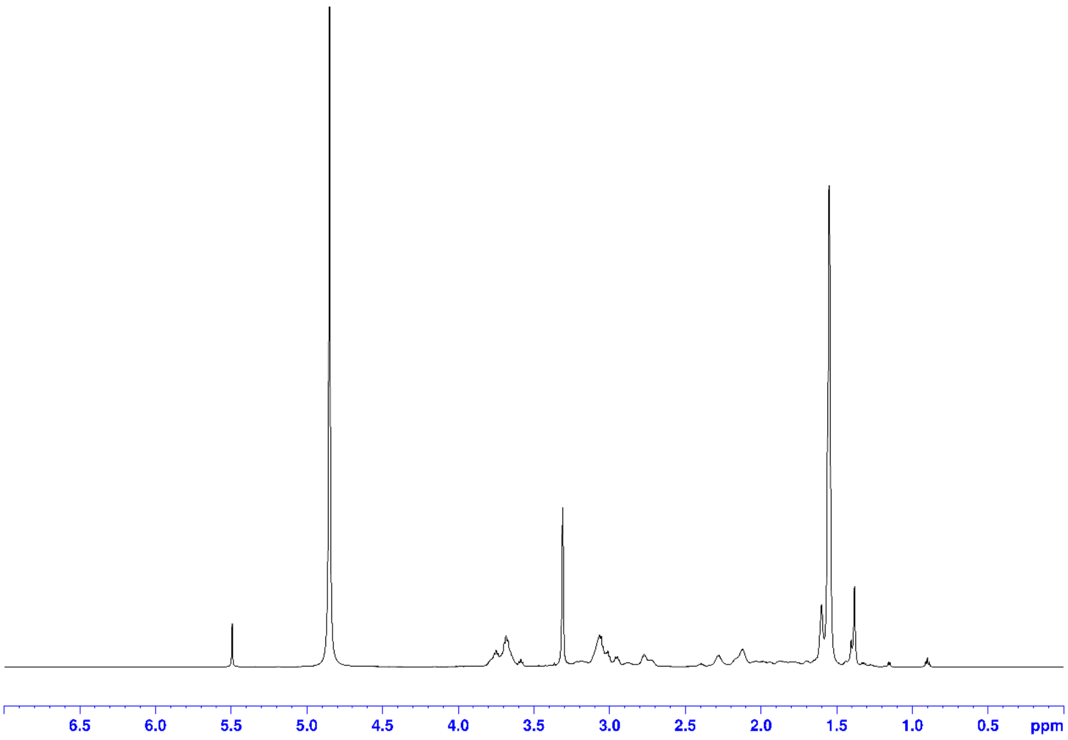

Figure S 6. ^{1}H-NMR spectrum of complex **2**

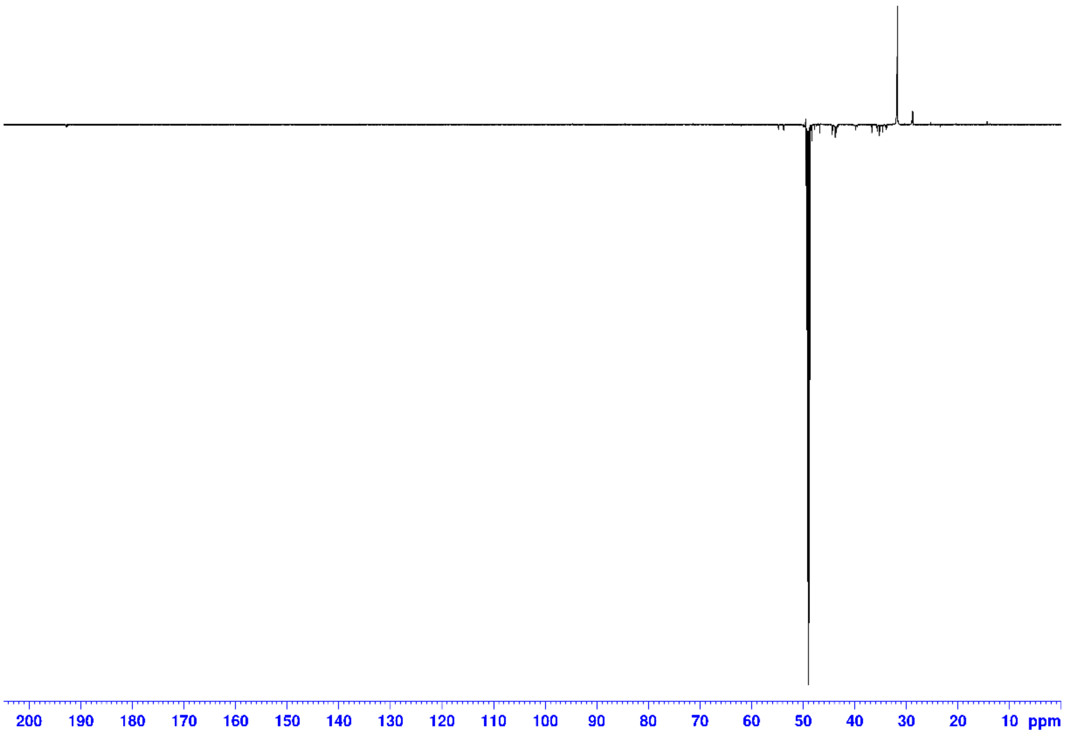

Figure S 7. ^{13}C-NMR spectrum of complex **2**

NMR Spectra of complex 3

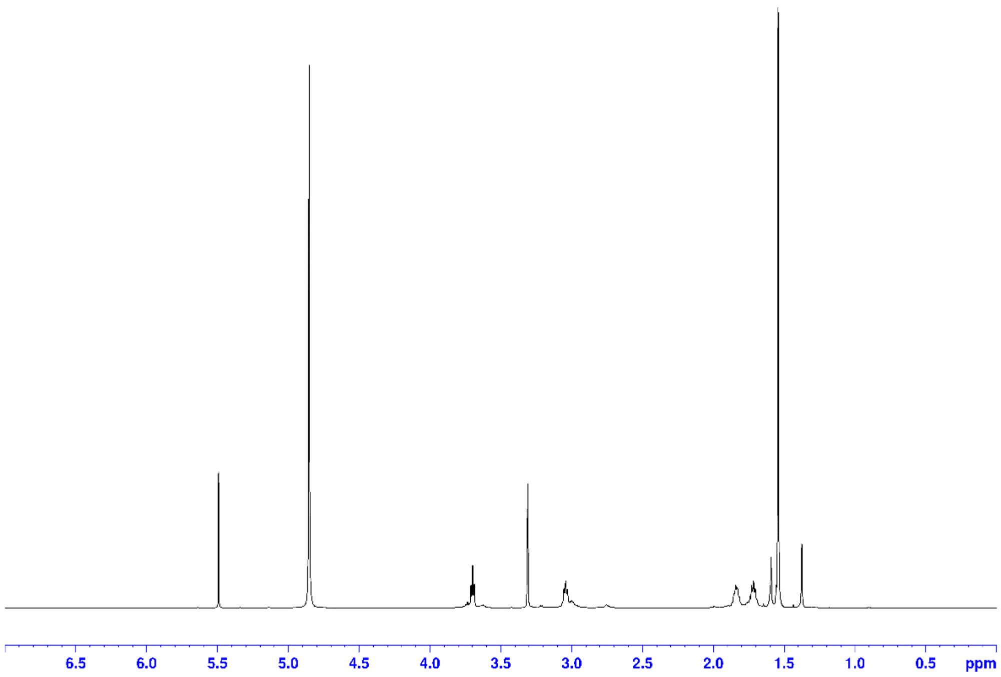

Figure S 8. ^{1}H-NMR spectrum of complex **3**

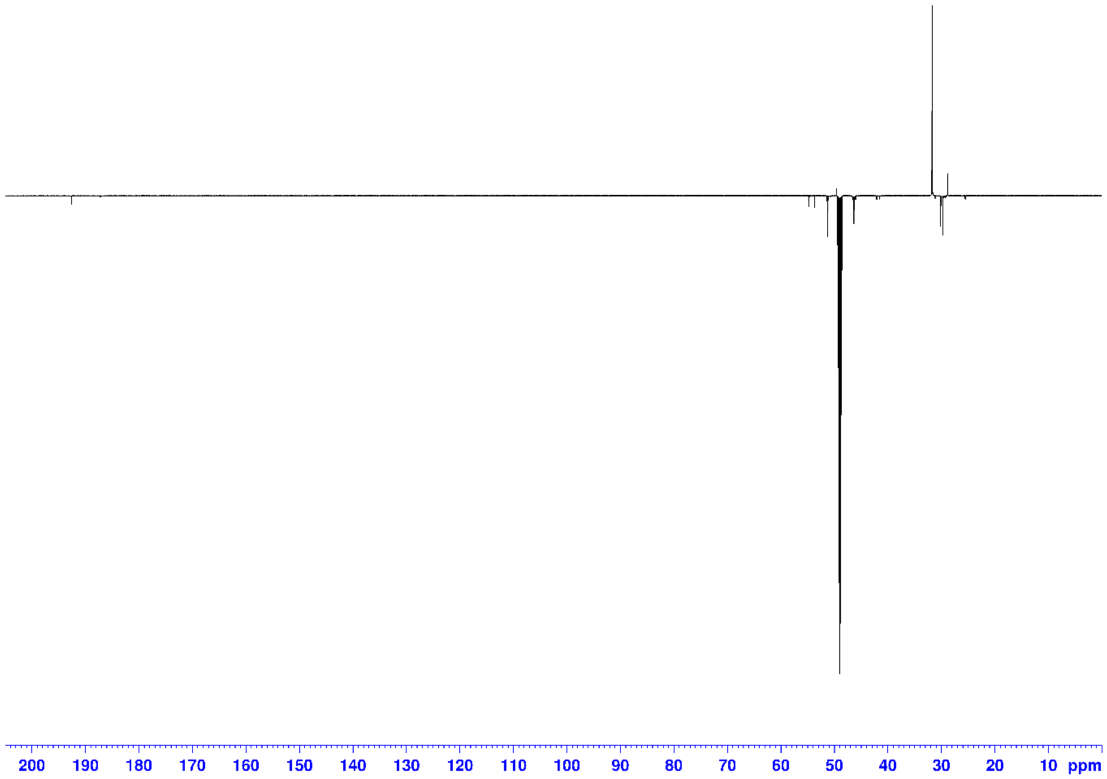

Figure S 9. ^{13}C-NMR spectrum of complex **3**

NMR Spectra of complex 4

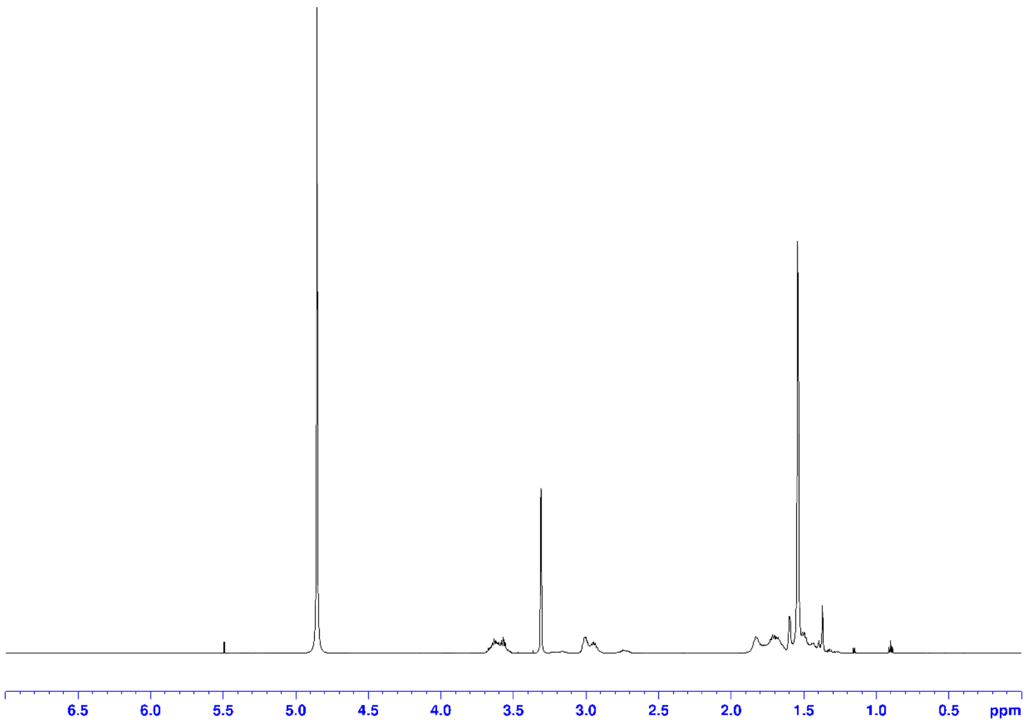

Figure S 10. ^{1}H-NMR spectrum of complex **4**

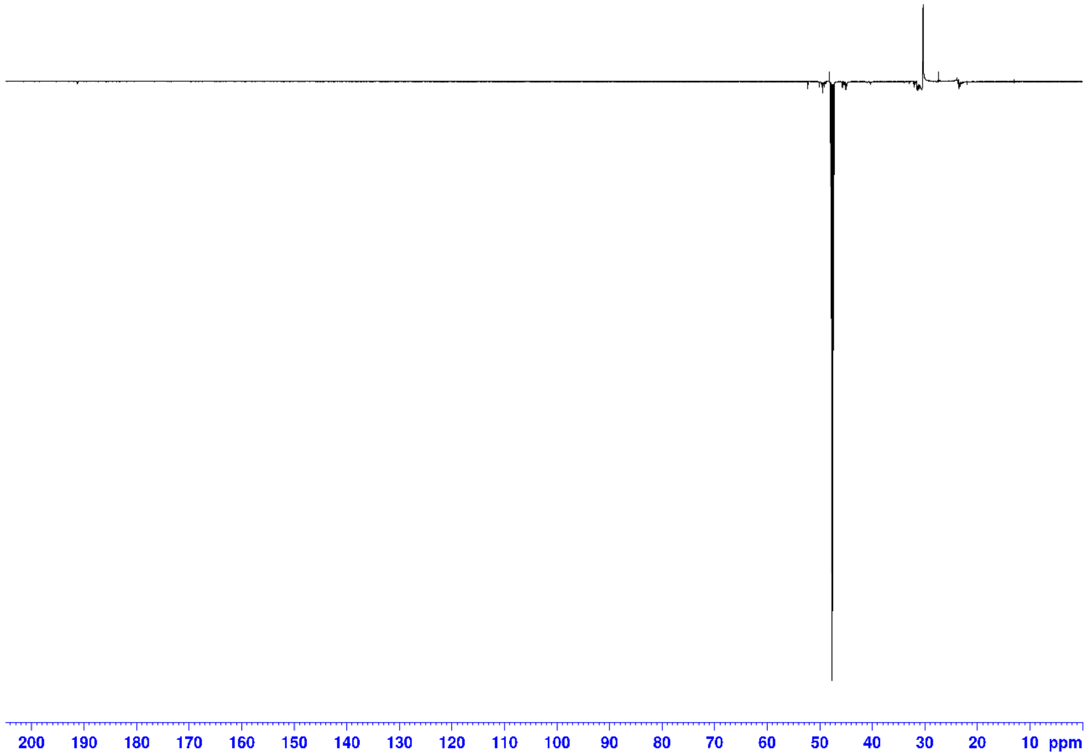

Figure S 11. ^{13}C-NMR spectrum of complex **4**

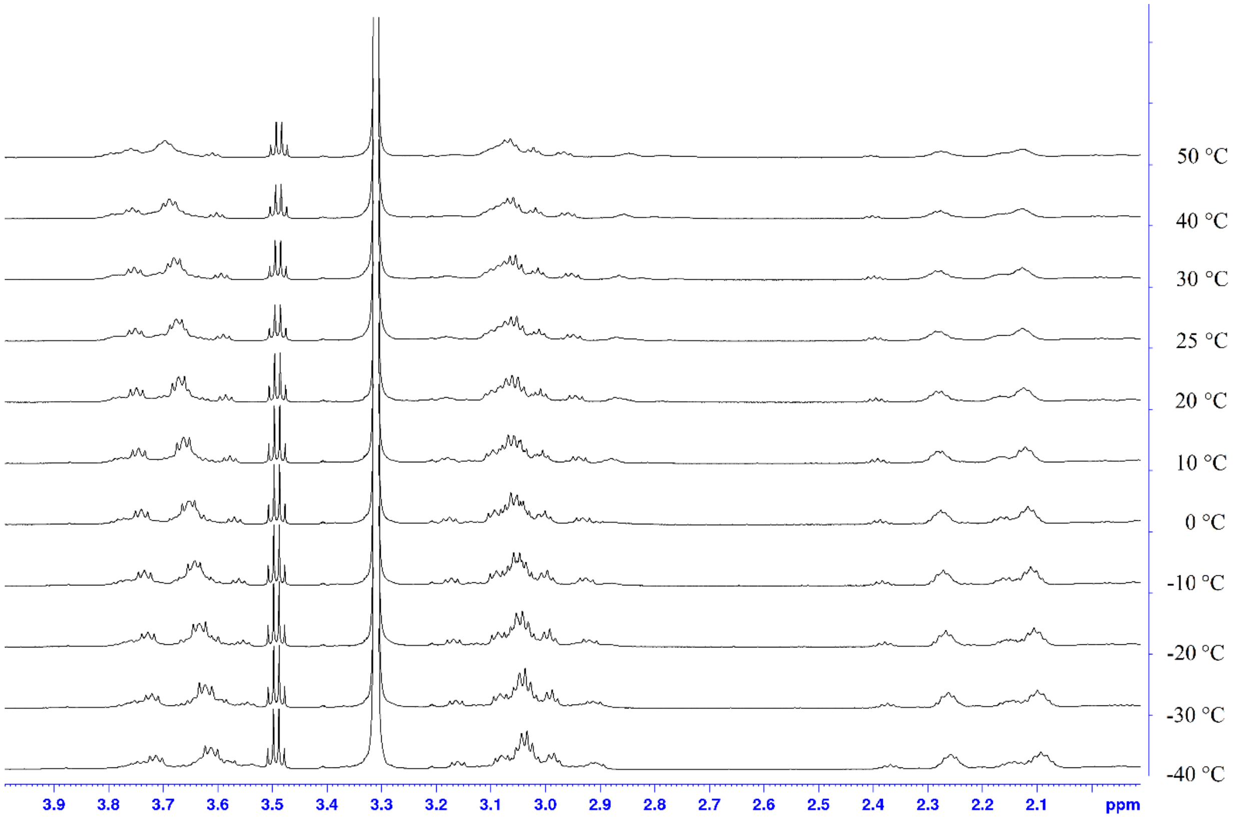

Figure S 12. ^{1}H-NMR VT experiment spectrum of complex **4** in the range of T = -40 °C to 50 °C

NMR Spectra of complex 5

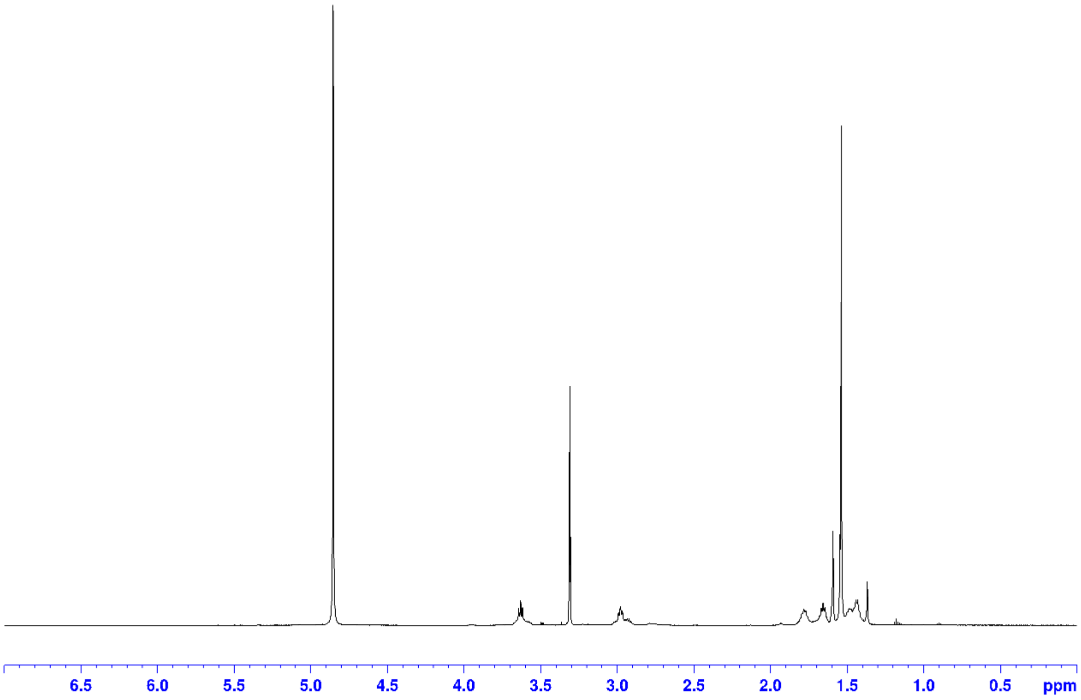

Figure S 13. ^{1}H-NMR spectrum of complex **5**

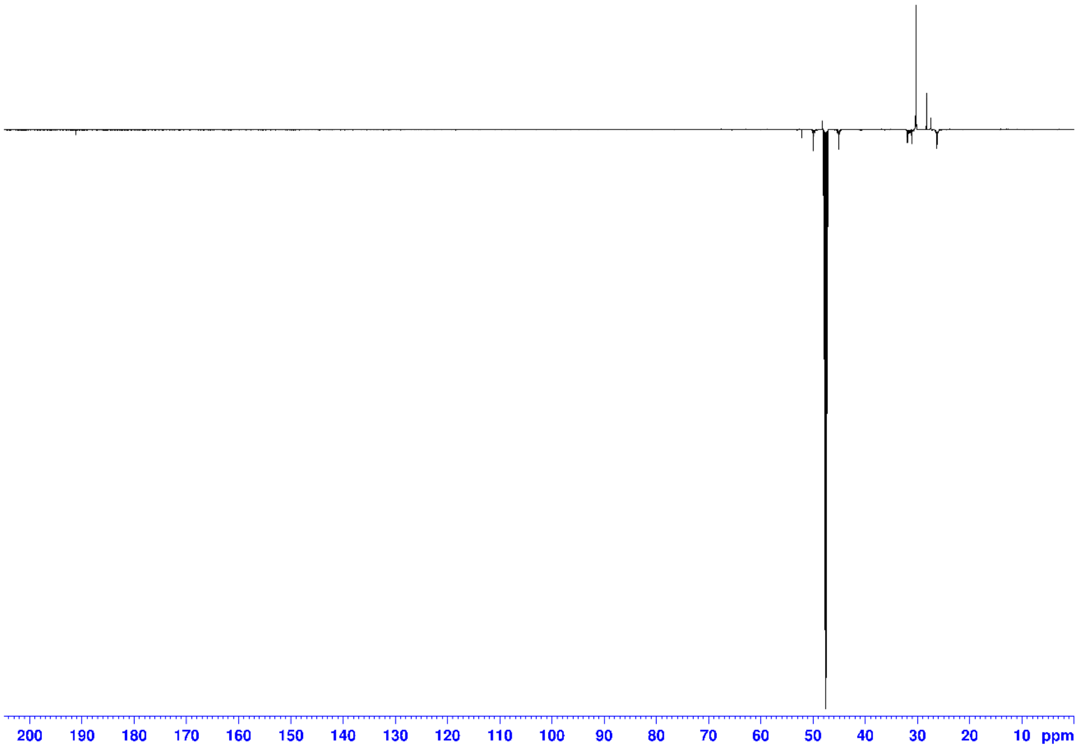

Figure S 14. ^{13}C-NMR spectrum of complex **5**

NMR Spectra of complex 6

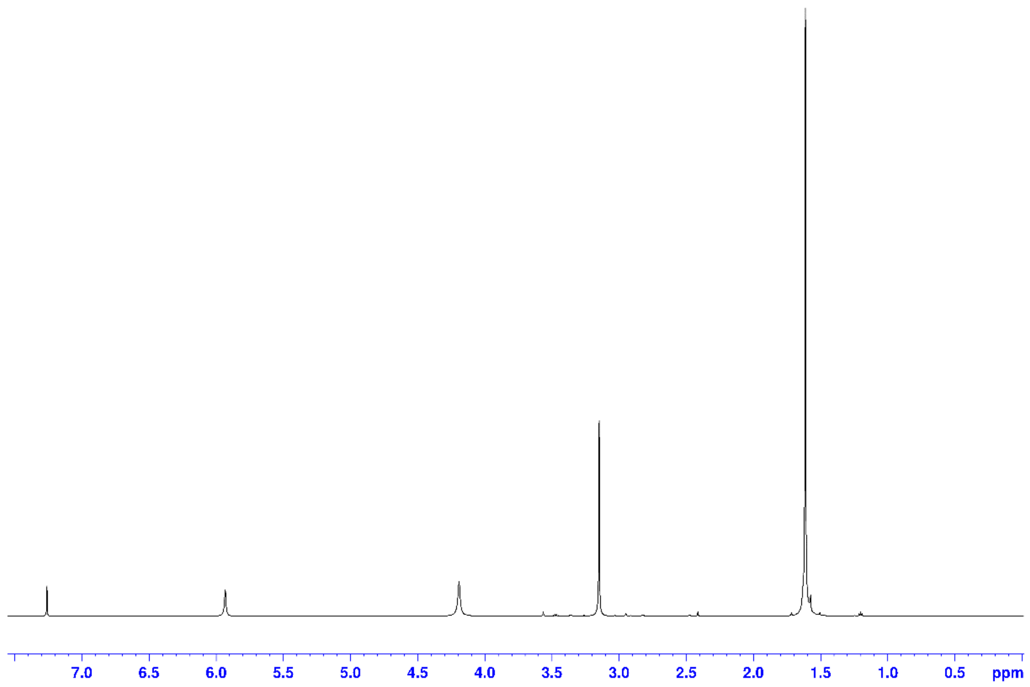

Figure S 15. ^{1}H-NMR spectrum of complex **6**

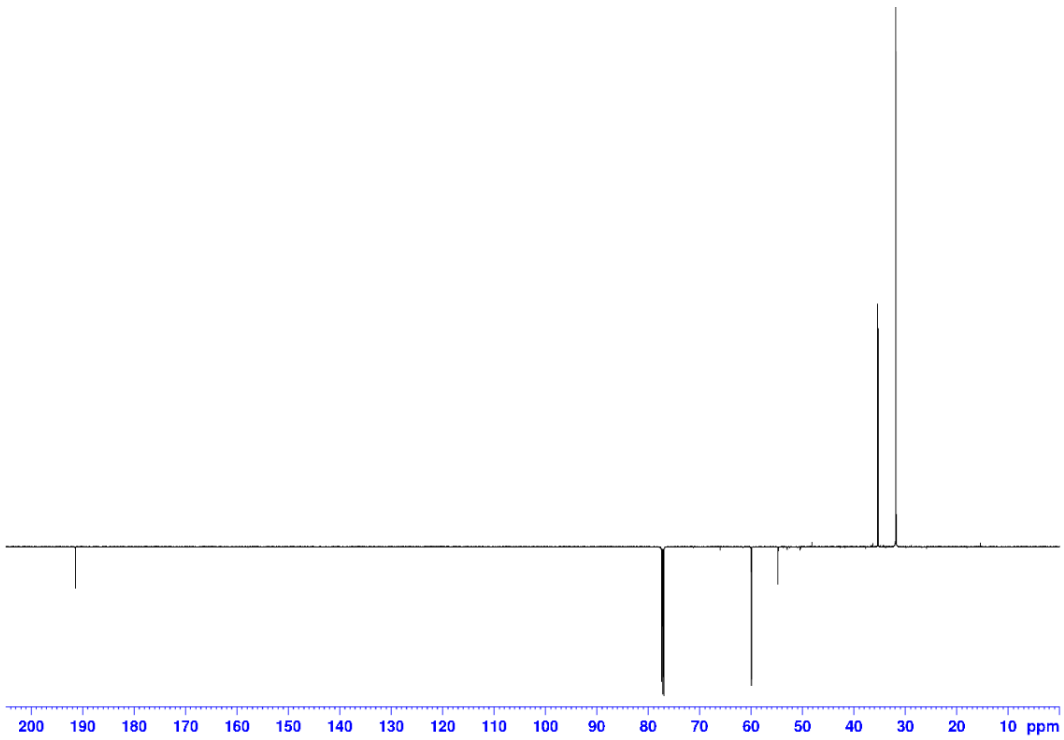

Figure S 16. ^{13}C-NMR spectrum of complex **6**

NMR Spectra of complex 7

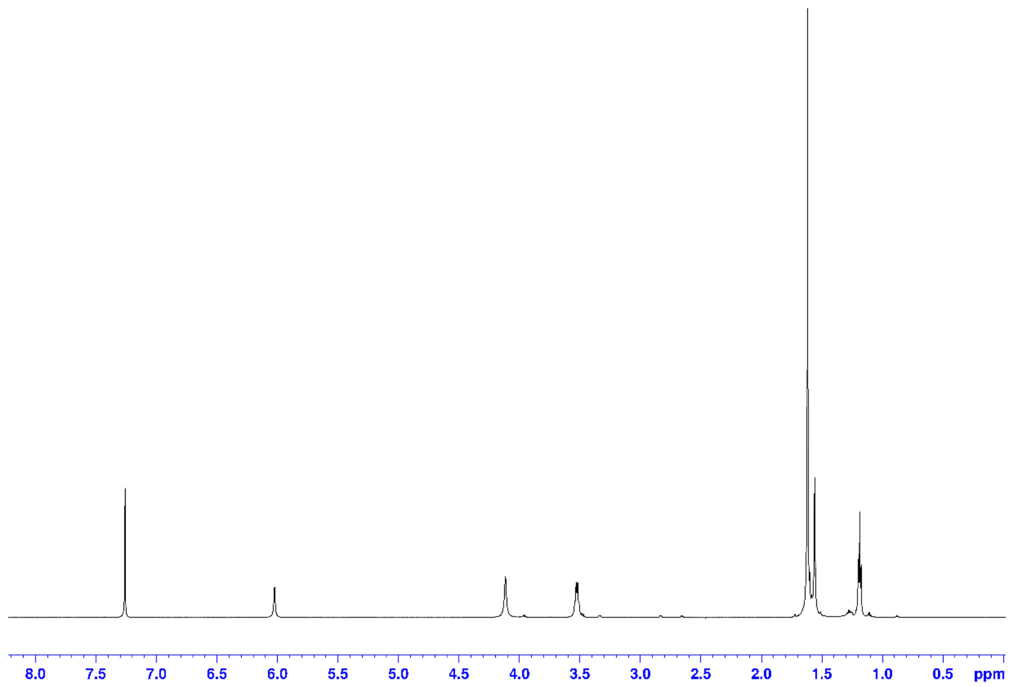

Figure S 17. ^{1}H-NMR spectrum of complex **7**

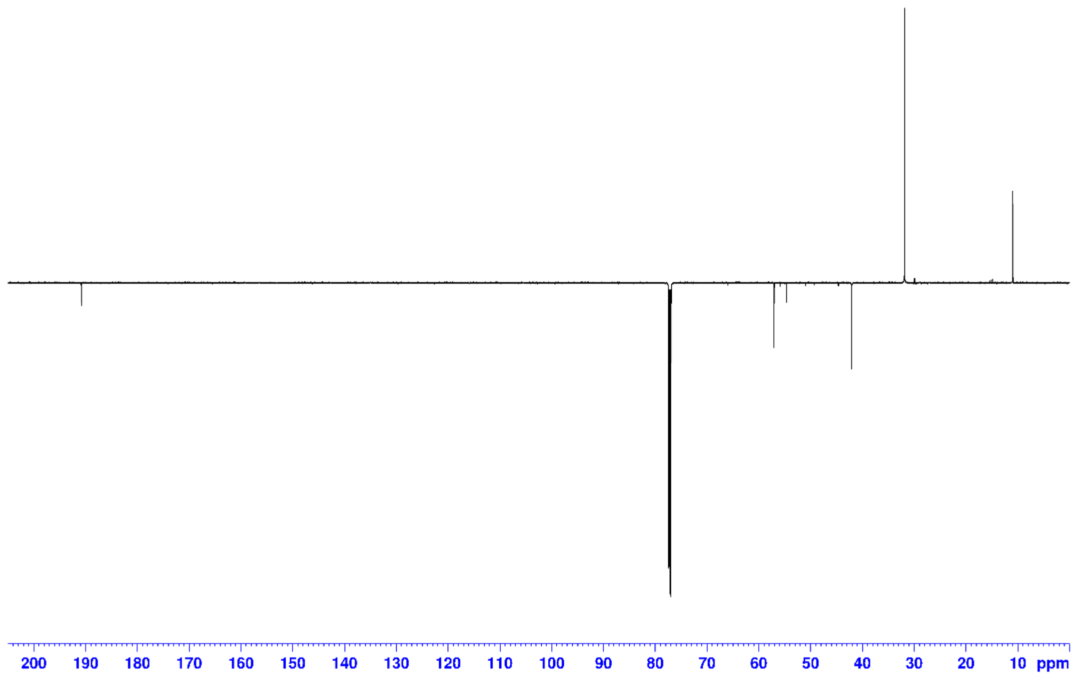

Figure S 18. ^{13}C-NMR spectrum of complex **7**

NMR Spectra of complex 8

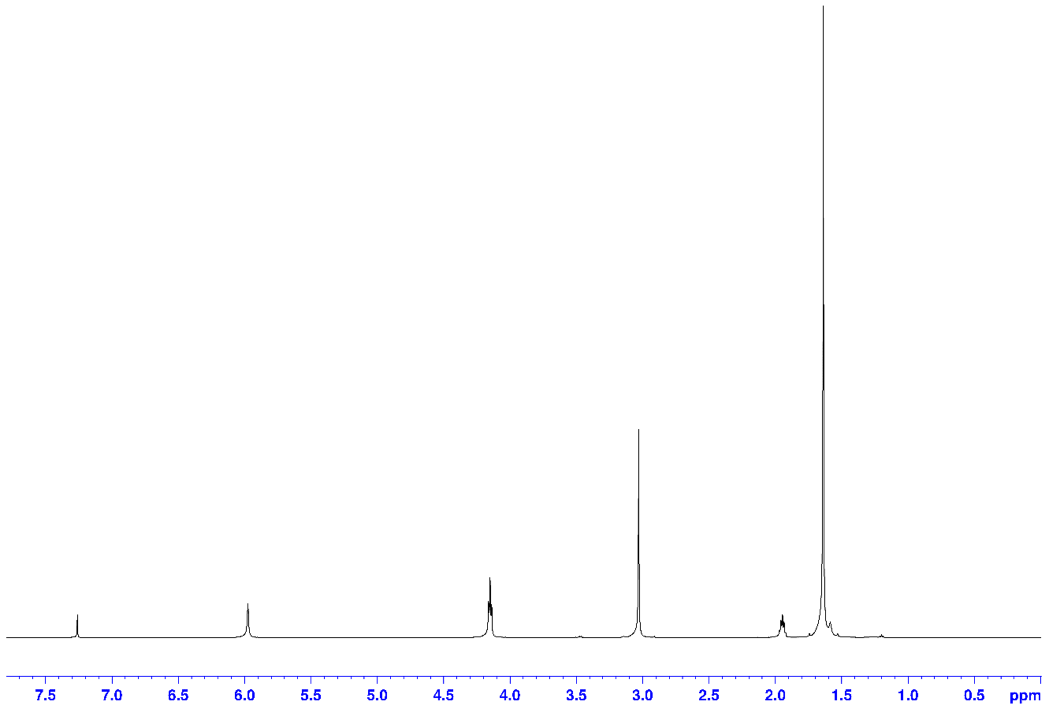

Figure S 19. ^{1}H-NMR spectrum of complex **8**

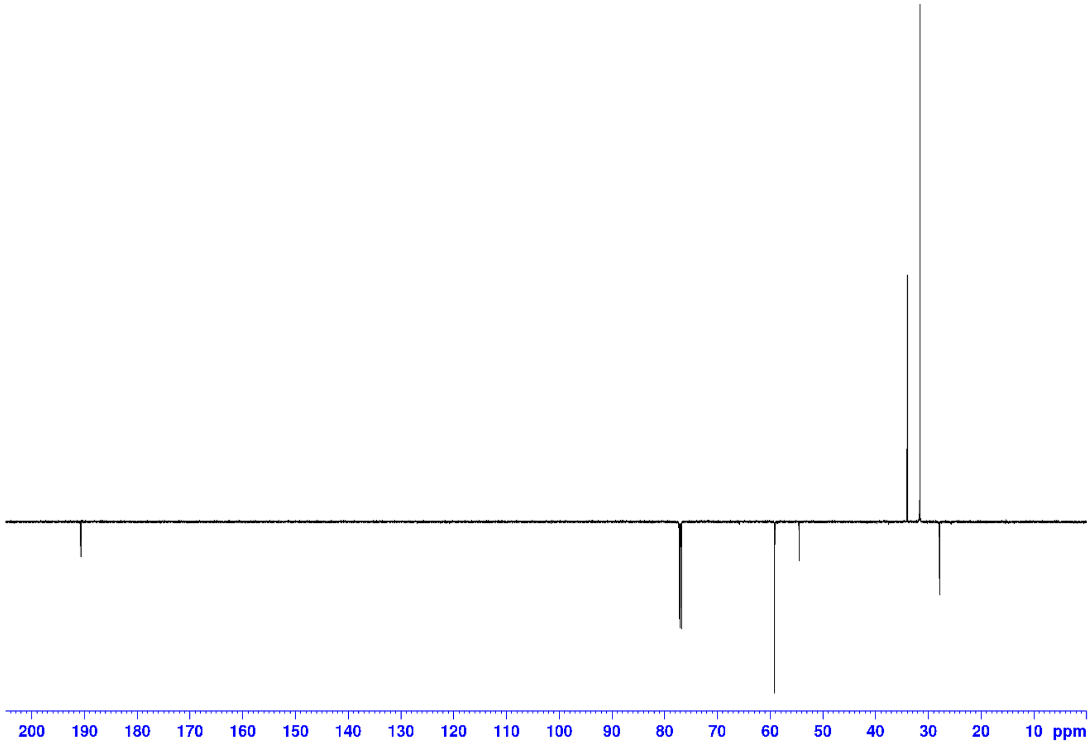

Figure S 20. ^{13}C-NMR spectrum of complex **8**

NMR Spectra of complex 9

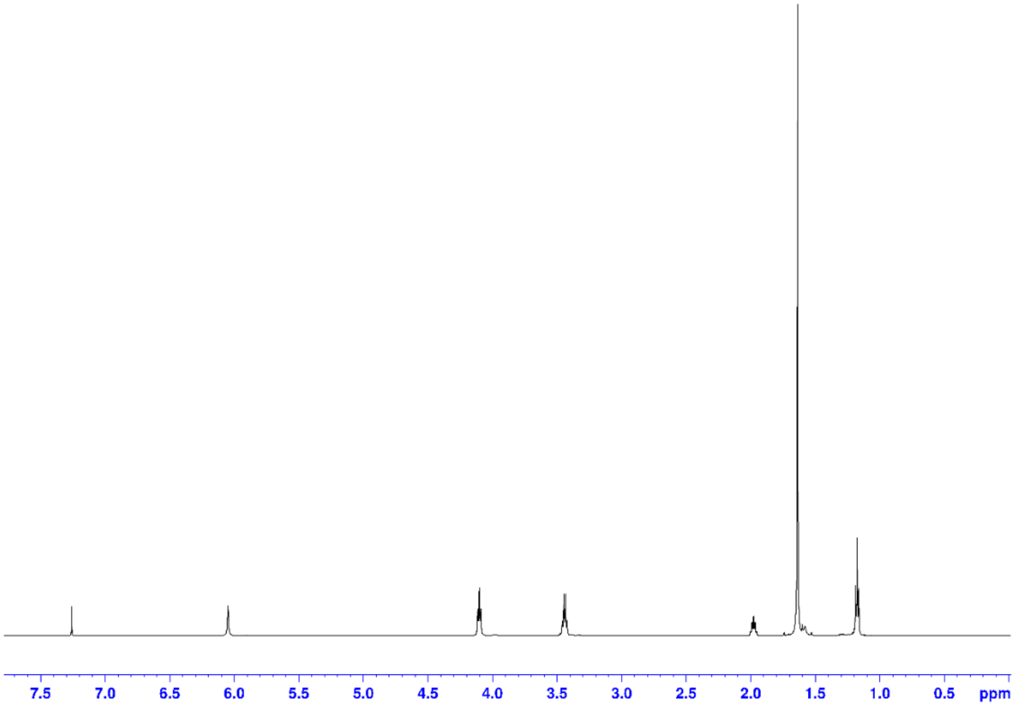

Figure S 21. ^{1}H-NMR spectrum of complex **9**

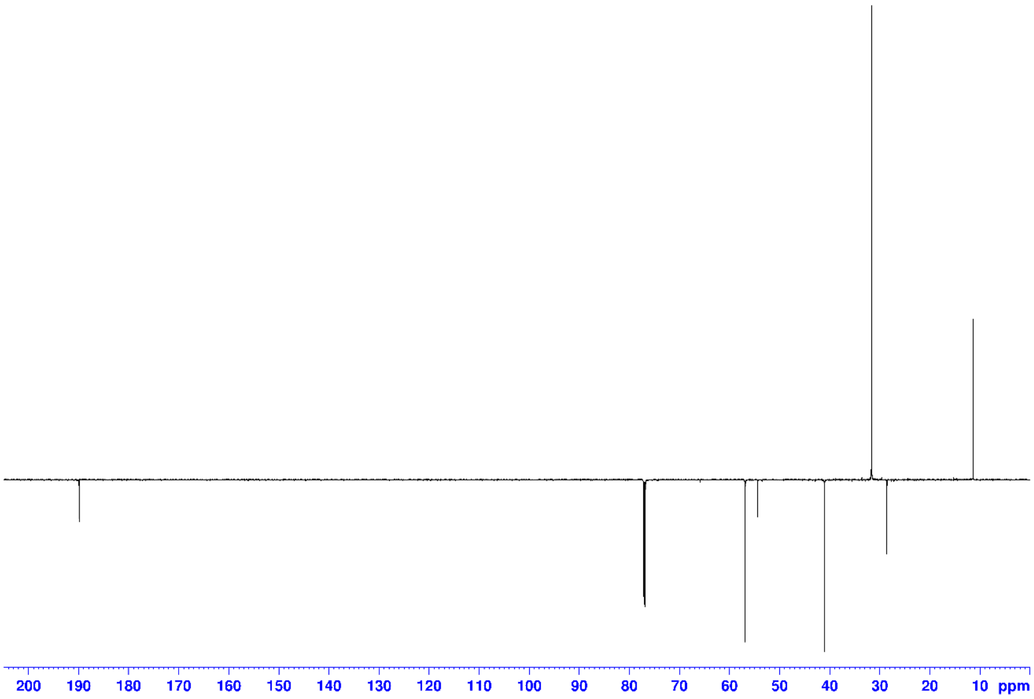

Figure S 22. ^{13}C-NMR spectrum of complex **9**

Mass spectra of complex 1

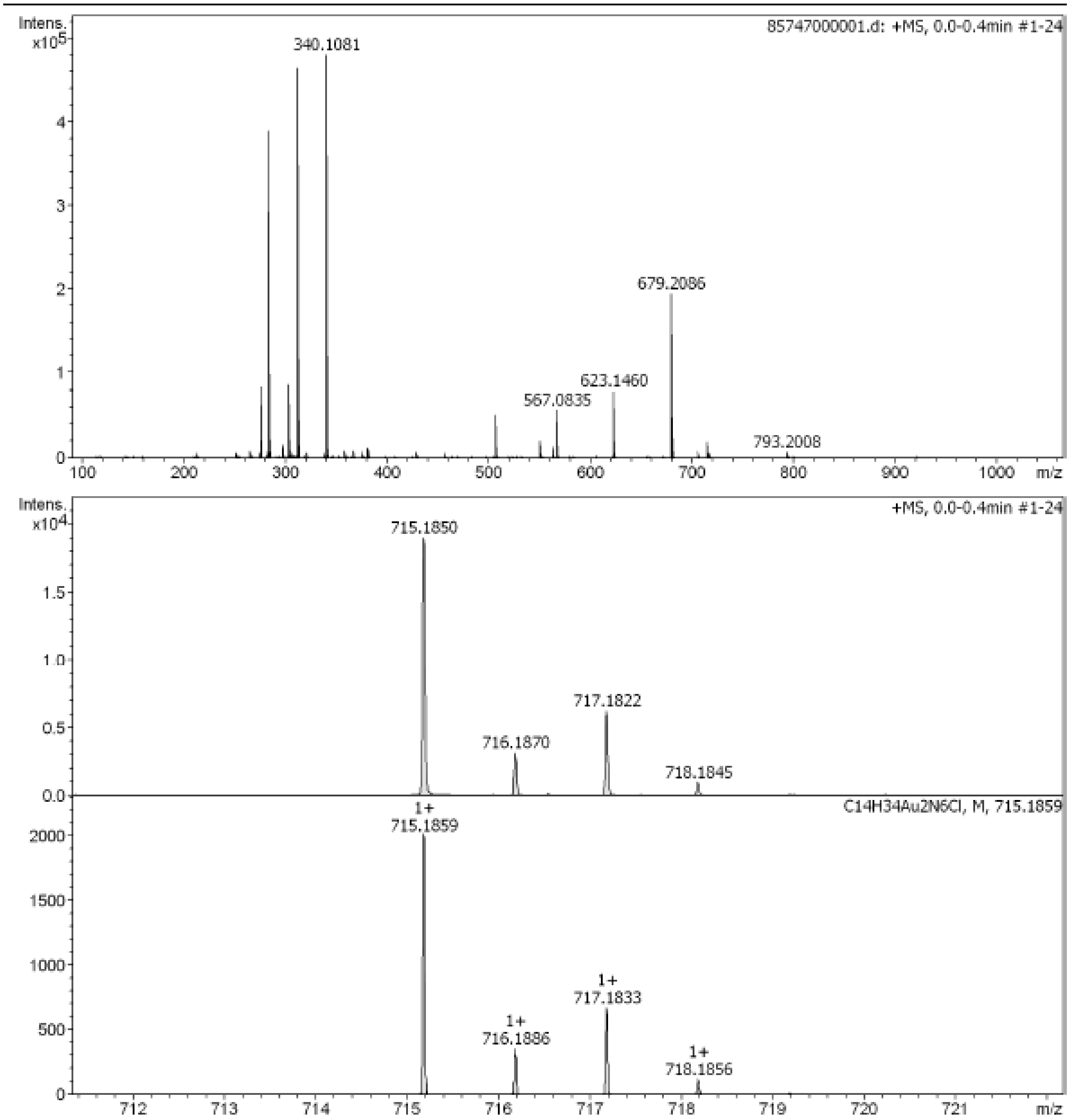

Figure S 23. Mass spectra of complex **1** [M-Cl$^-$]$^+$

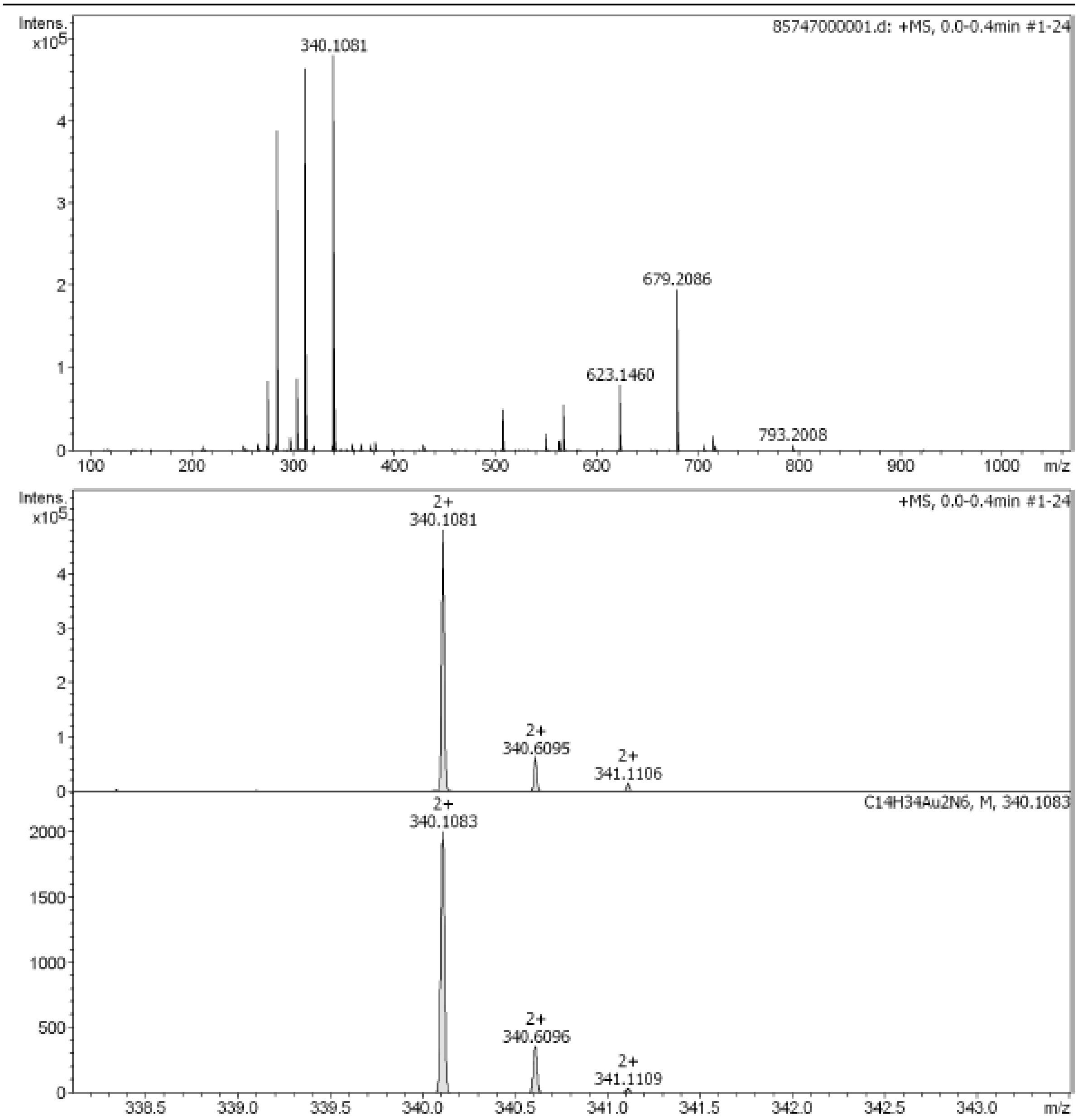

Figure S 24. Mass spectra of complex **1** $[M\text{-}2Cl^{-}]^{2+}$

Mass spectra of complex 2

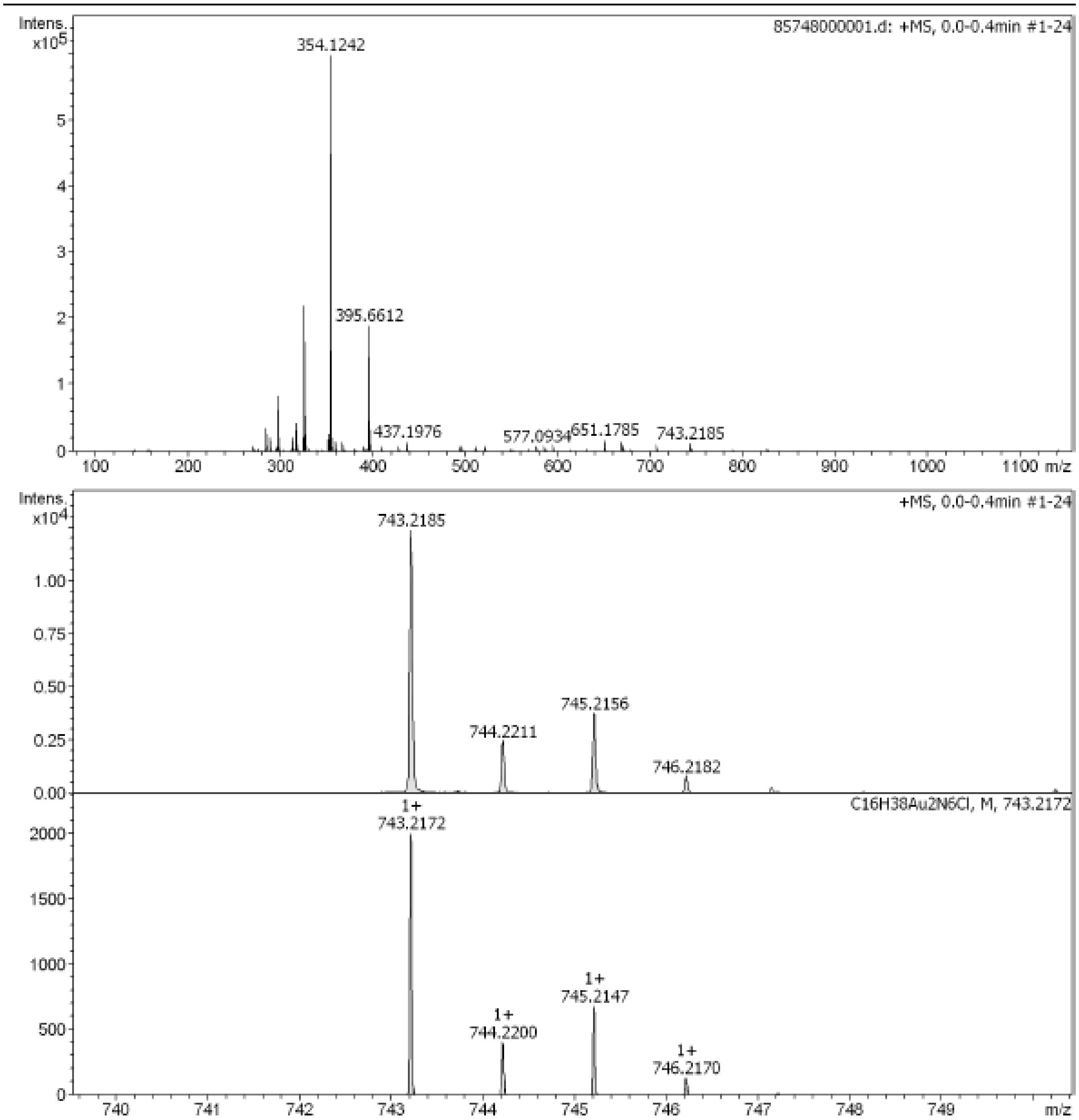

Figure S 25. Mass spectra of complex **2** $[M-Cl^{-}]^{+}$

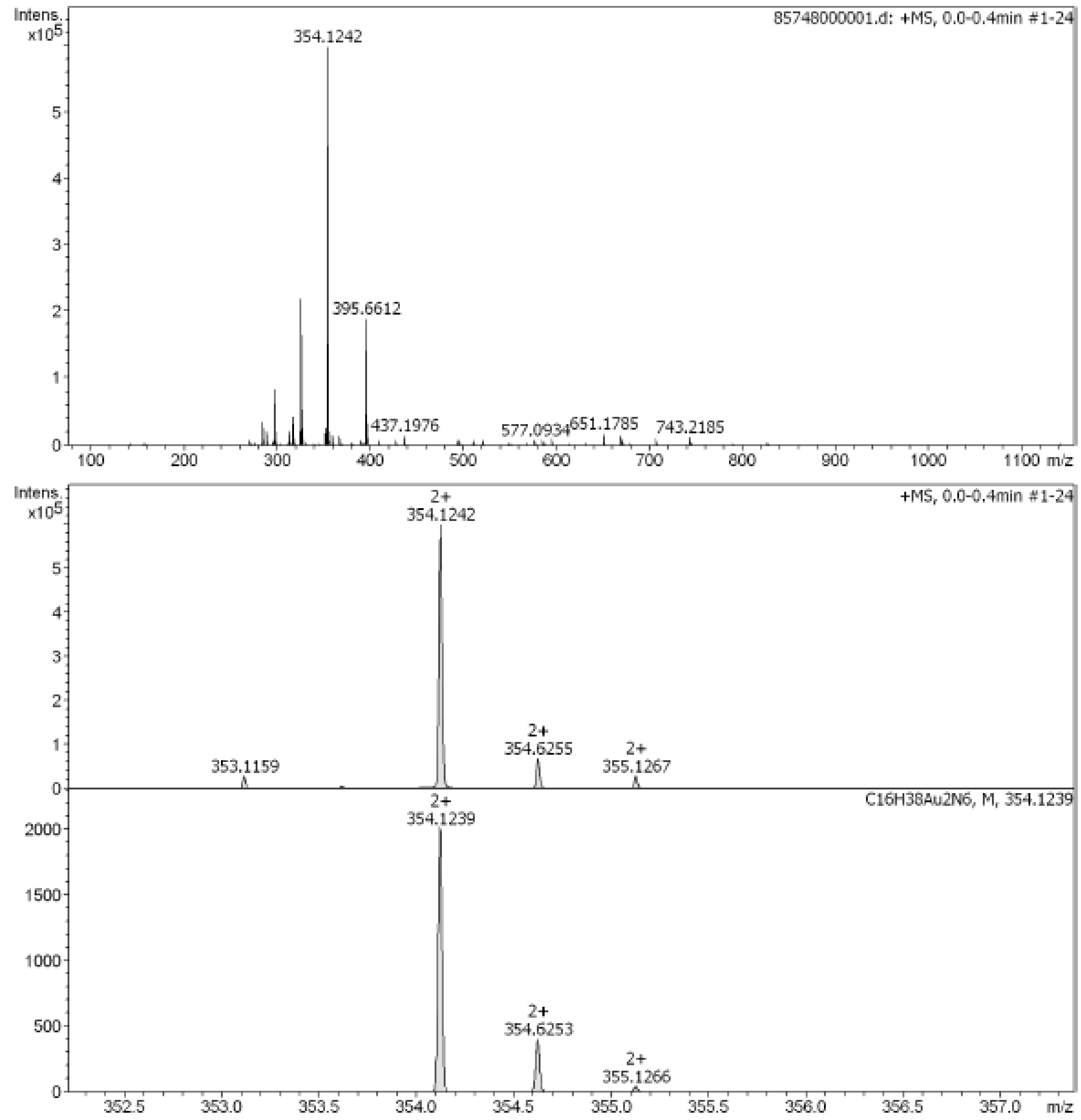

Figure S 26. Mass spectra of complex **2** [M-2Cl$^-$]$^{2+}$

Mass spectra of complex 3

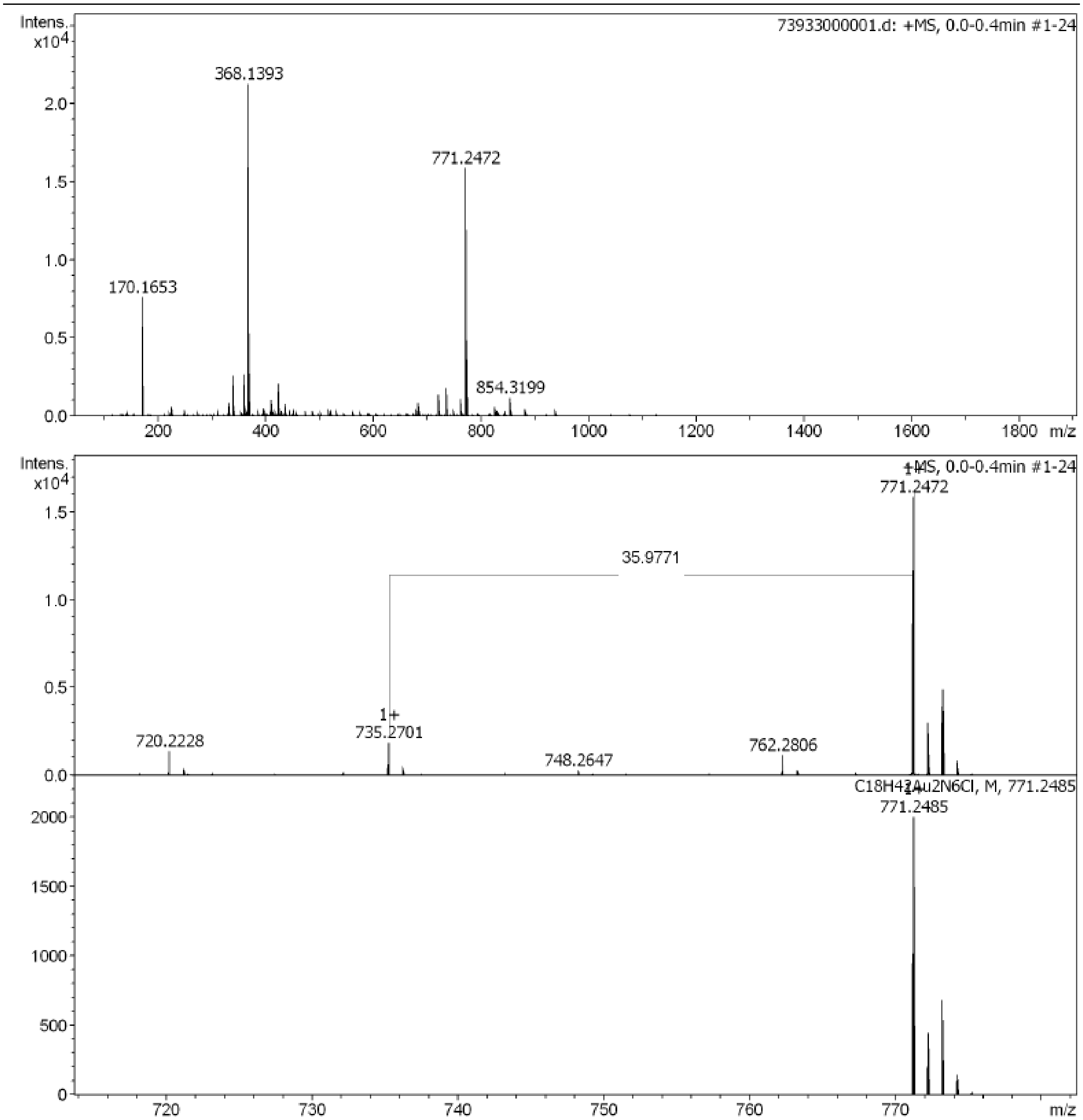

Figure S 27. Mass spectra of complex **3** $[M-Cl^{-}]^{+}$

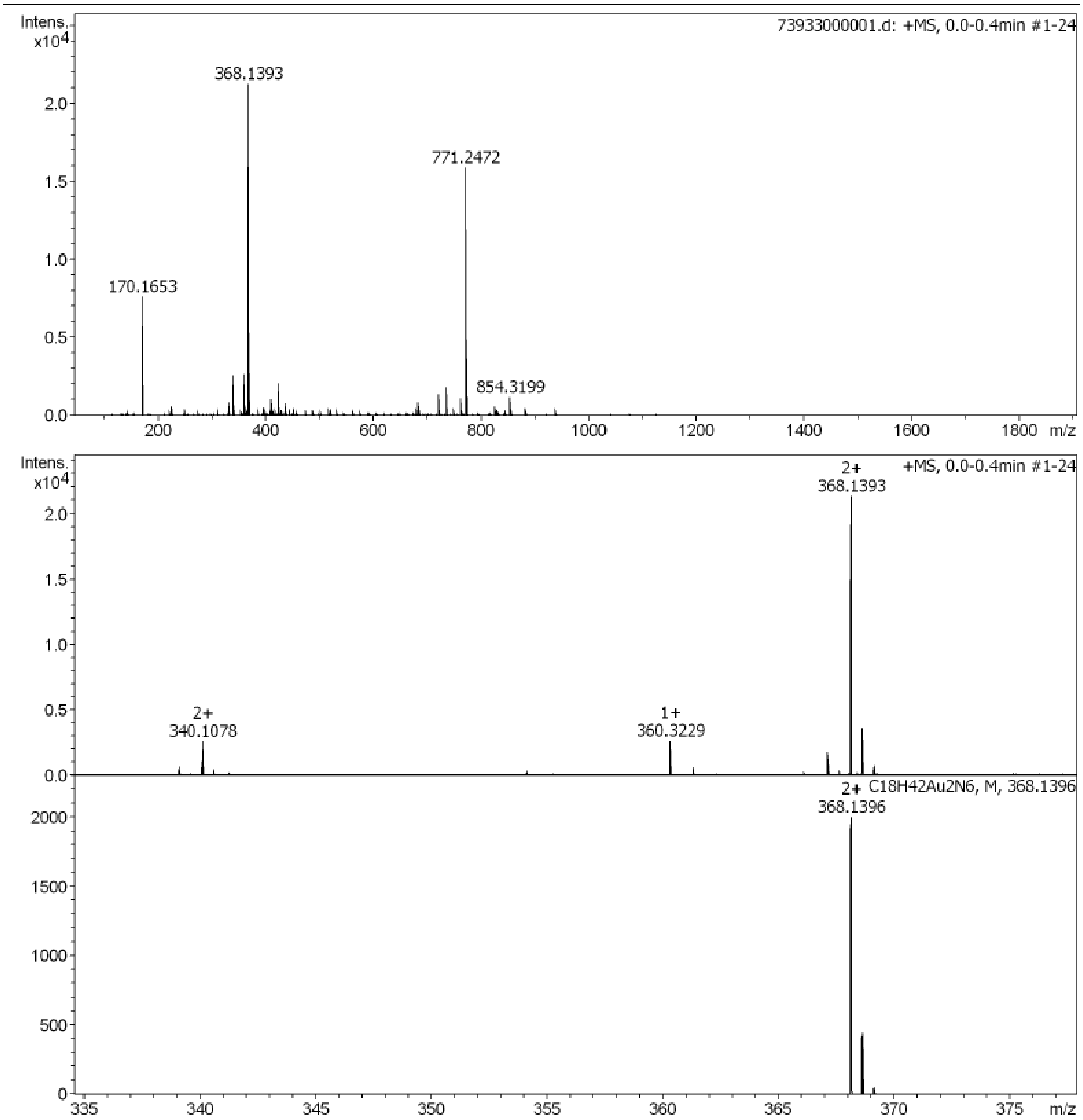

Figure S 28. Mass spectra of complex **3** $[M\text{-}2Cl^-]^{2+}$

Mass spectra of complex 4

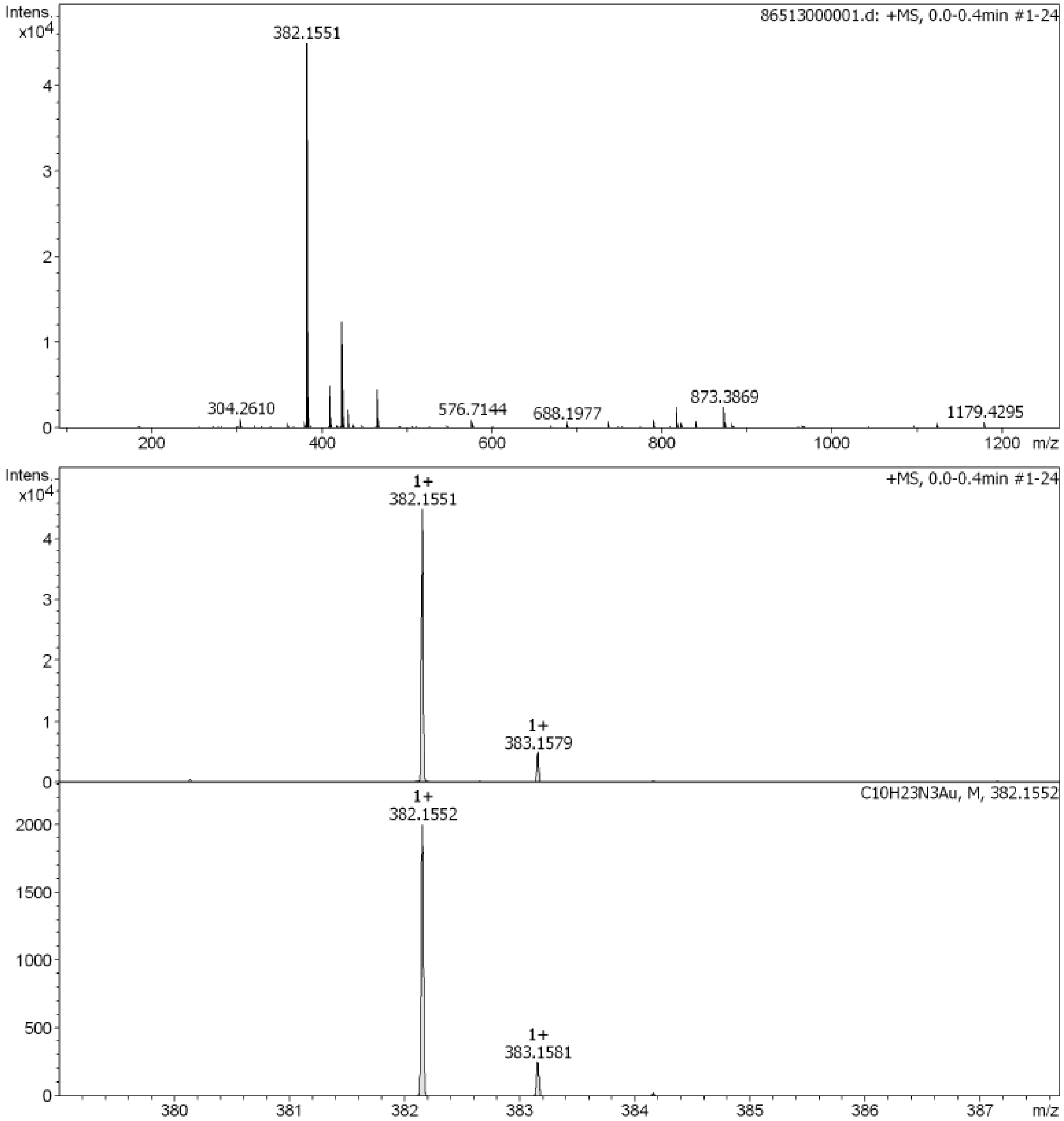

Figure S 29. Mass spectra of complex **4** [M-2Cl^{-}]$^{2+}$

Mass spectra of complex 5

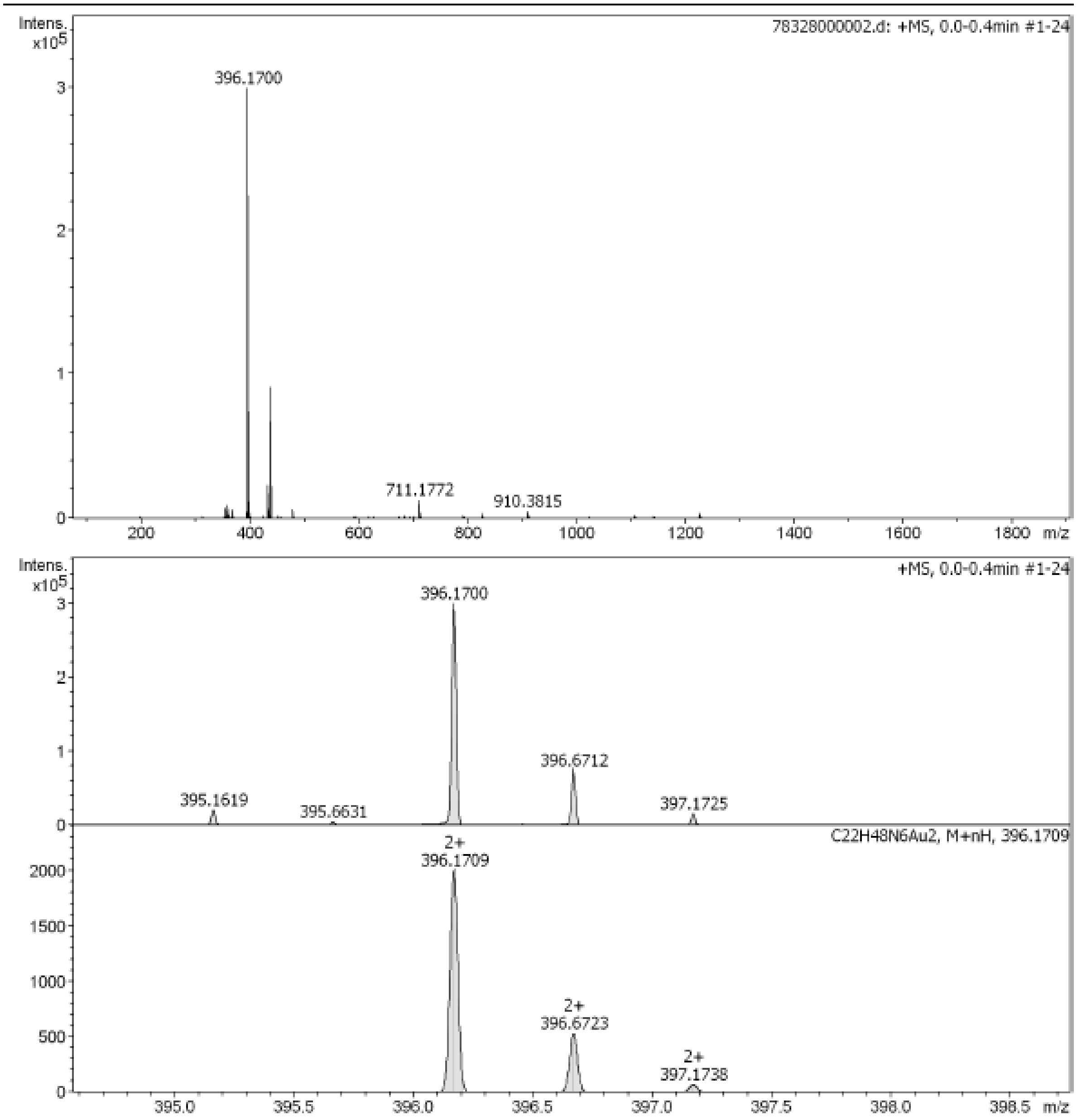

Figure S 30. Mass spectra of complex **5** $[M\text{-}2Cl^{-}]^{2+}$

Mass spectra of complex 6

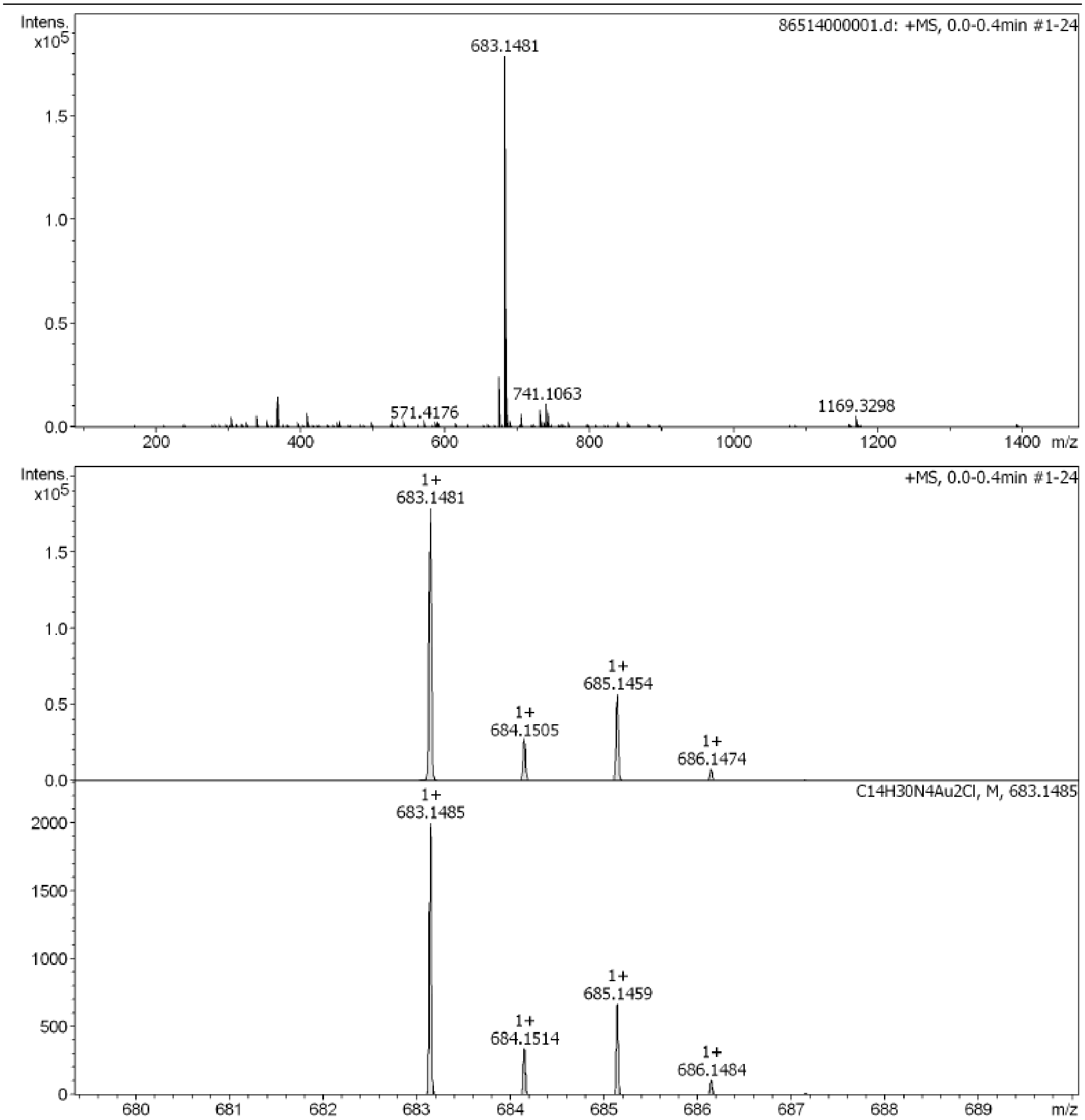

Figure S 31. Mass spectra of complex **6** $[M-Cl^-]^+$

Mass spectra of complex 7

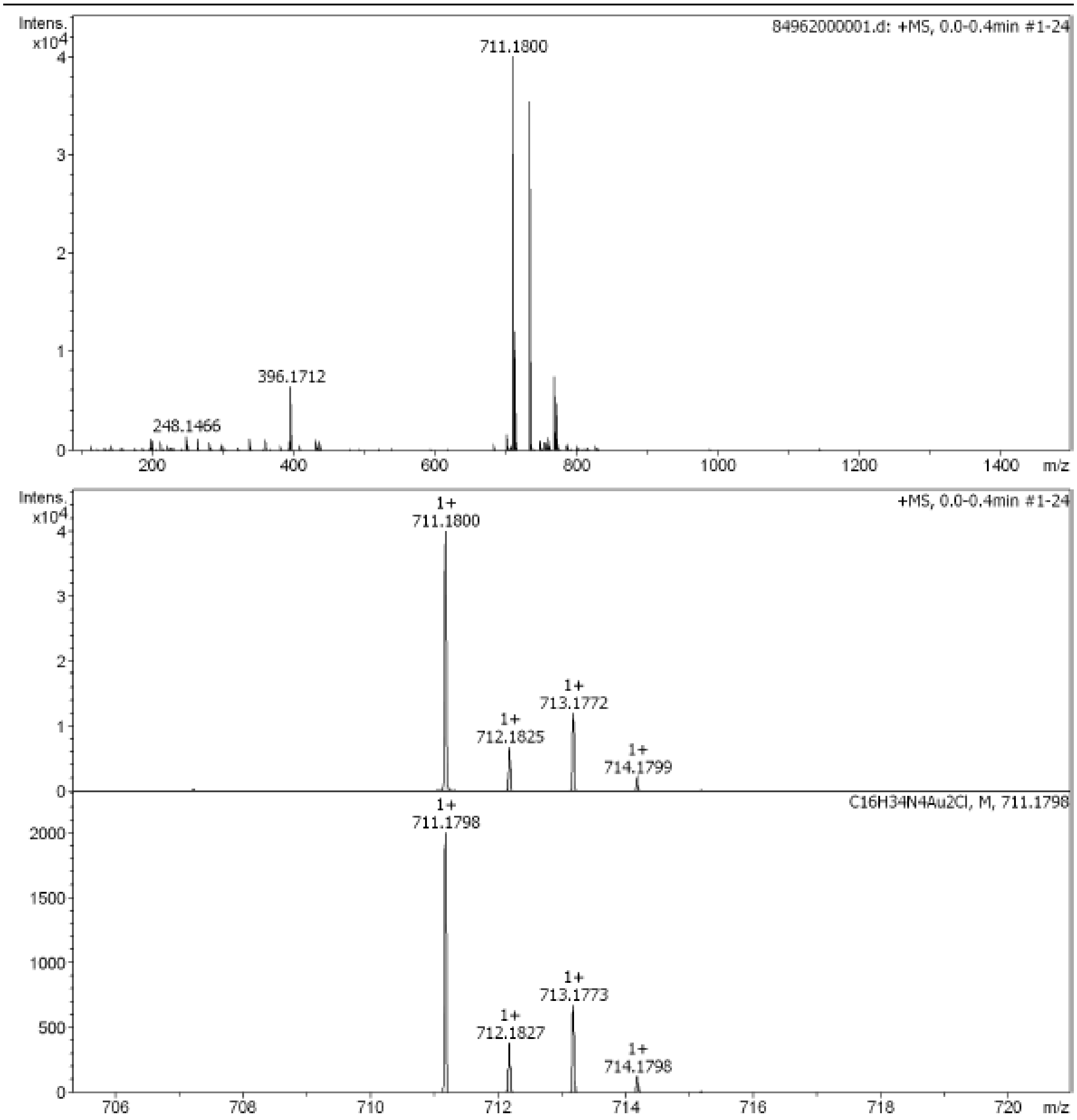

Figure S 32. Mass spectra of complex **7** $[M\text{-}Cl^-]^+$

Mass spectra of complex 8

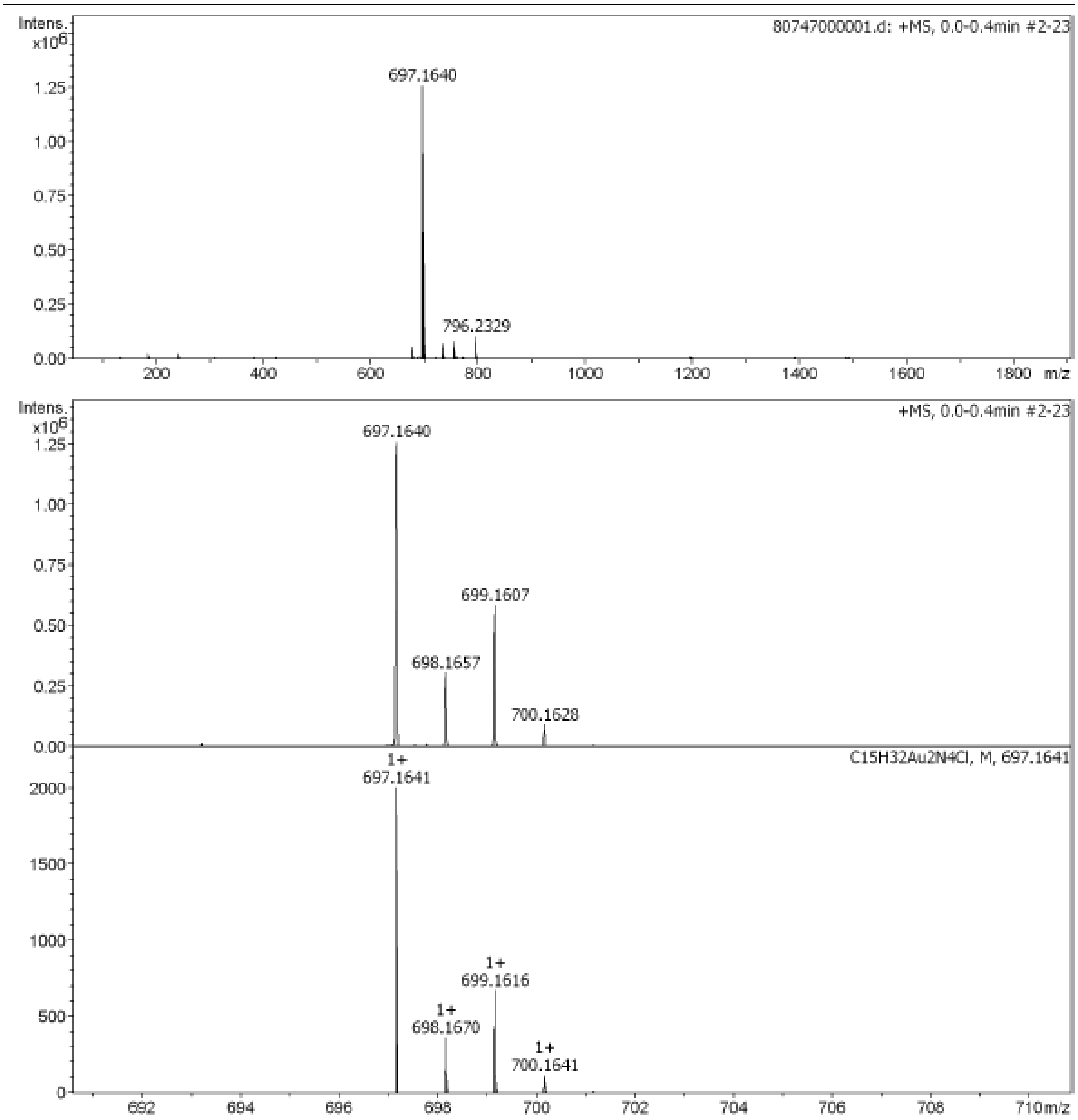

Figure S 33. Mass spectra of complex **8** $[M-Cl^{-}]^{+}$

Mass spectra of complex 9

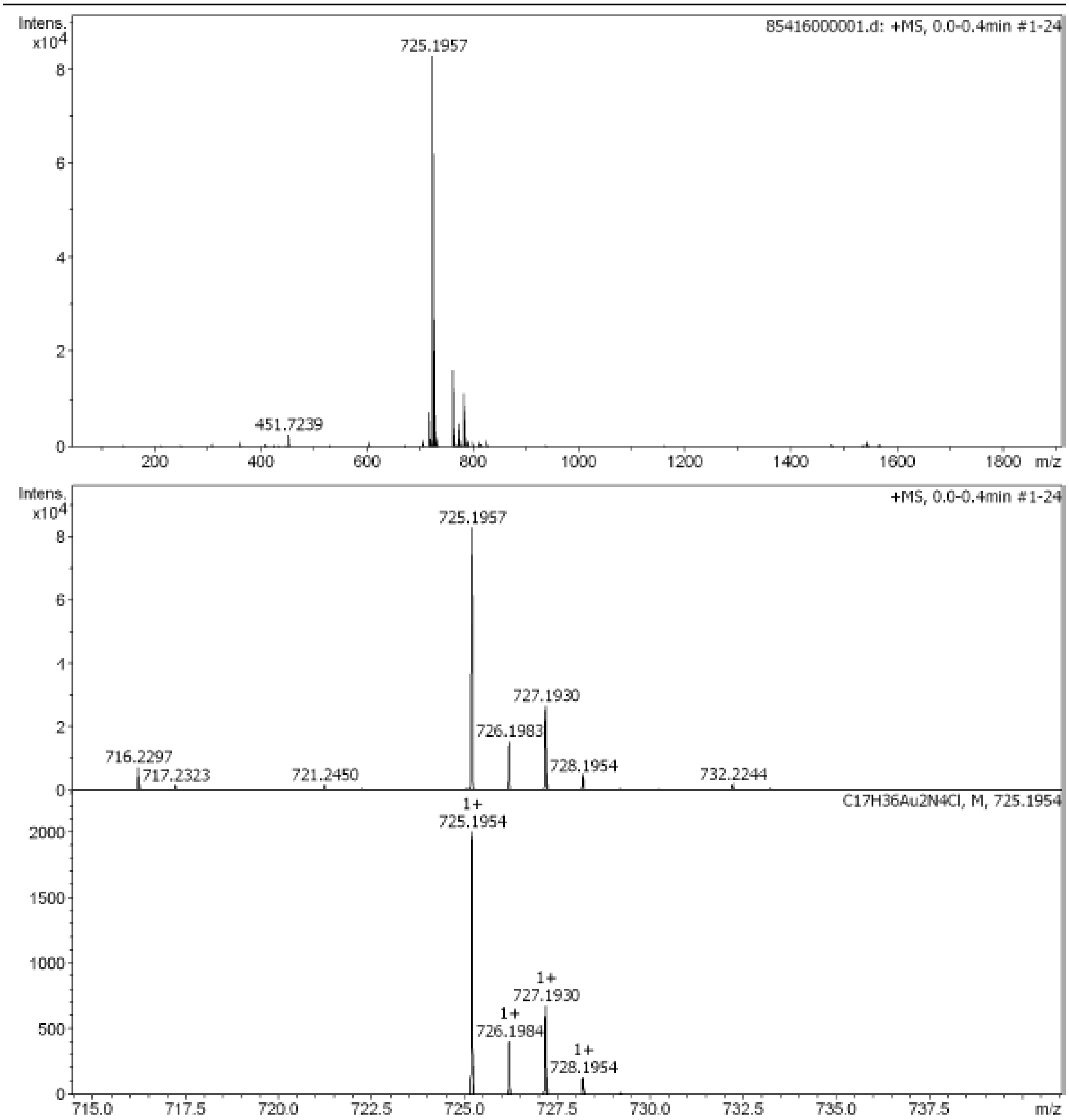

Figure S 34. Mass spectra of complex **9** $[M-Cl^{-}]^{+}$

Crystallographic data for complex 1

Single crystals of complex **1** were obtained by slow vapor diffusion of diethyl ether into an ethanol solution of the complex. The asymmetric unit contained half a molecule of **1** which is disordered around a special position.

CCDC number	2150201
Empirical formula	$C_{14}H_{34}Au_2Cl_2N_6$
Formula weight	751.30
Temperature/K	100.00
Crystal system	orthorhombic
Space group	Pccm
a/Å	5.6730(2)
b/Å	11.8653(6)
c/Å	18.9224(9)
α/°	90
β/°	90
γ/°	90
Volume/Å^3	1273.70(10)
Z	2
ρ_{calc}g/cm^3	1.959
μ/mm^{-1}	11.723
F(000)	704.0
Crystal size/mm^3	0.08 × 0.06 × 0.02
Radiation	MoKα (λ = 0.71073)
2Θ range for data collection/°	5.508 to 60.17
Index ranges	$-7 \leq h \leq 7$, $-16 \leq k \leq 16$, $-26 \leq l \leq 26$
Reflections collected	50259
Independent reflections	1932 [R_{int} = 0.0537, R_{sigma} = 0.0156]
Data/restraints/parameters	1932/0/109
Goodness-of-fit on F^2	1.149
Final R indexes [I>=2σ (I)]	R_1 = 0.0239, wR_2 = 0.0633
Final R indexes [all data]	R_1 = 0.0276, wR_2 = 0.0656
Largest diff. peak/hole / e Å^{-3}	1.10/-1.82

Crystallographic data for complex 3

Single crystals of complex **3** were obtained by slow vapor diffusion of diethyl ether into an ethanol solution of the complex. The asymmetric unit contained half a molecule of **3** and one molecule of ethanol

CCDC number	2150198
Empirical formula	$C_{22}H_{54}Au_2Cl_2N_6O_2$
Formula weight	899.54
Temperature/K	130.0
Crystal system	monoclinic
Space group	$P2_1/n$
a/Å	9.3277(5)
b/Å	10.2327(5)
c/Å	17.6647(7)
α/°	90
β/°	99.681(2)
γ/°	90
V/Å^3	1662.04(14)
Z	2
ρ_{calc} g/cm^3	1.797
μ/mm^{-1}	9.004
F(000)	872.0
Crystal size/mm^3	0.07 × 0.06 × 0.02
Wavelength	MoKα (λ = 0.71073)
2Θ range for data collection/°	4.618 to 50.694
Index ranges	$-11 \leq h \leq 11$, $-11 \leq k \leq 12$, $-21 \leq l \leq 21$
Reflections collected	16207
Independent reflections	3057 [R_{int} = 0.0369, R_{sigma} = 0.0232]
Data/restraints/parameters	3057/0/159
Goodness-of-fit on F^2	1.078
Final R indexes [I>=2σ (I)]	R_1 = 0.0207, wR_2 = 0.0530
Final R indexes [all data]	R_1 = 0.0242, wR_2 = 0.0545
Largest diff. peak/hole / e- ·Å^{-3}	1.41/-0.79

Crystallographic data for complex 6

Single crystals of complex **6** were obtained by slow vapor diffusion of diethyl ether into a dichloromethane solution of the complex. The asymmetric unit contained one molecule of **6**.

CCDC number	2150202
Empirical formula	$C_{14}H_{30}Au_2Cl_2N_4$
Formula weight	719.25
Temperature/K	100.0
Crystal system	monoclinic
Space group	$P2_1/c$

a/Å	12.6760(5)
b/Å	14.5418(11)
c/Å	11.5609(5)
α/°	90
β/°	108.991(2)
γ/°	90
V/Å^3	2015.05(19)
Z	4
ρ_{calc} g/cm^3	2.371
μ/mm^{-1}	14.811
F(000)	1336.0
Crystal size/mm^3	0.04 × 0.03 × 0.01
Wavelength	MoKα (λ = 0.71073)
2Θ range for data collection/°	4.404 to 50.694
Index ranges	$-15 \leq h \leq 15$, $-17 \leq k \leq 17$, $-13 \leq l \leq 12$
Reflections collected	17642
Independent reflections	3671 [R_{int} = 0.0760, R_{sigma} = 0.0571]
Data/restraints/parameters	3671/0/207
Goodness-of-fit on F^2	1.023
Final R indexes [I>=2σ (I)]	R_1 = 0.0295, wR_2 = 0.0682
Final R indexes [all data]	R_1 = 0.0373, wR_2 = 0.0710
Largest diff. peak/hole / e- ·Å^{-3}	0.94/-1.04

Crystallographic data for complex 7

Single crystals of complex **7** were obtained by slow vapor diffusion of diethyl ether into a dichloromethane solution of the complex. The asymmetric unit contained two crystallographically independent half molecules of **7** and one molecule of dichloromethane.

CCDC number	2150199
Empirical formula	$C_{17}H_{36}Au_2Cl_4N_4$
Formula weight	832.23
Temperature/K	100.0
Crystal system	triclinic
Space group	P-1
a/Å	9.2153(3)
b/Å	12.1047(4)
c/Å	12.8836(4)
α/°	67.5730(10)
β/°	73.5160(10)

γ/°	82.4420(10)
V/Å^3	1273.41(7)
Z	2
ρ_{calc} g/cm^3	2.170
μ/mm^{-1}	11.938
F(000)	784.0
Crystal size/mm^3	0.2 × 0.07 × 0.01
Wavelength	MoKα (λ = 0.71073)
2Θ range for data collection/°	3.642 to 66.604
Index ranges	-13 ≤ h ≤ 14, -18 ≤ k ≤ 18, -19 ≤ l ≤ 19
Reflections collected	76128
Independent reflections	9785 [R_{int} = 0.0499, R_{sigma} = 0.0313]
Data/restraints/parameters	9785/0/252
Goodness-of-fit on F^2	1.025
Final R indexes [I>=2σ (I)]	R_1 = 0.0253, wR_2 = 0.0510
Final R indexes [all data]	R_1 = 0.0359, wR_2 = 0.0546
Largest diff. peak/hole / e- ·Å^{-3}	1.47/-1.46

Crystallographic data for complex 9

Single crystals of complex **9** were obtained by slow vapor diffusion of diethyl ether into a dichloromethane solution of the complex. The asymmetric unit contained three crystallographically independent molecules of **9**.

CCDC number	2150200
Empirical formula	$C_{17}H_{36}Au_2Cl_2N_4$
Formula weight	761.33
Temperature/K	100.0
Crystal system	monoclinic
Space group	$P2_1/c$
a/Å	20.9778(5)
b/Å	10.5387(2)
c/Å	31.7375(6)
α/°	90
β/°	91.8102(11)
γ/°	90
V/Å^3	7013.0(2)
Z	12
ρ_{calc} g/cm^3	2.163
μ/mm^{-1}	12.774

$F(000)$	4296.0
Crystal size/mm^3	0.2 × 0.1 × 0.01
Wavelength	MoKα (λ = 0.71073)
2Θ range for data collection/°	3.886 to 60.804
Index ranges	$-29 \leq h \leq 29$, $-11 \leq k \leq 14$, $-45 \leq l \leq 45$
Reflections collected	157570
Independent reflections	21013 [R_{int} = 0.1195, R_{sigma} = 0.0809]
Data/restraints/parameters	21013/0/700
Goodness-of-fit on F^2	1.008
Final R indexes [I>=2σ (I)]	R_1 = 0.0407, wR_2 = 0.0673
Final R indexes [all data]	R_1 = 0.0857, wR_2 = 0.0782
Largest diff. peak/hole / e- ·Å^{-3}	1.51/-2.04

DFT calculations

Optimized molecular structures of the intermediates and transition states investigated by DFT calculations.

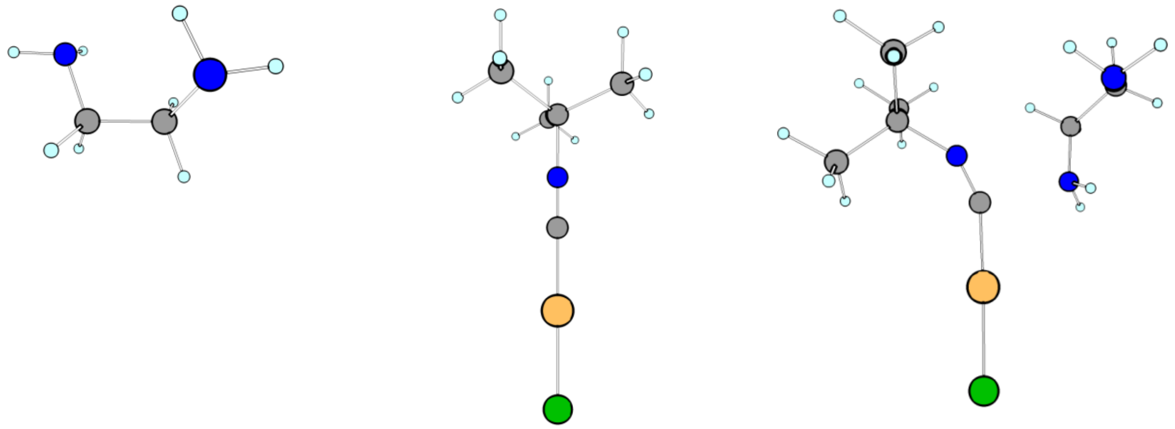

Figure S 35. **Ethylene diamine** (left), [**AuCNtBuCl**] (center), A1 (right)

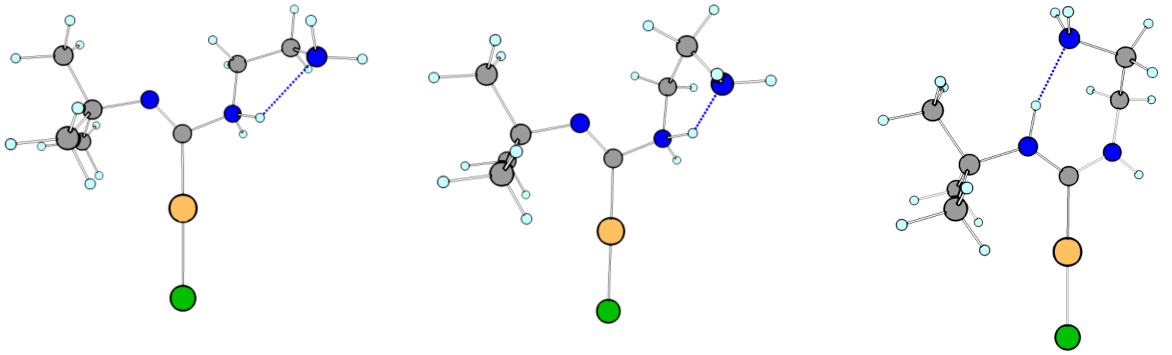

Figure S 36. **B1** (left), **C1** (center), **D1** (right)

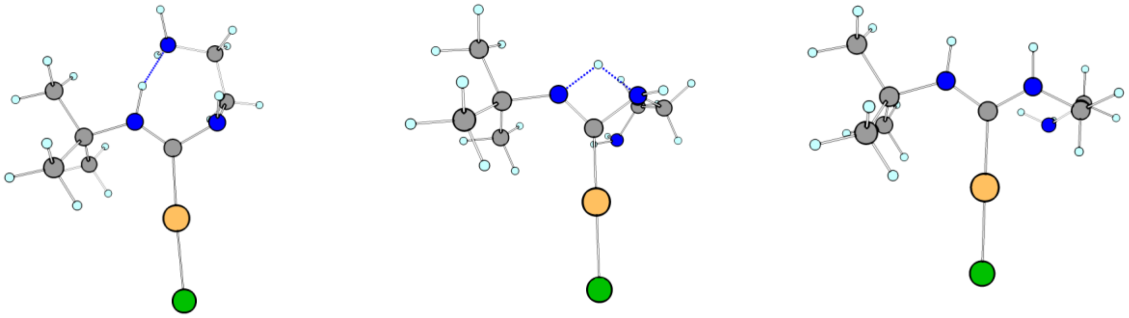

Figure S 37. **E1** (left), **F1** (center), **G1** (right)

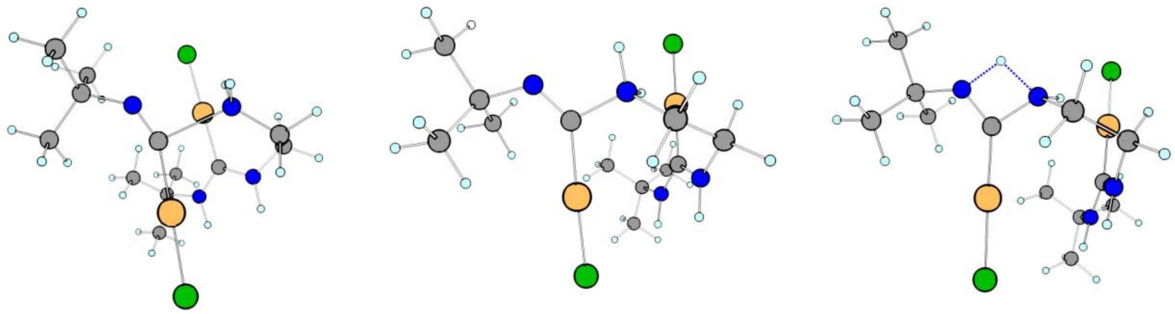

Figure S 38. **H1** (left), **I1** (center), **J1** (right)

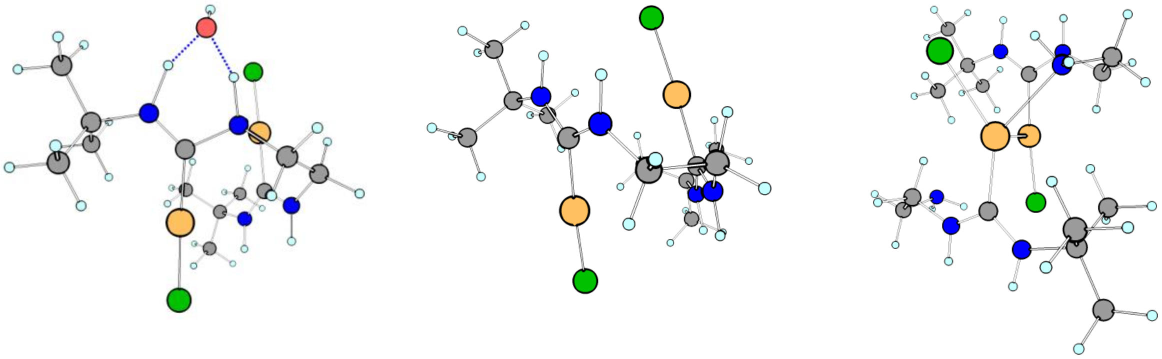

Figure S 39. **K1** (left), **L1** (center), **M1** (right)

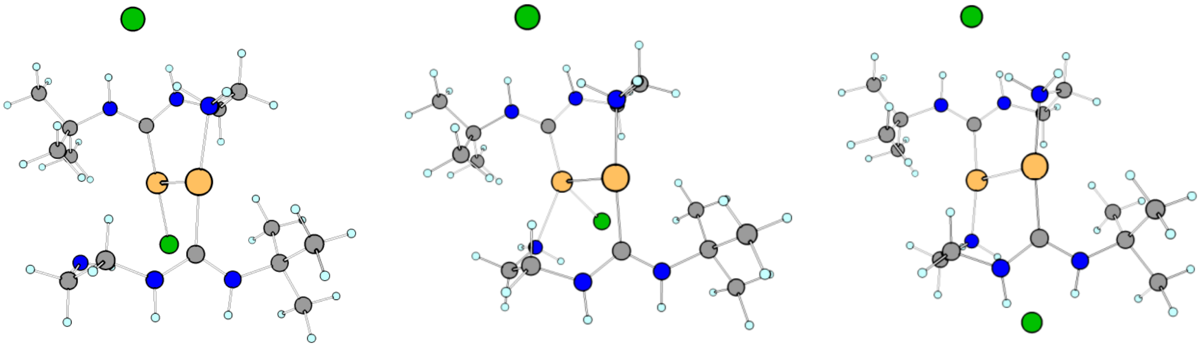

Figure S 40. **N1** (left), **O1** (center), **P1** (right)

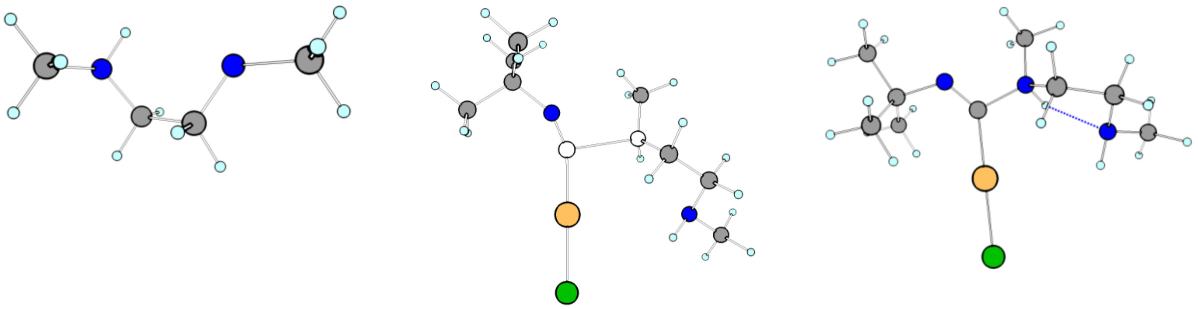

Figure S 41. **1,2-Dimethylethylenediamine** (left), **A2** (center), **B2** (right)

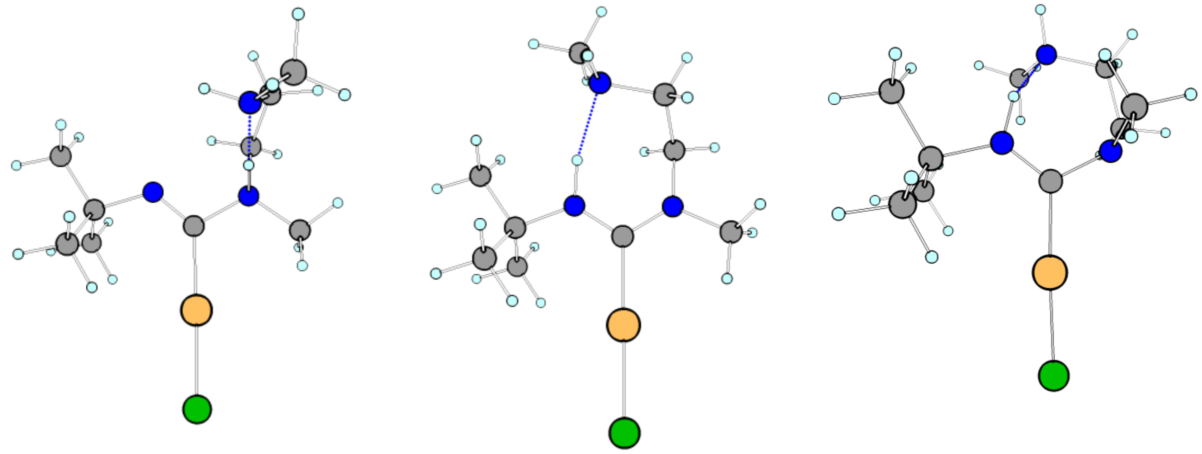

Figure S 42. **C2** (left), **D2** (center), **E2** (right)

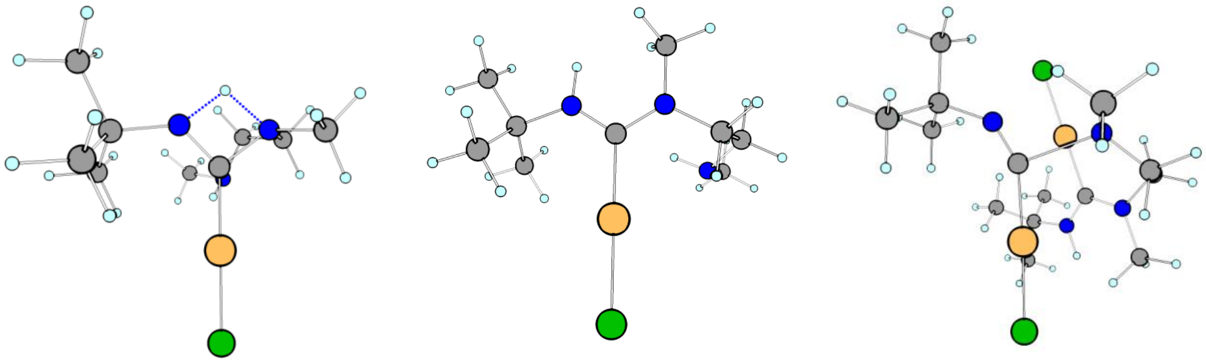

Figure S 43. **F2** (left), **G2** (center), **H2** (right)

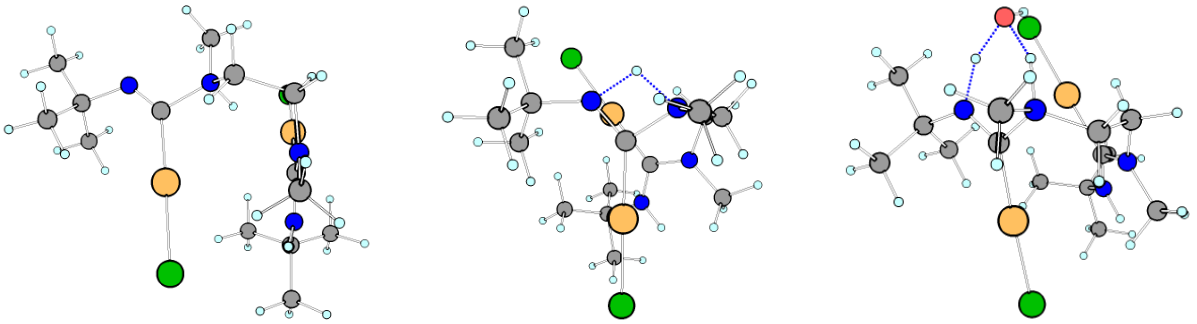

Figure S 44. **I2** (left), **J2** (center), **K2** (right)

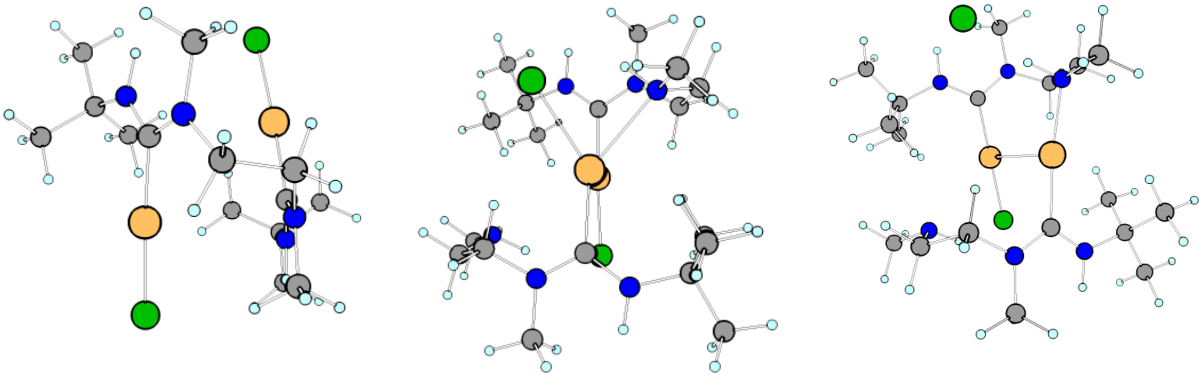

Figure S 45. **L2** (left), **M2** (center), **N2** (right)

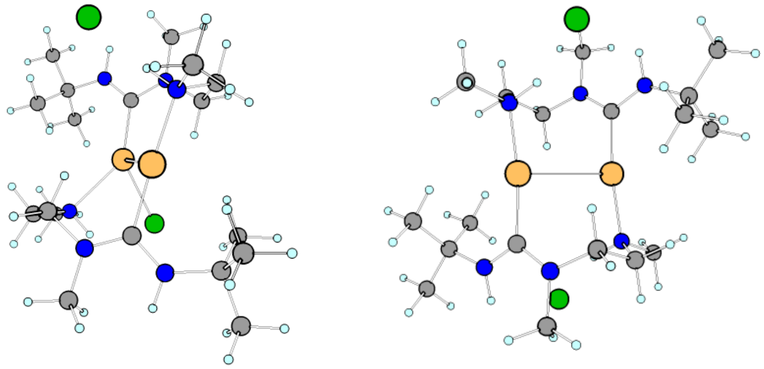

Figure S 46. **O2** (left), **P2** (right)

Table S 1. DFT-calculated electronic energies and Gibbs free energies after geometry optimization with BP86 D3/def2 TZVP, single point energies calculated with B2PLYP-D3/def2 TZVP

	E_{el} (BP86-D3) (E_h)	G (BP86-D3) (E_h)	E_{el} (B2PLYP-D3) (E_h)	G_{solv}(CPCM) kcal/mol	ΔG_{solv} kcal/mol
**H_2O**	-76.47442212	-76.47161952	-76.41733672	-47950.85435	
**AuCN*t*BuCl (SM1)**	-847.0512607	-846.9600623	-846.2886381	-530997.0237	
**Ethylene Diamine (SM2)**	-190.6196226	-190.5404164	-190.4359159	-119450.6643	
**SM1 + SM2**	-1037.670883	-1037.500479	-1036.724554	-650447.688	0.0
**A1**	-1037.664706	-1037.472491	-1036.708138	-650423.701	24.0
**B1**	-1037.681348	-1037.48473	-1036.723427	-650430.5319	17.2
**C1**	-1037.679132	-1037.484715	-1036.716194	-650427.3744	20.4
**D1**	-1037.718579	-1037.520988	-1036.759349	-650452.4621	-4.8
**E1**	-1037.684427	-1037.487461	-1036.724562	-650431.0261	16.7
**F1**	-1037.644379	-1037.452725	-1036.681187	-650407.1408	40.6
**G1**	-1037.715079	-1037.518198	-1036.757458	-650451.7217	-4.1
**H1**	-1884.773651	-1884.459304	-1883.038653	-1181427.591	17.2
**I1**	-1884.786835	-1884.469067	-1883.050499	-1181432.878	11.9
**J1**	-1884.751963	-1884.439605	-1883.01065	-1181411.268	33.5
**K1**	-1961.265948	-1960.932232	-1959.461647	-1229371.6	24.0
**L1**	-1884.826647	-1884.507866	-1883.091793	-1181458.155	-13.5

**M1**	-2075.444522	-2075.023501	-2073.519688	-1300889.331	10.1
**N1**	-2075.469441	-2075.046667	-2073.545783	-1300904.607	-5.2
**O1**	-2075.45859	-2075.033591	-2073.532937	-1300895.149	4.3
**P1**	-2075.47827	-2075.052334	-2075.052334	-1300907.315	-8.0
**Dimethylethylene Diamine (SM3)**	-269.2535768	-269.1243676	-268.9873984	-168713.1778	
**SM1 + SM3**	-1116.304837	-1116.08443	-1115.276036	-699710.2015	0.0
**A2**	-1116.308503	-1116.065615	-1115.269771	-699690.0824	20.2
**B2**	-1116.32652	-1116.32652	-1115.28609	-699697.0825	13.2
**C2**	-1116.318931	-1116.073846	-1115.273603	-699691.1081	19.1
**D2**	-1116.359349	-1116.110853	-1115.316613	-699715.9567	-5.8
**E2**	-1116.326566	-1116.077392	-1115.283915	-699695.0135	15.2
**F2**	-1116.29142	-1116.04868	-1115.245518	-699674.9562	35.3
**G2**	-1116.357313	-1116.109296	-1115.316713	-699716.3205	-6.2
**H2**	-1963.421058	-1963.054356	-1961.602705	-1230694.436	12.8
**I2**	-1963.433567	-1963.064605	-1961.614403	-1230700.358	6.9
**J2**	-1963.399446	-1963.034137	-1961.575828	-1230678.444	28.8
**K2**	-2039.915181	-2039.52865	-2038.02866	-1278640.014	18.1
**L2**	-1963.473871	-1963.103439	-1961.654694	-1230724.718	-17.5
**M2**	-2232.733635	-2232.209331	-2230.641411	-1399419.912	6.7
**N2**	-2232.754466	-2232.228438	-2230.66466	-1399433.418	-6.9
**O2**	-2232.746196	-2232.218727	-2230.655592	-1399426.824	-0.4
**P2**	-2232.762648	-2232.233791	-2230.673925	-1399437.457	-11.0

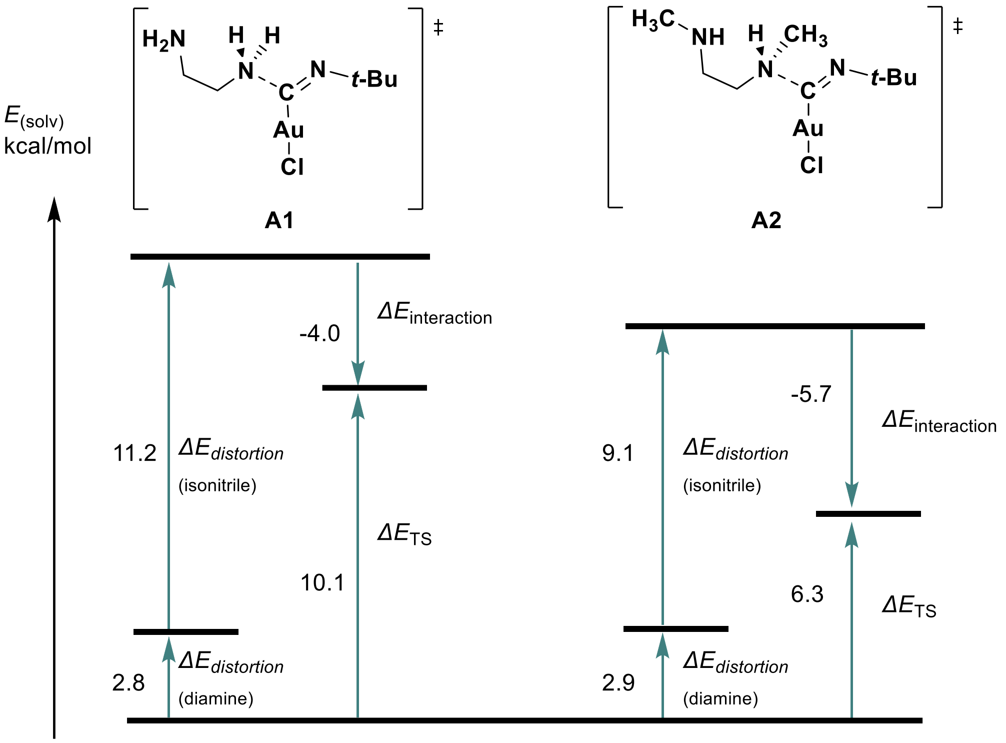

Figure S 47. Distortion-interaction analysis of transition states **A1** and **A2**

Distortion-interaction analysis of the two transition states (Figure S46) revealed that while the distortion energy $\Delta E_{\text{distortion}}$ of both diamines in were similar (2.8 and 2.9 kcal/mol in **A1** and **A2** respectively), $\Delta E_{\text{distortion}}$ of the isonitrile complex in **A2** (9.1 kcal/mol) was lower than $\Delta E_{\text{distortion}}$ of the isonitrile complex in **A1** (11.2 kcal/mol). In addition, the stronger interaction between the secondary amine and the isonitrile (-5.7 kcal/mol) was more stabilizing than the interaction between the less basic primary amine and isonitrile in A1 (-4.0 kcal/mol).

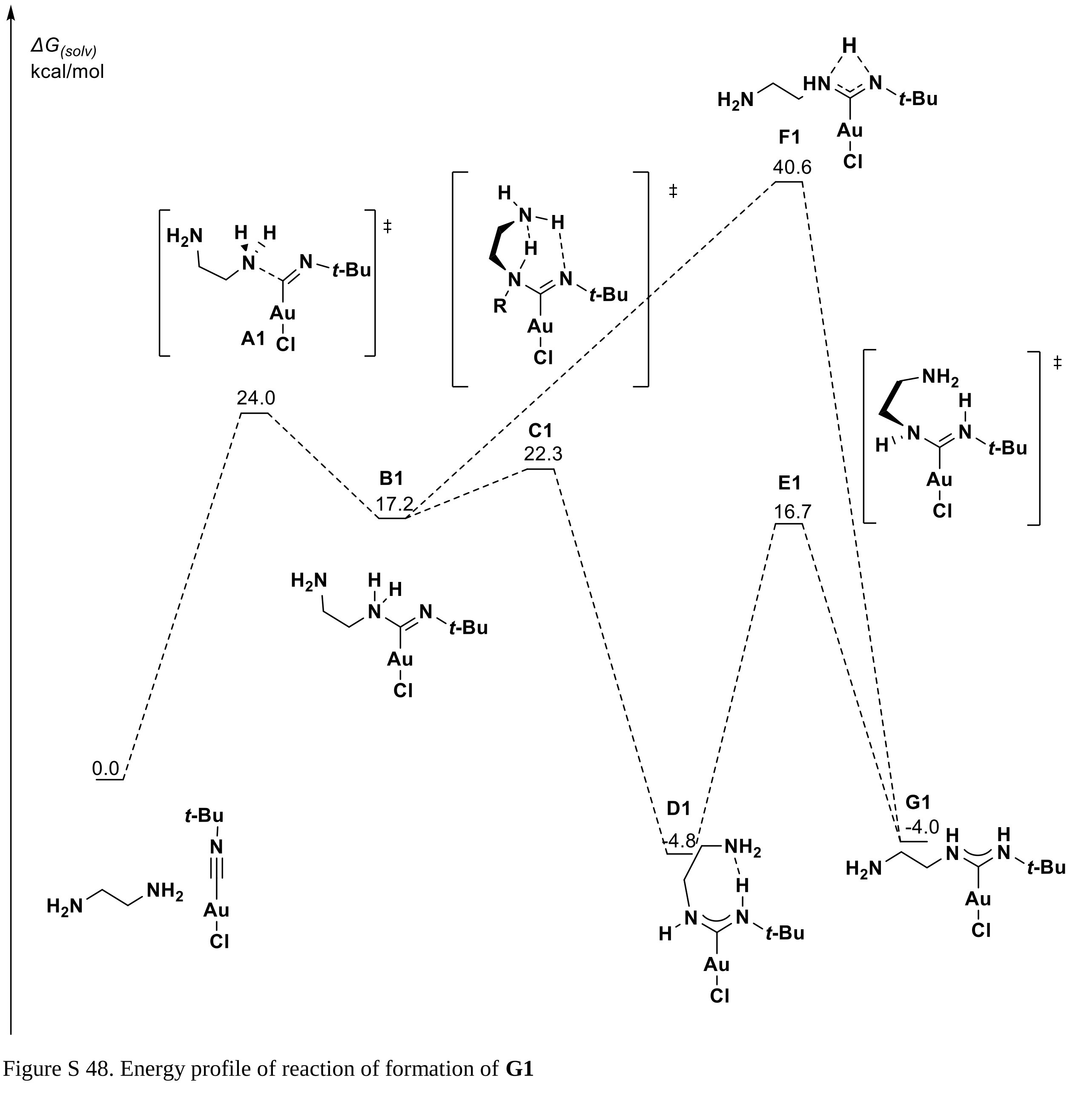

Figure S 48. Energy profile of reaction of formation of **G1**

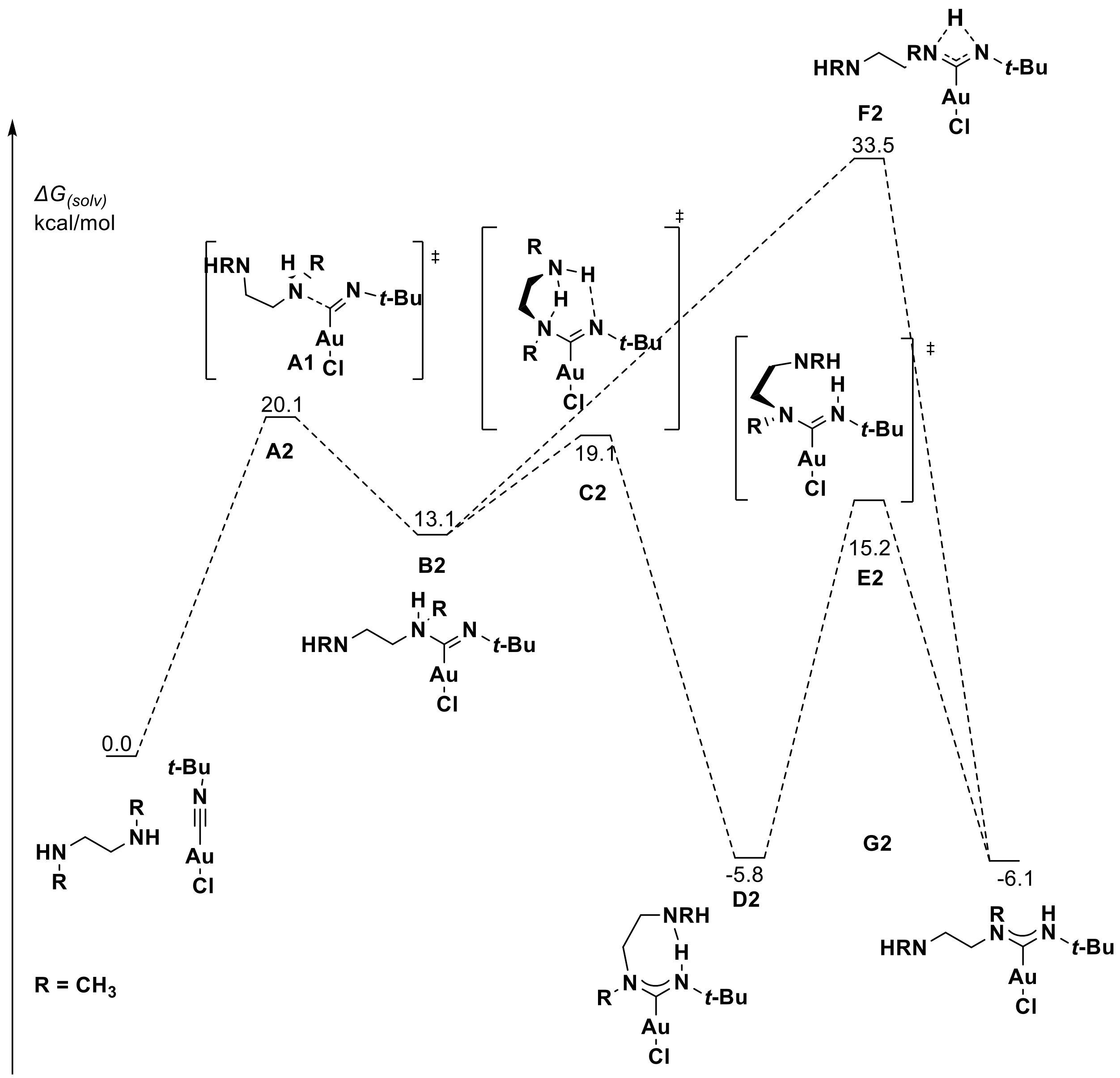

Figure S 49. Energy profile of reaction of formation of **G2**

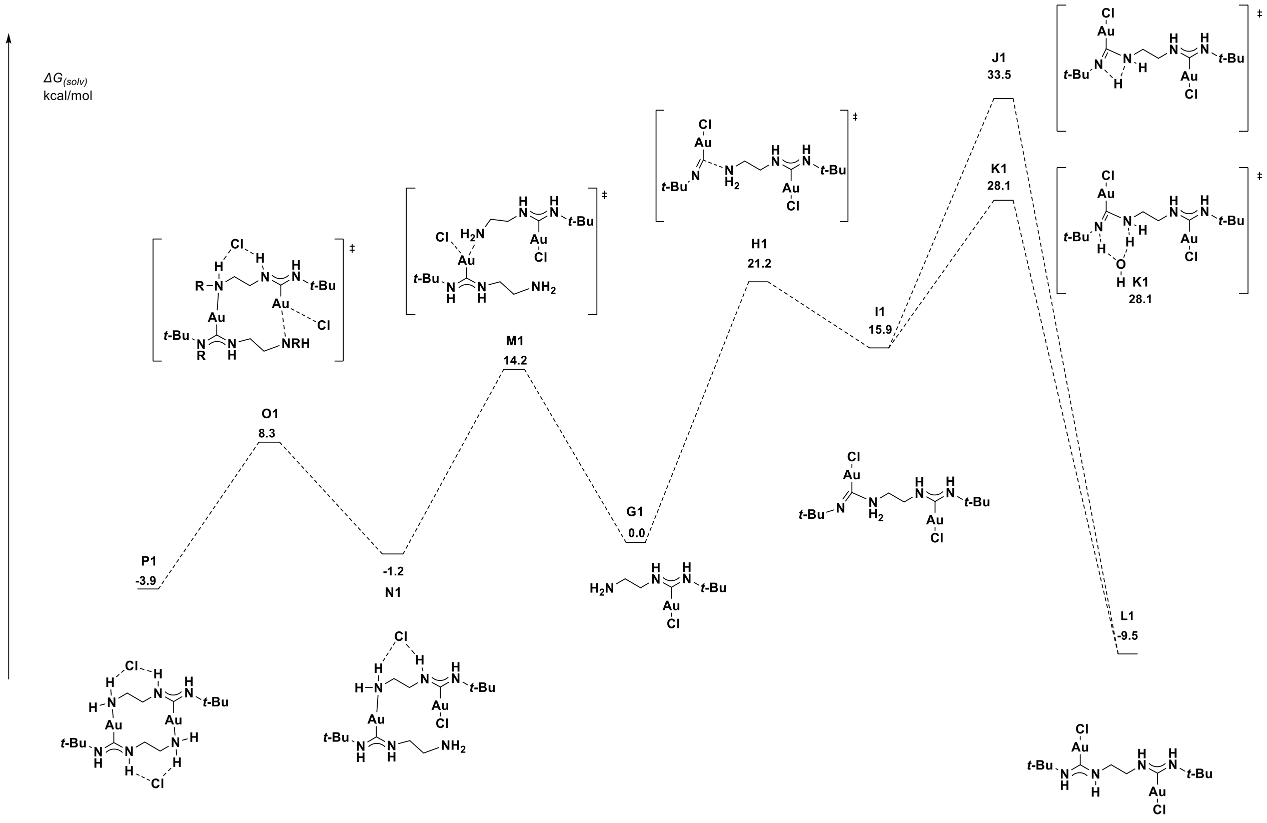

Figure S 50. Energy profile of reaction of reactivity of **G1** to form either the bis carbene complex **L1** or the dimer complex **P1.**

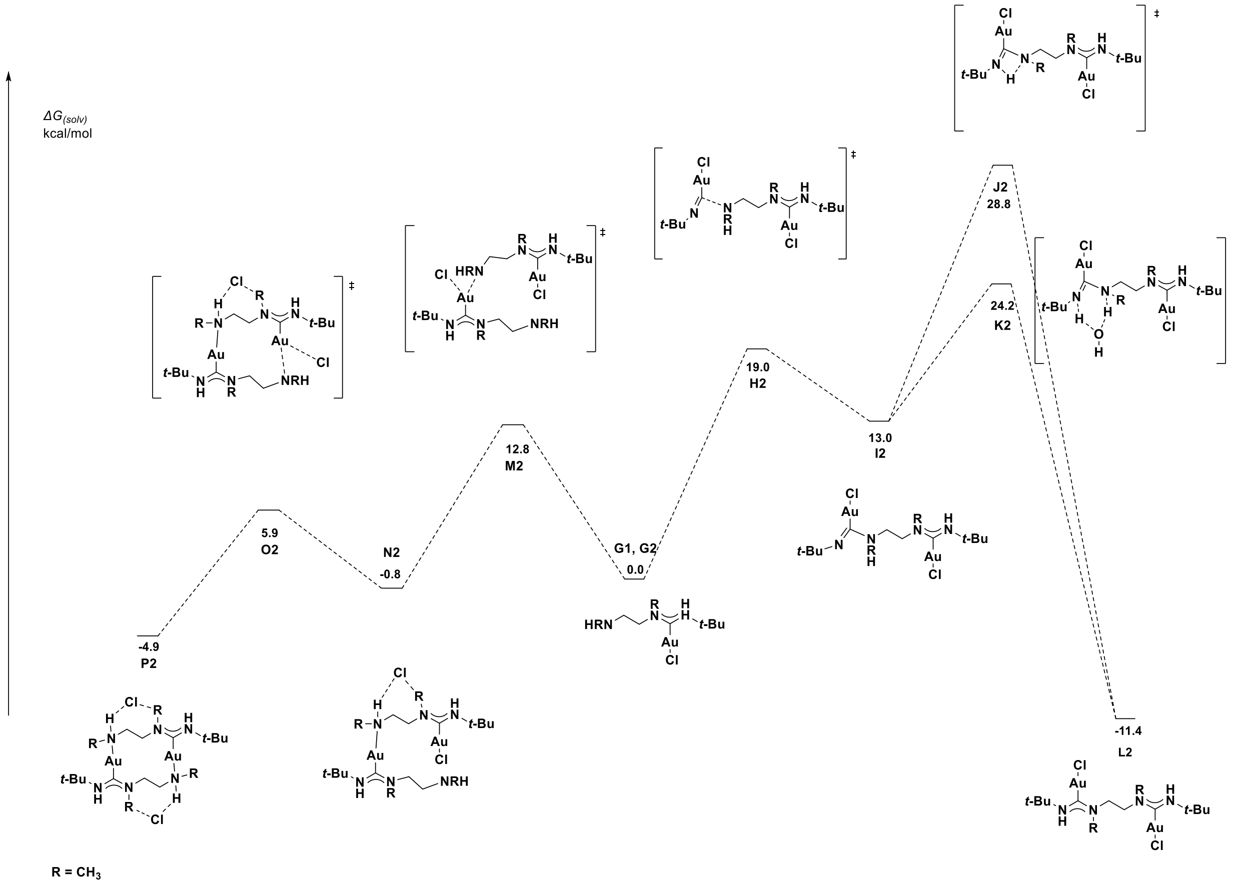

Figure S 51. Energy profile of reaction of reactivity of **G2** to form either the bis carbene complex **L2** or the dimer complex **P2.**
